# Network Analysis of Breast Cancer Progression and Reversal Using a Tree-Evolving Network Algorithm

**DOI:** 10.1371/journal.pcbi.1003713

**Published:** 2014-07-24

**Authors:** Ankur P. Parikh, Ross E. Curtis, Irene Kuhn, Sabine Becker-Weimann, Mina Bissell, Eric P. Xing, Wei Wu

**Affiliations:** 1 Machine Learning Department, School of Computer Science, Carnegie Mellon University, Pittsburgh, Pennsylvania, United States of America; 2 Lane Center for Computational Biology, School of Computer Science, Carnegie Mellon University, Pittsburgh, Pennsylvania, United States of America; 3 Life Sciences Division, Lawrence Berkeley National Laboratory, Berkeley, California, United States of America; 4 Joint Carnegie Mellon University-University of Pittsburgh PhD Program in Computational Biology, Pittsburgh, Pennsylvania, United States of America; Memorial Sloan-Kettering Cancer Center, United States of America

## Abstract

The HMT3522 progression series of human breast cells have been used to discover how tissue architecture, microenvironment and signaling molecules affect breast cell growth and behaviors. However, much remains to be elucidated about malignant and phenotypic reversion behaviors of the HMT3522-T4-2 cells of this series. We employed a “pan-cell-state” strategy, and analyzed jointly microarray profiles obtained from different state-specific cell populations from this progression and reversion model of the breast cells using a tree-lineage multi-network inference algorithm, *Treegl*. We found that different breast cell states contain distinct gene networks. The network specific to non-malignant HMT3522-S1 cells is dominated by genes involved in normal processes, whereas the T4-2-specific network is enriched with cancer-related genes. The networks specific to various conditions of the reverted T4-2 cells are enriched with pathways suggestive of compensatory effects, consistent with clinical data showing patient resistance to anticancer drugs. We validated the findings using an external dataset, and showed that aberrant expression values of certain hubs in the identified networks are associated with poor clinical outcomes. Thus, analysis of various reversion conditions (including non-reverted) of HMT3522 cells using *Treegl* can be a good model system to study drug effects on breast cancer.

## Author Summary

The HMT3522 isogenic human breast cancer progression series has been used to study the effect of various drugs on the reversion of the breast cancer cells. Despite significant efforts to delineate key signaling events responsible for phenotypic reversion of the malignant HMT3522-T4-2 (T4-2) breast cells in this series, many questions remain. For example, what is involved in the phenotypic reversion of T4-2 cells at the systems level? In order to answer this question, we analyzed gene expression microarray data obtained from these cells using our recently developed tree-evolving network inference algorithm *Treegl*. We reconstructed cell-state-specific gene networks using *Treegl*. Our functional analysis results show that we can not only unravel cell-state specific information characteristic of non-malignant HMT3522-S1 (S1) and malignant T4-2 cells in the series, but can also provide insight into the T4-2 cells reverted by various agents. We found that the networks specific to various conditions of the T4-2 reverted cells are all suggestive of compensatory signaling effects, which, however, are mediated by different signaling pathways to antagonize different drug effects in the reverted cells. Our results demonstrate that the HMT3522 system when analyzed with *Treegl* may potentially become an effective tool for novel drug-target discovery and identification.

## Introduction

A major challenge in systems biology is to uncover dynamic changes in cellular pathways that either respond to the changing microenvironment of cells, or drive cellular transformation during various biological processes such as cell cycle, differentiation, and development. These changes may involve rewiring of transcriptional regulatory circuitry or signal transduction pathways that control cellular behaviors. Such information is of particular importance for seeking a deep mechanistic understanding of cellular responses to drug treatments in various diseases, offering a more holistic view of both microscopic and macroscopic changes in the cellular functional machinery than has been available from traditional analyses which usually focus only on finding differential markers or close-up analysis of changes in a handful of molecules constituting parts of some selected pathways of interest.

Network-based differential analysis naturally requires the availability of multiple networks each in principle corresponding to a specific biological condition in question, that are then topologically rewired across conditions [1]. However, most existing computational techniques for reconstructing molecular networks based on high-throughput data cannot capture such dynamic aspects of the network topology; instead, they represent the networks as an invariant graph. For example, it is common to infer a single invariant gene network using microarray data obtained from samples collected over time or multiple conditions. More sophisticated methods such as a trace-back algorithm [1] and DREM [2], [3] do emphasize uncovering the dynamic changes of a network over time using time series data, but limitations in these algorithms allow only certain kinds of dynamic behaviors, such as “active path” [1] or bifurcating sequence of transcriptional activations [2]. Moreover, such methods are heuristic in nature and do not offer statistical guarantees on the asymptotic correctness of the inferred “transient” components in the network, making the results difficult to withstand the harsh standard on stability and robustness when sample quality and size become less ideal, as we face in the analysis to be conducted in this paper.

**Figure 1:**
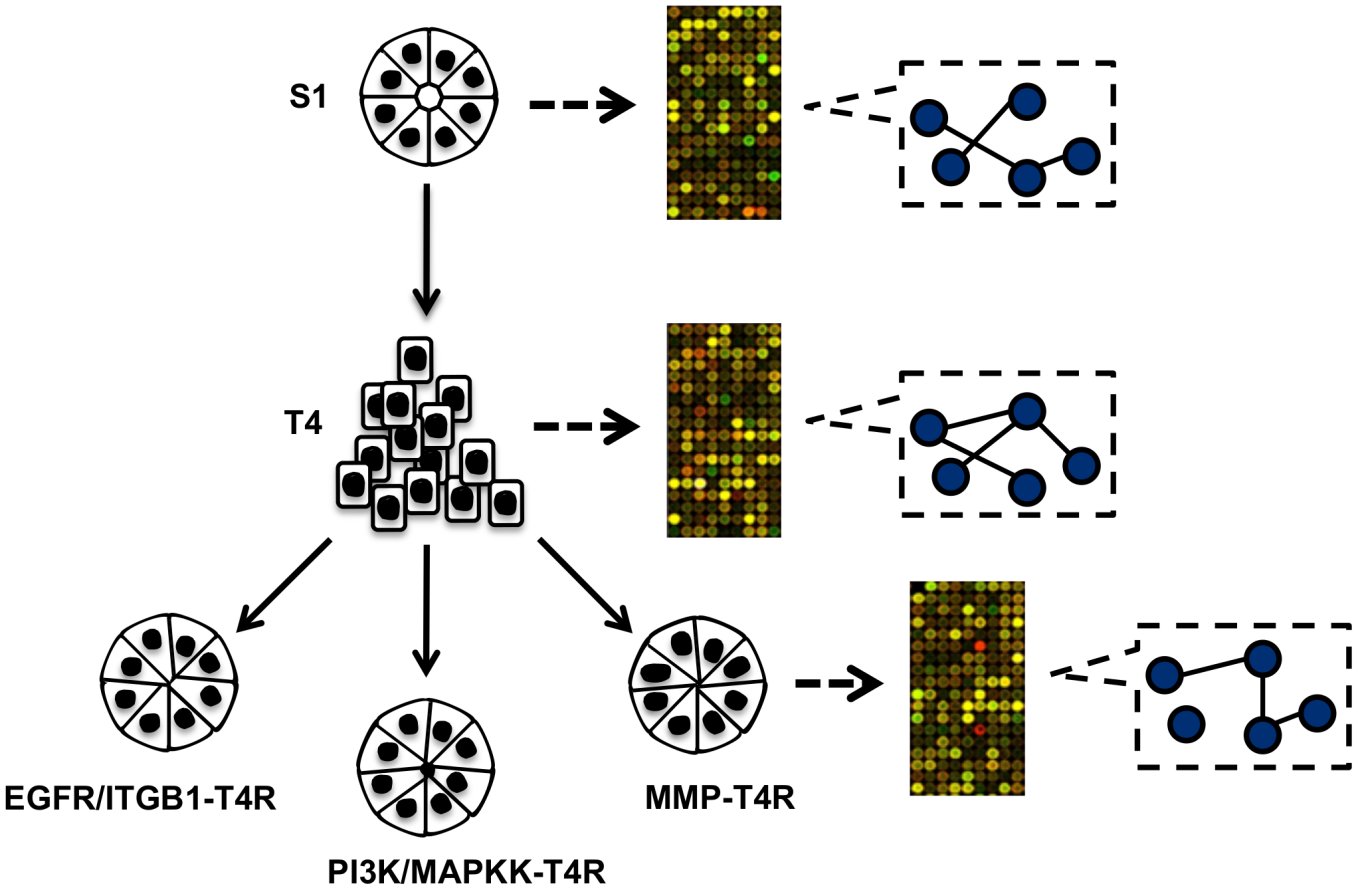
A schematic representation of the relationship of the non-reverted and various conditions of the reverted HMT3522 breast cells. The nonmalignant S1 cell is the root of the tree. It is also the parent of the malignant T4-2 cell since T4-2 cells were derived from S1. The T4-2 cells can be reverted to phenotypically normal-looking structures by treatment with various agents, such as: i) either EGFR or β1-integrin inhibitor, ii) either PI3K or MAPKK inhibitor, or iii) MMP inhibitors, they are thus represented as the parent of the various conditions of the reverted T4-2 cells. Microarray profiles were generated from each cell state represented in the tree, and gene networks specific to each state were reverse engineered using *Treegl*.

Indeed, a number of in-depth investigations of disease models have suggested that over the course of cellular transformation in response to microenvironmental changes due to disease progression or drug-induced reversion, there may exist multiple underlying “themes” that determine each molecule's function and relationship with other molecules [4], [5]. As a result, molecular networks at each cellular stage are context-dependent and can undergo systematic rewiring (Figure 1). For example, strong evidence of alterations of various pathways have been reported in the HMT3522 progression series of breast cells when malignant T4-2 cells were phenotypically reverted by various drugs, albeit only manifested by a small number of well-known signaling molecules as discussed below [6]–[8].

In this paper, we conduct an in-depth study of the structural changes in the gene regulatory networks underlying each cell state in both the non-reverted and the reverted HMT3522 progression series of breast cells. The HMT3522 cells have been shown to be an excellent model system for studying the roles of tissue architecture, microenvironment and signaling molecules involved in the nonmalignant and malignant growth and behaviors of breast cells, including the potential of various factors to cause phenotypic reversion of malignant cells to nonmalignant states. These cells originated from a nonmalignant human breast epithelial sample, HMT3522 [9], [10]. HMT3522-S1_LBNL (S1) cells are from early passages which are nonmalignant and dependent on exogenous epidermal growth factor (EGF) to grow. HMT3522-T4-2_LBNL (T4-2) cells were generated from S1 cells by a multi-step process: 238 passages in medium without EGF followed by transplantation into a mouse which generated a tumor, and T4-2 cells were isolated from the serial passage of this tumor; thus T4-2 cells are malignant and tumorigenic [10]. Interestingly, when cultured in three-dimensional (3D) laminin-rich extracellular matrices (lrECM), S1 cells form polarized acinus structures with a central lumen which resemble the terminal milk-secreting alveolar units in normal breasts [6], [11], whereas T4-2 cells form disorganized structures under the same conditions. Signaling molecules such as EGFR, β1-integrin, PI3K, and MAPK are overexpressed in T4-2 cells relative to their levels in S1. Crosstalk between these molecules plays pivotal roles in defining malignant behaviors of T4-2 cells, and downmodulation of them causes phenotypic reversion of T4-2 cells into growth-arrested, normal-looking cells (also called T4R cells later) which form structures resembling S1 acini but often without the lumen [8]. Other molecules, such as TACE or Rap1, have also been shown to be important for reversion of T4-2 cells [12], [13]. NFkappaB was identified as one of the transcriptional regulators involved in disorganization of T4-2 cells [14]. Despite significant efforts to delineate key signaling events responsible for phenotypic reversion of these malignant breast cells, many questions remain. For example, are T4-2 cells reverted by inhibitors of different molecules intrinsically the same? What is involved in the phenotypic reversion of T4-2 cells at the systems level other than a few genes directly related to the signaling molecules mentioned above?

One classical approach to address these questions is to identify genes differentially expressed between different cell states. While this can lead to some information about marginal effects of the genes in a particular stage of cancer progression or reversion, it cannot yield insight into the underlying regulatory mechanisms that govern interaction of genes with one another to carry out complex cellular processes. Instead, we propose a network-based differential analysis, by reverse engineering gene regulatory networks of various conditions of the breast cells to depict a fuller picture of regulatory mechanisms of the cells.

Many methods, as reviewed in [15], [16], have been proposed for reconstructing gene networks using gene expression microarray data. Most of them [17]–[19], however, rely on the statistical assumption that the samples in question were independent and identically distributed (*i.i.d*), and thus they either lead to estimation of a single network by pooling data from all the samples together, or lead to estimation of a network for each cell state independently. Since the breast cells in this study came from non-reverted HMT3522 cells as well as various conditions of the reverted cells, the regulatory mechanisms in different cell states can be significantly different; therefore, pooling data from different cell states together to estimate one single network does not reveal networks in their full depth. On the other hand, reconstructing a network specific to each cell state independently of the other ones can be statistically inaccurate due to a small sample size for each cell state. Recently, time-varying network detection methods have been proposed that allow information sharing across time and can thus recover a sequence of networks even with small sample sizes [20]–[23]. For example, Song *et al.* proposed a time-varying dynamic Bayesian network method to estimate a chain of evolving networks over time [22]. However, these methods estimate networks that evolve as a chain of networks over time, not as a series of networks shared by the tree-shaped phenotypic relationships as shown in Figure 1.

Due to the unique challenges we encountered to reconstruct networks that rewire over the tree-shaped phenotypic relationships, we recently proposed *Treegl* [24], a network reconstruction algorithm that can effectively and jointly recover rewiring regulatory networks present in multiple related cell states. Our approach can not only recover a distinct network for each cell state and reveal sharp differences among networks for different cell states, but also capture and leverage similarities of the networks in the cell states nearby in the phenotypic tree, thereby leading to more accurate estimation of gene interactions in small sample size scenarios. This new angle of estimating networks can reveal information that has not been mined in traditional analysis.

In this paper, we conduct an extensive network analysis of non-reverted HMT3522 cells (normal S1 and malignant T4-2 cells) as well as three different conditions of reverted T4-2 cells using gene expression microarray data obtained from these cells. It is notable that the same set of the gene expression data was first described and used in our previous work published in [24], however, our focus then was to report the novel methodology behind the *Treegl* algorithm, but not a thorough biological analysis of the HMT3522 series of cells from which the gene expression data was generated. In this current work, we focus more on the biological findings discovered by a more comprehensive network analysis of the data using *Treegl* and other bioinformatics tools, and aim to provide better biological insights and understandings of the various breast cell states in the HMT3522 series.

In particular, we estimated the network specific to each cell state using *Treegl*. Our results showed that while the S1-specific network contains predominantly nonmalignant pathways, the T4-specific network contains various cancer-related pathways, both findings consistent with biological evidence [4]. Furthermore, we found that the networks specific to various conditions of the T4-2 reverted cells are enriched with pathways suggestive of compensatory effects. In the T4-2 cells reverted by inhibition of either EGFR or β1-integrin, signaling pathways downstream of EGFR or β1-integrin, mainly via the PI3K-AKT-mTOR axis, are upregulated. Similarly, in the T4-2 cells reverted by either PI3K or MAPKK, we observed upregulation of the pathways both upstream and downstream of PI3K. These results are supported by clinical evidence showing patient resistance to the same anti-breast cancer drugs as we used in the study. Moreover, the compensatory signaling is also observed in the differential network of the T4-2 cells reverted by MMPIs, which involves genes participating in protein catabolic processes. Together, our findings suggest a common resistance mechanism employed by breast cancer cells to antagonize drug effects. Finally, in order to identify potential novel drug targets, we also investigated hubs (i.e., genes with high degrees, see details in Materials and Methods) in the differential networks of the breast cells, and characterized specifically three hubs (NEBL, HBEGF, and PAPD7) whose aberrant expression values are linked with the worst survival outcomes in the breast cancer patients to provide insight into their functional significance on the growth and development of breast cancer cells. Our data suggest that *Treegl* when applied to an effective disease model system, such as the HMT3522 cells, may potentially become an effective tool for elucidating disease mechanism and discovering novel drug targets, and thus help make personalized medicine possible.

## Results

**Figure 2:**
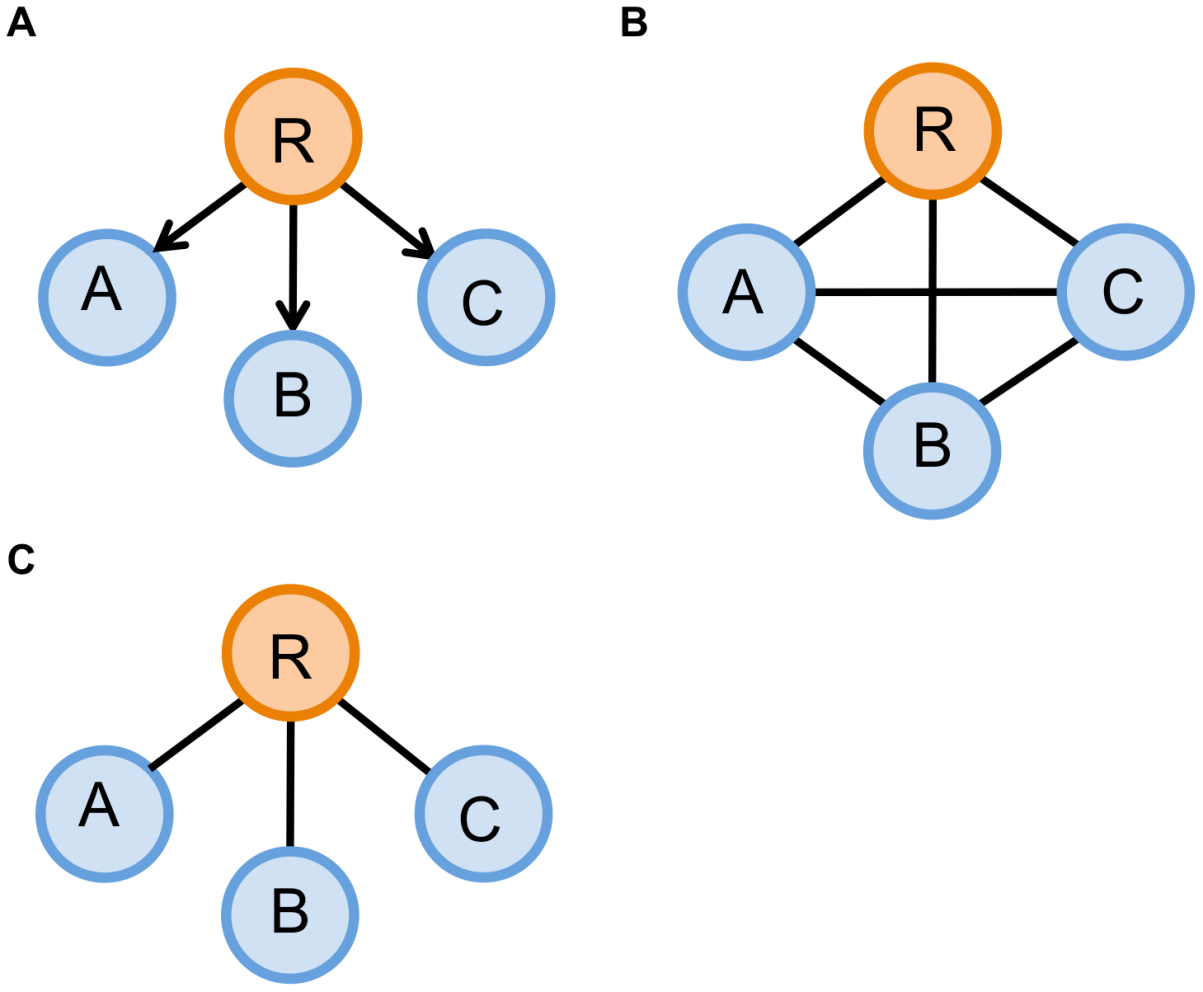
An example demonstrating the difference between a Markov network and a correlation network. (A) A true network, in which ***R*** is the regulator of ***A***, ***B***, and ***C***. (B) A clique graph produced by a correlation network. Since all the genes in (A) are correlated with one another, the correlation network cannot distinguish between indirect and direct relationships and thus connects all the nodes. (C) A correct graph recovered by a Markov network. The Markov network recovers true relationship of the nodes because it uses conditional independence to determine the presence of an edge.

We model a gene network as a Markov network [25], which is a graph 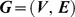 where 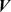 is the set of vertices (genes), and 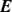 is the set of edges. Genes 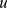 and 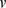 do *not* have an edge between them if and only if they are conditionally independent given the values of all other genes. We contrast this with a correlation network (a common approach for modeling gene networks), in which 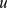 and 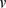 are connected if their marginal pairwise correlation is greater than a certain threshold. Correlation can be effective when analyzing a pair of genes in isolation. However, when studying the dependence between two genes in the *context* of other genes, correlation can confound direct/indirect relationships, thus producing undesirable results (Figure 2).

Specifically, we model the gene network for each cell state 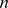 as a *Gaussian* Markov network. Gaussian distributions are special in that the inverse of the covariance matrix, called the precision matrix 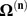, completely encodes the structure of the Markov network. In particular, an edge 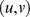 exists in the Markov network if and only if the corresponding precision matrix element is non-zero.

Thus, under our model, the problem of learning the structure of a gene network reduces to estimating 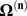. This is straightforward when the number of genes 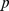 is smaller than that of microarray samples 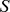: one can compute the sample covariance matrix and simply get its inverse. However, in high dimensional settings, when 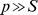 (as is our case), the sample covariance matrix is not invertible, and thus the problem becomes substantially more challenging. A statistically principled solution is the graphical lasso [26], which estimates the neighborhood of gene 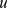 by using regularized regression. The regression coefficients can be interpreted as estimates of the precision matrix elements up to a proportionality constant (see Materials and Methods for more details). After the neighborhood of each gene is estimated independently, the results are combined to form a network.

### Tree-evolving network detection algorithm, *Treegl*

However, our goal is not estimate a single network, but rather a collection of networks, one for each cell state. One simple solution is to estimate the network for each state independently of the others using the graphical lasso. However, this approach can result in poor quality of the networks due to the small sample size per cell state. To overcome this challenge, our recently proposed algorithm, *Treegl* [24], utilizes the following strategy. Similar to the graphical lasso [26], [27], *Treegl* estimates the neighborhood of each gene independently of those of other genes using regularized regression. However, unlike previous methods learning only a single network, *Treegl* simultaneously estimates neighborhoods of a gene in multiple networks each corresponding to a unique state in the phenotypic tree of the breast cells. It is unique in that *Treegl* makes use of a total variation regularizer based on the progression and reversal relationships between pairs of cell states in question to bias the amount of topological differences between networks underlying the related states, and to allow information regarding probabilistic independencies between genes to propagate across all states either directly or indirectly related by phenotypes. Such a strategy can lead to highly statistically confident estimation of a gene Markov network [28], even under small sample size scenarios. In the Materials and Methods section, we will offer details of a novel statistical regularization technique that makes this possible.

From the theoretical standpoint, *Treegl* is an instance of the general varying coefficient varying structure (VCVS) formalism analyzed in [29]. The VCVS model encodes changing structures of gene networks in different cell states as a function of regression coefficients in regularized regression problems. Estimating these regression coefficients, and thus the associated network structures then reduces to solving a convex optimization problem jointly over all cell states. The global optimal solution for such a problem can be found using standard convex solvers.

Moreover, the VCVS formalism allows one to theoretically examine and prove the statistical conditions under which changes in structures can be correctly estimated even in the high dimensional setting when 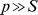. This distinguishes our approach from other methods [17], [30] that are highly non-convex and therefore rely on local search heuristics that only find local optima. These existing methods also do not offer sound statistical machinery for addressing difficult conditions such as nonstationarity (e.g., time-evolving) and high-dimensionality under small sample size as we encountered in our study.

Having a theoretical framework allows us to trade-off model expressivity and learning complexity in a principled manner. For example, if we allow a complex and arbitrary network model (i.e., a dense network), then there would be no guarantees on the quality of the recovered network structure in small-sample size scenarios. Instead, by enforcing a restricted model (i.e., a sparse network), its likelihood function is by definition convex and an optimal solution may be found. Thus, the quality of the resulting solution can be theoretically characterized, and it can be determined under which conditions the correct underlying parameters (network structure in this case) are discovered. Fortunately, sparsity is also biologically justifiable. For example, it is common to find a transcription factor regulating a limited number of genes under specific conditions [31].

### Simulation results

We first evaluate *Treegl*'s performance on simulated microarray data. In order to find out how effectively *Treegl* can detect change points of multiple networks while sharing information among related cell states at the same time, we design the simulated networks as illustrated in Figure S1. In particular, for each experiment, an artificial collection of 70 networks related by a tree-shaped lineage are generated, in which a sequence of 10 identical networks is connected to a network of different topology via a change/branching point (see details in Materials and Methods). Then, a small number of samples are generated from each of the networks. It is important to note that *Treegl* does not know a priori which of the networks are identical and which are different and thus has to discover this based on the samples.

**Figure 3:**
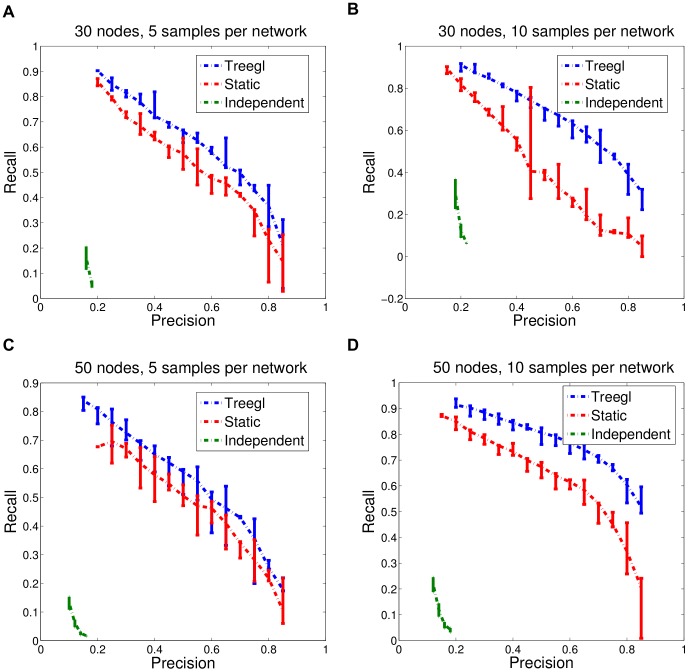
Simulation results comparing the performance of *Treegl* to a method estimating a single static network and a method estimating each network independently. In all cases, 70 networks were generated that are related by a tree lineage (See Materials and Methods for details). (A) Each network has 30 nodes and 5 samples. (B) Each network has 30 nodes and 10 samples. (C) Each network has 50 nodes and 5 samples. (D) Each network has 50 nodes and 10 samples. In all cases, *Treegl* (shown in blue) performs favorably to the method estimating a single static network (red) or the method estimating each network independently (green).

In order to evaluate how well *Treegl* can recover the underlying network structures for the samples in the simulation data, we compare *Treegl* with the static method estimating a single network and the method estimating each network independently by plotting the precision-recall curves which show the recall for different values of precision based on the network estimated by the three methods. As illustrated in Figures 3 & S2, *Treegl* performs favorably to the other two methods. It should also be noted that compared to the static method which produces only one network, *Treegl* can produce different networks related by the tree lineage. The independent method also produces different networks but it performs poorly compared to *Treegl*.

### A phenotypic tree representation of the HMT3522 series of the breast cells

In order to reverse engineer gene networks of the breast cells, we first used a phenotypic tree to represent the relationships of the cells (Figure 1). Due to the small sample size of the microarray data and imbalance of the sample abundance for different cell states (see Materials and Methods for details) — both of the problems pose significant challenges to network reconstruction — we used what is known of the interrelatedness of signaling pathways affecting phenotypic reversion to pool data derived from various samples in order to increase the power of the network inference. In particular, since EGFR and β1-integrin are cross-modulated in the HMT3522 cells [8], we assumed that the gene networks in the T4-2 cells reverted by inhibiting either of the molecules share reasonable similarity, and hence we grouped data from these reverted cells together to form the EGFR/ITGB1-T4R group. Likewise, we grouped together data from T4-2 cells reverted by either a PI3K inhibitor, a MAPK inhibitor, or a dominant-negative Rap1 to form the PI3K/MAPKK-T4R group, because PI3K and MAPK are also cross-modulated in the breast cells and Rap1 signals through PI3K. A tree diagram illustrating the relationships of the cells are shown in Figure 1. Based on these relationships, we reverse engineered gene networks for the HMT3522 cells using *Treegl*.

It is important to point out that it would be nearly statistically impossible to reconstruct a cell-state-specific gene network using existing methodology based on three microarray samples per group as in the dataset we used here. Note that we will also refer to different groups of the cells in the phenotypic tree in Figure 1 as different cell conditions or states.

### The reconstructed gene networks for the HMT3522 cells

The reconstructed networks for non-reverted and various conditions of the reverted HMT3522 cells are illustrated in Figure S3. They share many topological similarities as well as differences. About 60% of the network edges are common to all cell conditions represented in the phenotypic tree diagram, consistent with underlying biological similarities shared between them. In the following, we concentrate on only the edges specific to each cell state, which we call the *differential network* for each cell state.

#### S1 differential network

**Figure 4:**
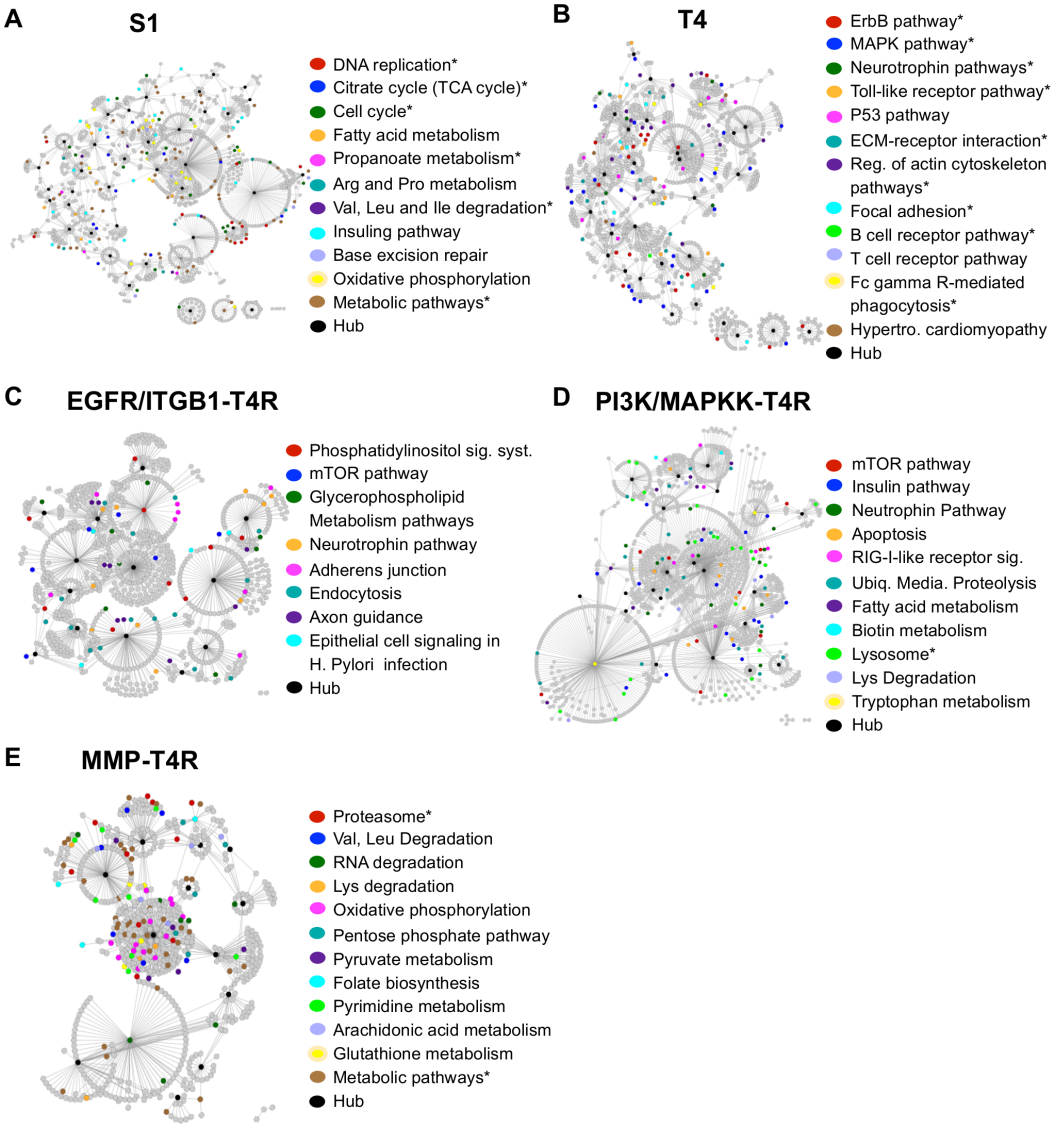
Illustration of selected enriched pathways in the differential network of each breast cell state. (A) S1; (B) T4; (C) the EGFR/ITGB1-T4R group; (D) the PI3K/MAPKK-T4R group; and (E) the MMP-T4R group. In each plot, the differential network for the corresponding cell state is shown. Nodes represent genes; edges represent interaction of the genes. Selected pathways enriched in the network (unadjusted p-values<0.05) are color-coded and shown on the right; genes participating in the selected pathways are also colored based on the color code for the corresponding pathway. An asterisk indicates a pathway that is significantly enriched in the corresponding differential network. The pathway names are shortened to save space. See Table S1 for detailed information about the enriched pathways in each cell state.

Our pathway and GO analysis showed that genes in the S1 differential network are significantly enriched with those involved in normal cellular processes, such as cell cycle, TCA cycle, and cellular respiration (Figure 4A, Tables S1A & S2A). Notably, genes involved in tube lumen formation are enriched only in S1 cells (Table S2A) while absent in the other cell states, consistent with the observation that a central lumen is always present in the acinus structure formed by S1 cells but often absent from spheres formed by reverted T4-2 cells. Furthermore, our disease relevance analysis did not find association of the genes in the S1 differential network with any disease (Table S3A). Together, these results agree with the biological fact that S1 cells are nonmalignant.

#### T4-2 differential network

**Figure 5:**
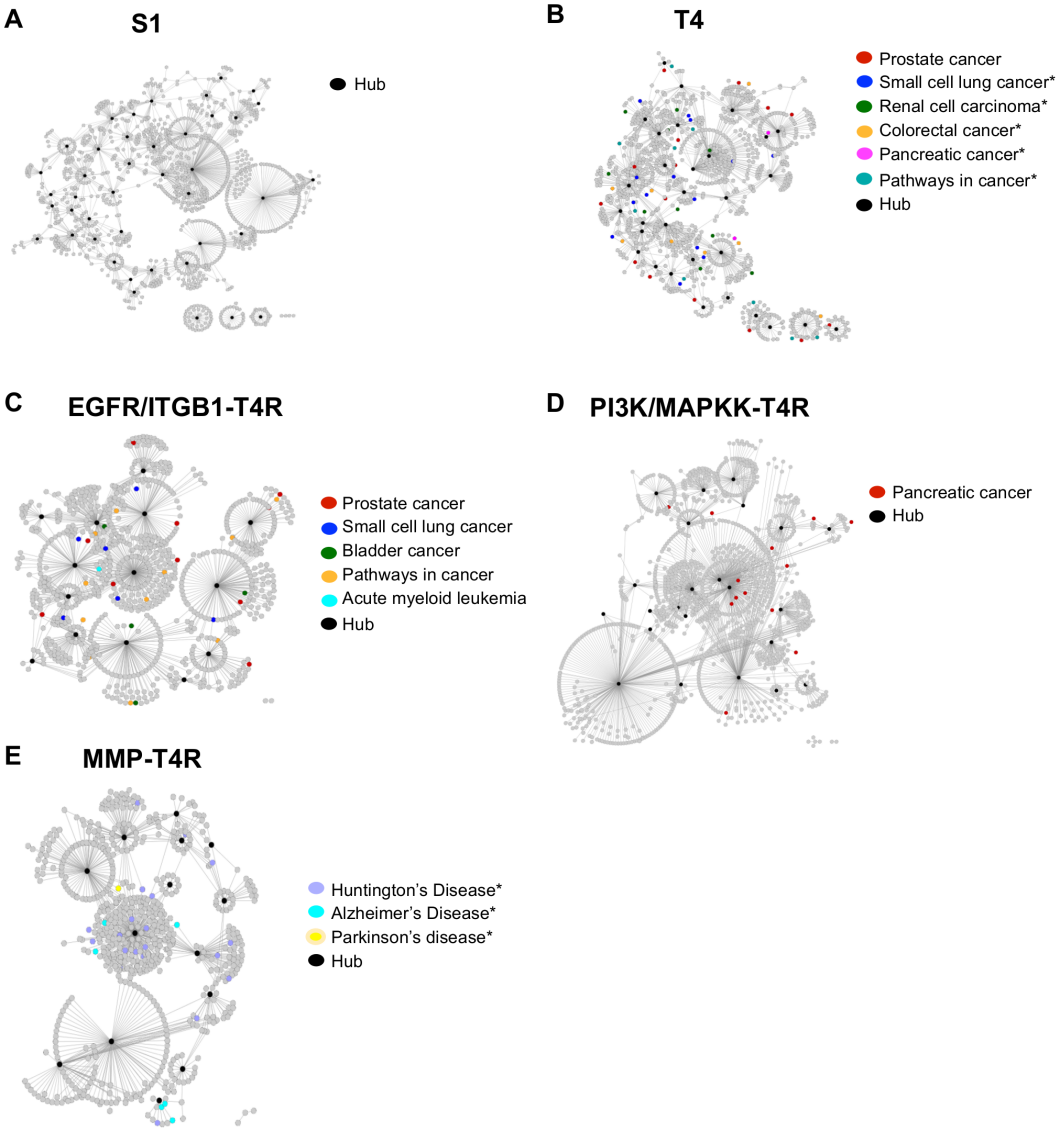
Diseases associated with the genes in the differential network of each breast cell state. (A) S1; (B) T4; (C) the EGFR/ITGB1-T4R group; (D) the PI3K/MAPKK-T4R group; and (E) the MMP-T4R group. In each plot, the differential network for the corresponding cell state is shown. Nodes represent genes; edges represent interaction of the genes. Diseases associated with the genes in the network (unadjusted p-values<0.05) are color-coded and shown on the right; genes associated with a certain disease are also colored based on the color code for the corresponding disease. An asterisk indicates a disease that is significantly enriched in the corresponding differential network.

The T4-2 differential network, on the other hand, is significantly enriched with genes involved in a number of pathways important for tumor growth and progression, such as ErbB and MAPK signaling pathways, ECM-receptor interaction, and regulation of actin cytoskeleton pathways (Figure 4B, Table S1B). Moreover, disease relevance analysis showed that genes in the T4-2 differential network are associated with various cancers, such as small cell lung cancer, renal cell carcinoma, colorectal cancer, among others (Figure 5B, Table S3B), and that genes involved in the pathways such as ErbB and MAPK signaling pathways, focal adhesion, and ECM-receptor interaction (Table S1B) have significant association with pathways in cancer (FDR adjusted p-values<0.1). These data are supported by biological evidence showing that ErbB and MAPK signaling pathways, microenvironment, and integrity of tissue architecture play significant roles in the malignant T4-2 cells [4], [6]–[8]. Together, these functional results suggest that *Treegl* can indeed reveal biological characteristic that is specific to the states in the HMT3522 cells.

#### Differential networks of the three conditions of the reverted T4-2 cells

In order to find out whether T4-2 cells reverted by various agents are merely phenotypically or intrinsically normal, and also whether these different conditions of the reverted cells are identical to one another at the systems level, we examined the differential networks specific to each condition of the reverted T4-2 cells.

#### EGFR/ITGB1-T4R differential network

Our pathway analysis suggests that there are no pathways significantly enriched in the differential network for this group of the cells (Table S1C). However, a close examination of the enriched pathways (unadjusted p values<0.05) revealed that genes involved in the pathways downstream of EGFR, such as phosphatidylinositol and mTOR pathways are enriched (Figure 4C, Tables S1C & S2C). Since PI3K is a component of the phosphatidylinositol pathway, the enrichment of the pathway suggests upmodulation of PI3K in these cells (Figure S4). This is confirmed by the results showing PIK3R2 (encoding a regulatory subunit of PI3K) and AKT1, but not mTOR itself, are among the genes upmodulated in the enriched mTOR pathway (Figure S5). Together, these results suggest PI3K-AKT-mTOR signaling is activated in the EGFR/ITGB1-T4R cells. Further, genes in the EGFR/ITGB1-T4R differential network are connected with various cancers, including prostate, and small cell lung cancer (Figure 5C, Table S3C), suggesting that despite phenotypic reversion to normal, some malignant genes remain active in the EGFR/ITGB1-T4R cells.

Interestingly, activation of PI3K-AKT-mTOR signaling in our data agrees well with clinical evidence showing that some breast cancer patients develop drug resistance after being treated with EGFR inhibitors, and that compensatory signaling via the PI3K-AKT-mTOR pathway has been implicated in such resistance [32]–[34]. Furthermore, our data suggest that mTOR signaling is not mediated directly by mTOR, but rather by other genes in the mTOR pathway, because mTOR per se is not involved in the differential network of this condition of the reverted cells; multiple other genes in the pathway, however, are upregulated. These results also agree with previous evidence in the literature; despite a close link between mTOR signaling and cancer, overexpression of mTOR has not been reported in human tumors; instead, signaling events related to mTOR occur frequently [35]. Our results may thus identify genes directly involved in such drug resistance.

#### PI3K/MAPKK-T4R differential network

As expected, the enrichment of the PI3K-related pathways is absent in the differential network of the PI3K/MAPKK-T4R group of the reverted cells due to the blockage of intracellular signaling (Figure 4D). Our data, however, show that pathways both upstream (insulin pathway) and downstream (mTOR pathway) of PI3K are enriched (unadjusted p-values<0.05, Table S1D, Figures S6 & S5), suggesting activation of compensatory signaling due to the loss of PI3K signaling in the cells. Moreover, genes in the differential network, particularly those involved in the pathways such as mTOR, insulin, and apoptotic signaling, showed association with cancer (Figure 5D, Table S3D). Even though the p-values of the pathways are not significant after the FDR adjustment, these results agree with recent clinical findings that some breast cancer patients exhibit resistance to treatment by PI3K inhibitors, and that mTOR and insulin growth factor (IGF) signaling pathways have been implicated for such resistance [34]. However, no genes in these pathways have been identified for this resistance yet. A close connection between insulin and IGF pathways can be seen in Figure S5.

#### MMP-T4R differential network

Unlike the differential networks in the other phenotypic reversion conditions mentioned above, the MMP-T4R differential network has far fewer edges, suggesting that fewer biological processes take place in the MMP-T4R group of the reverted cells. Functional pathway and GO analysis revealed that genes involved in protein catabolic processes and transfer, such as ubiquitin-dependent and proteasomal protein catabolic processes and receptor-mediated endocytosis, are significantly enriched (FDR p-values<0.05, Figure 4E, Tables S1E & S2E). Interestingly, MMPs are known to play key roles in protein catabolic processes, these results, therefore, suggest that when MMPs are inhibited in T4-2 cells, genes participating in protein catabolic processes and transfer are upregulated as compensatory pathways to make up for the loss of the MMP function. Since protein catabolic processes have been implicated in tumor development [36], our data suggest the MMP-T4R cells still possess tumor-developing potentials, consistent with clinical data showing MMP inhibitors (MMPIs) delivered disappointing results as anticancer treatments in clinical trials [37], [38]. Furthermore, disease relevance analysis showed that some pathways, such as oxidative phosphorylation and metabolic pathways, in the MMP-T4R differential network are significantly associated with diseases, such as Alzheimer's and Parkinson's diseases (Figure 5E, Table S3E). This suggests that despite being not as malignant as T4-2 cells, the MMP-T4R group of the reverted cells are not intrinsically normal.

### Survival analysis of hubs

In order to validate our network reconstruction results, we used an external microarray dataset. Since previous evidence suggests that there is a high correlation between the degrees and essentiality of genes in yeast networks, we hypothesize that i) if a gene is indeed involved in the networks of the breast cells, its abnormal expression would have higher impact on the survival of breast cancer patients than those genes which are not in the networks; and ii) if a gene is a hub (with a high degree) in the differential networks of breast cells, its abnormal expression would have higher impact on the survival of breast cancer patients than those with low degrees. We therefore investigated the effect of the aberrant expression values of the hubs in the differential networks of the HMT3522 cells on the survival of human breast cancer patients. The external dataset we used is a gene expression microarray dataset obtained from 295 primary human breast tumors [39], employed previously to identify gene expression signatures which may be predictive of patient clinical outcomes. The same dataset was also used previously [12] to demonstrate the impact of abnormal expression of TACE, TGFA, and AREG, which were shown to play important roles in the HMT3522 series of the breast cancer cells grown in the 3D culture, on the survival of the same cohort of the breast cancer patients.

In order to define hubs, we examined the distribution of genes with varying degrees in the differential networks. Figure S7 shows that while a majority of the genes have degrees of 1–3, much fewer genes have a degree greater than 5, and therefore, we designated hubs to be genes with degree greater than 5 in the differential networks. Note that the same criterion was also used to define hubs in [40].

Indeed, we found that 18% of the genes in the networks of the five breast cell states affect patient survival significantly, whereas that only 6% of the genes which are not in the breast cell networks but are present in the external dataset affect patient survival significantly. Our results also showed that 22% of the hubs in the differential networks of the breast cells affect patient survival significantly. GO analysis revealed that these significant hubs are enriched with genes involved in regulation of cell migration, mobility, growth, and proliferation (see Table S4 for a list of the hubs), and all of these biological activities are known to be essential for cancer cell development and progression. Similarly, 23% of the genes with degree >10 and also with degree >20 affect patient survival significantly. However, for genes with degrees equal to one to five in the differential networks, the percentage of them affecting patient survival drops to 17%. Together, these results indicate that i) genes in the breast cell networks indeed have higher tendency of influencing patient survival significantly than those not in the networks, and also that ii) hubs in the differential networks are more likely to affect patient survival significantly than those with low degrees, suggesting the structures of the reconstructed networks are valid.

### Neighborhood analysis of the hubs significantly affecting patient survival

**Figure 6:**
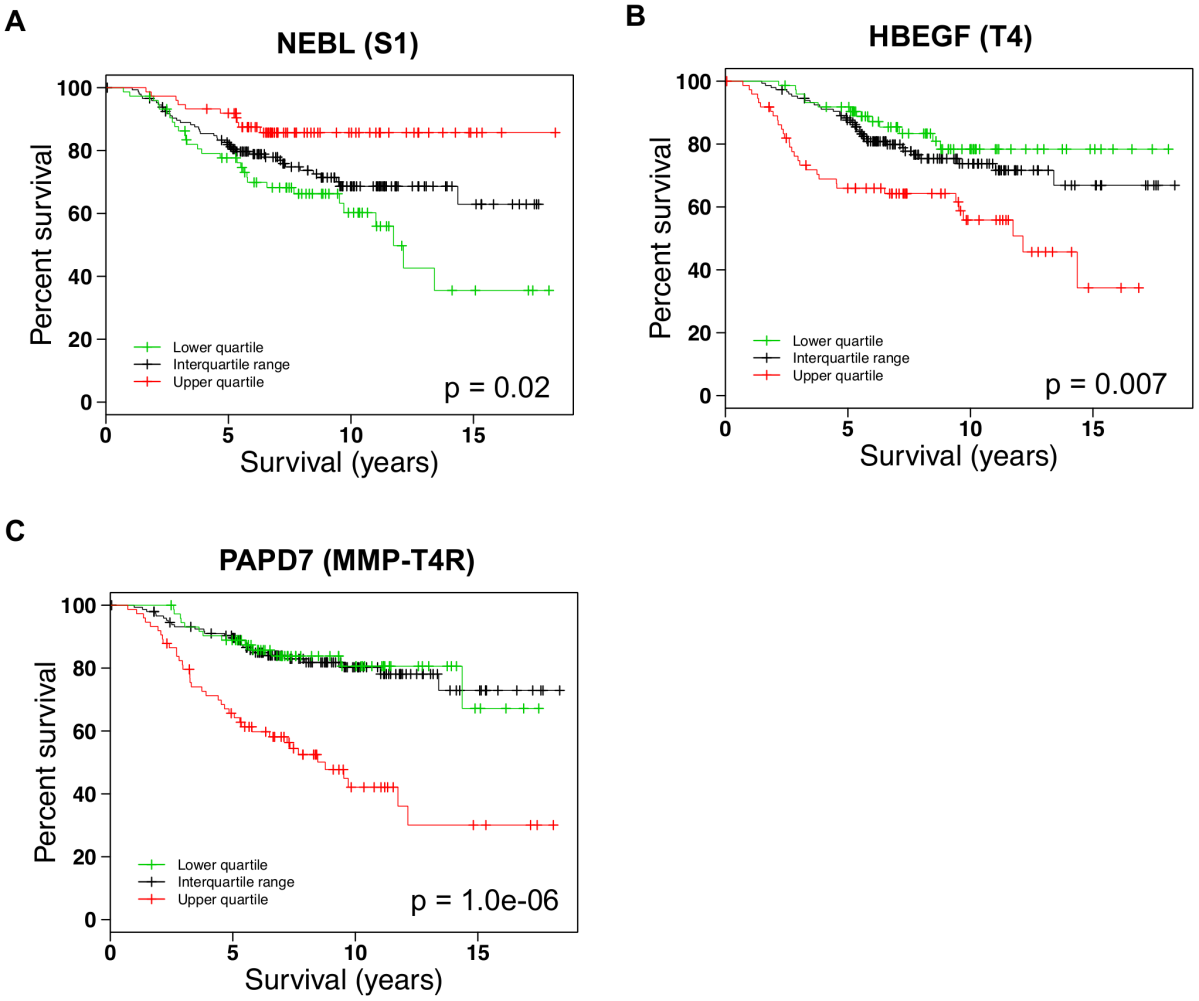
Kaplan–Meier curves estimating the association of different expression values of three hubs in the differential networks of the breast cell states with survival of breast cancer patients. (A) NEBL in the S1 differential network; (B) HBEGF in the T4-2 differential network; (C) PAPD7 in the MMP-T4R differential network.

In order to identify potential novel drug targets, we examined three hubs, NEBL, HBEGF, and PAPD7, whose extreme (i.e., either lower or higher) expression values are correlated with the lowest 15-year patient survival rates (35%, 30% and 34%, respectively) and also with low 10-year survival rates (60%, 42% and 56%, respectively) in the examined dataset (Figure 6). Previous evidence has shown that abnormal expression of TACE, TGFA, and AREG are associated with 62%, 61% and 54% of the 10-year survival rates respectively, and associated with 57%, 50% and 54% of the 15-year survival rates respectively, in the same cohort of the patients [12]. Our results, therefore, suggest that NEBL, HBEGF, and PAPD7, similar to TACE, TGFA, and AREG, also play important roles in breast cells.

Since little is known about NEBL, HBEGF, and PAPD7, we examined their neighbors in the corresponding differential networks (which we call neighborhood analysis) to shed light on their functions in breast cancer.

**Figure 7:**
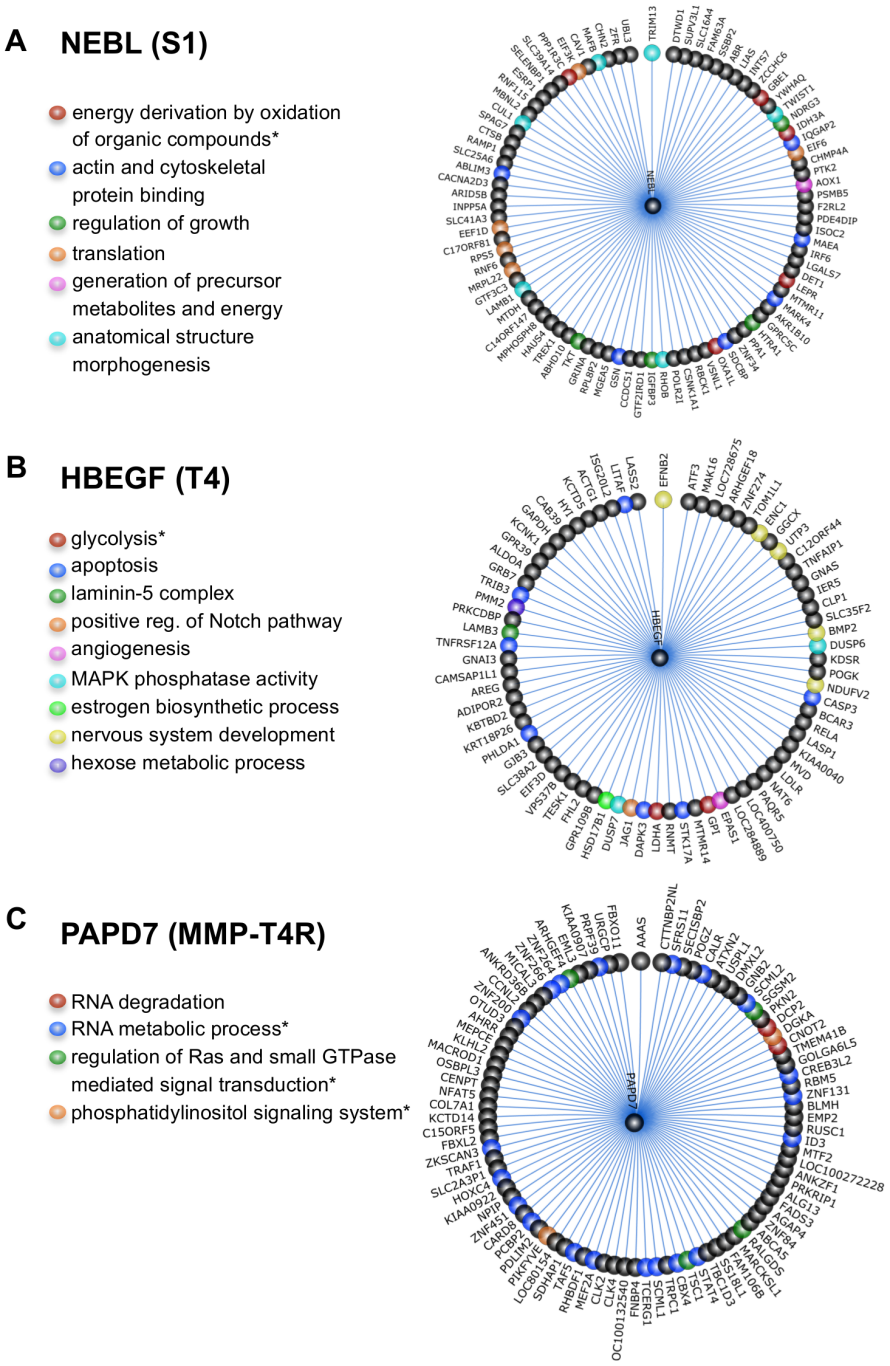
Selected pathways or GO groups in the neighborhood of the three hubs in the differential networks of the breast cell states. (A) NEBL in the S1 differential network; (B) HBEGF in the T4-2 differential network; and (C) PAPD7 in the MMP-T4R differential network. An asterisk indicates a pathway or a GO group that is significantly enriched in the corresponding differential network.

Figure 7A shows the NEBL subnetwork in the S1 differential network. NEBL encodes a member of the nebulin family of proteins, which bind actin and are components of focal adhesion complex. Our data showed that decreased expression of NEBL is associated with 36% of 15-year survival rate for breast cancer, suggesting a protective role of this protein when overexpressed. Genes interacting with NEBL in the NEBL subnetwork are mainly involved in energy production by oxidation of organic compounds, actin and cytoskeletal protein binding, regulation of growth, and anatomical structure morphogenesis, all of which are consistent with the biological evidence suggesting involvement of nebulin in migratory cells [41].

Figure 7B shows the HBEGF subnetwork in the T4-2 differential network. HBEGF encodes a heparin-binding EGF-like growth factor, which is an EGFR ligand [42]. We found that higher expression of HBEGF is correlated with 34% of 15-year survival rate (Figure 6B), and that the neighbors of HBEGF in the HBEGF subnetwork are involved in diverse biological processes and functions, such as glycolysis, apoptosis, participating in laminin-5 complex, notch signaling pathway, and angiogenesis, all of which are in line with previous findings showing the high expression level of HBEGF is positively related to the aggressiveness of the breast tumors [43] and that HBEGF plays key roles in tumorigenicity and invasiveness of ovarian cancer [44].

Finally, we examined the PAPD7 subnetwork in the MMP-T4R differential network (Figure 7C). PAPD7 encodes DNA polymerase sigma. Our data indicated that overexpression of PAPD7 is associated with 30% of 15-year survival rate and 8.5 years of median survival time (Figure 6C), both of which are the worst patient outcomes correlated with all the hubs in the differential networks, suggesting that PAPD7 plays significant roles in breast cancer cells. Previous evidence showed that a homolog of PAPD7 in *Saccharomyces cerevisiae* Trf4 plays a key role in RNA quality control by degrading aberrant or unwanted RNAs in the nucleus [45]. Interestingly, our functional analysis revealed that genes interacting with PAPD7 in the MMP-T4R differential network are significantly enriched with those involved in RNA degradation and metabolic process, as well as regulation of Ras and small GTPase mediated signal transduction, and phosphatidylinositol signaling system (Figure 7C), which implicates that similar to Trf4, PAPD7 also participates in crucial functions such as RNA quality control in human cells.

Taken together, these findings suggest that the three hubs, NEBL, HBEGF, and PAPD7, in the differential networks play important roles in growth and development of breast cancer cells, and may thus become potential novel therapeutic targets. More important, these results also suggest that our reconstructed networks can not only reveal genes which have high impact on patient survival in specific cell conditions, but also can provide insight into their functions by neighborhood analysis, and thus facilitate personalized drug target discovery and identification, and help make personalized breast cancer therapy possible.

## Discussion

The problem of estimating rewiring networks simultaneously from multiple cell states in the phenotypic tree, as solved by *Treegl*, is fundamentally different from either estimating a single “average” network from the samples pooled from all states and subsequently “trace-out” active subnetworks corresponding to each state [1], or estimating multiple networks independently. The latter strategies are common practices in the system biology community, which either directly or indirectly assume the network in question is static, and samples of the nodal states in the phenotypic tree are *i.i.d.* across (when pooled) or within cell states. In reality, such an assumption is biologically invalid as well as statistically unsubstantiated. The *Treegl* algorithm elegantly couples all the inference problems pertained to each network in the tree of multiple conditions, and achieves a globally optimal and statistically well behaving solution based on a principled VCVS model and a convex optimization formulation.

In our analysis of the HMT3522 breast cancer cell lines, we reverse engineered 5 different gene networks specific to each cell state represented in the phenotypic tree. The S1 differential network contains genes predominantly involved in normal cellular activities, while the T4-2 differential network is enriched with pathways playing active roles in cancers. Interestingly, compensatory signaling appears to be a recurring theme of the T4-2 cells phenotypically reverted by different agents. In the T4-2 cells reverted by inhibition of either EGFR or β1-integrin (i.e., the EGFR/ITGB1-T4R group), despite the absence of the ErbB pathway, signaling events downstream of EGFR or β1-integrin, mainly via the PI3K-AKT-mTOR axis, seem to be upregulated. These results are supported by clinical evidence showing that some breast cancer patients exhibit drug resistance after being treated with EGFR inhibitors. Similarly, in the PI3K/MAPKK-T4R cells, their differential network is enriched with genes closely connected to PI3K, suggesting they are upmodulated to make up for the loss of PI3K signaling, also agreeing with clinical findings showing patient resistance to PI3K inhibitors. Likewise, the compensatory effect is observed in the differential network of the T4-2 cells reverted by MMPIs, which involves genes participating in protein catabolic processes presumably to make up for the loss of the MMP function. The effect of MMPIs for treating breast cancer patients was disappointing in clinical trials, but no conclusive evidence for ineffectiveness has been put forward [38]. Our results suggest that the failure of treating breast cancer patients by MMPIs involves upmodulation of the catabolic processes in the treated patients due to compensatory effect. Together, these results suggest despite phenotypic similarities, T4-2 cells reverted by various drugs are intrinsically different from one another; similar compensatory mechanisms, however, appear to be utilized by the T4-2 cells to antagonize effects of the different drugs.

In order to compare our network-based approach with traditional statistical test-based approach, we also analyzed the gene expression data using ANOVA, and identified 1432 genes significantly differentially expressed (FDR p-value<0.05) across different cell states; then we used pairwise t-tests to further identify significant differences between cell states. We found that due to small sample size problems, these traditional approaches are too stringent to reveal interesting signals. For example, we examined the genes differentially expressed between the T4-2 cells reverted by MMP inhibitors (MMP-T4R) and other cell states, in particularly between MMP-T4R and S1, as well as between MMP-T4R and T4. Our results show that there are 473 genes significantly differentially expressed in MMP-T4R, comparing to S1, and the only two GO functional groups significantly enriched (FDR p-value<0.05) among these genes are “mitotic cell cycle” and “sterol biosynthesis process.” Comparing to T4, there are 375 genes differentially expressed in MMP-T4R, and there are no GO groups significantly enriched among these genes.

Moreover, we examined genes in the differential network of MMP-T4R which are involved in some of the significantly enriched GO groups, e.g., “proteasome complex” and “cellular catabolic process”, both of which suggest compensatory signaling in the MMP-T4R cells. We found that among 12 genes in the differential network of MMP-T4R (“PSME3, PSMA4, PSMB8, PSMD10, PSMA3, PSMB9, PSME2, PSMD7, PSMA6, PSMC2, PSMA2, PSMD6”) which are involved in “proteasome complex”, only two of them (PSMA3, PSMB9) significantly differ between MMP-T4R and S1 as identified by ANOVA, and two (PSMC2, PSMB9) significantly differ between MMP-T4R and T4. Likewise, among 33 genes in the differential network of MMP-T4R which are involved in “cellular catabolic process”, only 5 genes (“PSMB9, ANAPC5, USP18, IDH1, PSMA3”) significantly differ between MMP-T4R and S1 as identified by ANOVA, and 3 genes (“PSMB9, USP18, IDH1”) differ between MMP-T4R and T4.

Furthermore, we looked into the 22 hubs in the differential networks which significantly affect patient survival, and found that only 8 (36%) of them are differentially expressed across 5 cell states as identified by ANOVA and a majority (64%) of them are not differentially expressed. Similarly, among 99 hubs in the differential networks, 43% are differentially expressed, while 57% are not. These results suggest that under small-sample-size scenarios, traditional statistical tests are too stringent to capture interesting signals, while our network-based differential analysis can leverage on similarities among different samples while revealing key differences which set them apart.

In order to identify potential novel drug targets, we also investigated hubs in the breast cells whose aberrant expression values are significantly associated with survival outcomes of breast cancer patients. We found that genes in the networks of the breast cells have 2 times higher tendency than those not in the networks to affect patient survival in the cohort we studied. Also, hubs in the breast networks appear more likely to influence patient survival than genes with low degrees. Indeed, the proportion of the hubs with high degrees which are significant survival genes (22% for hubs with degree >5, and 23% for hubs with degree >10 and also for those with degree >20) is not much higher than that (17%) of the genes with low degrees. The reasons for this can be explained as follows.

When previous evidence suggests that in yeast networks, a gene with a higher degree is more likely to be an essential gene [46], [47], an essential gene is defined as “the cell is unviable when the gene is knocked off” [47]. However, it is difficult to know/determine which genes are essential in humans. Nevertheless, in light of the definition of ‘essentiality’ in yeast, we think it is plausible to believe that the actual percentage of the hubs (with degree >5) in the differential networks of the breast cells, which can affect patients significantly, is 22%+x%, rather than 22%, and the reasons why we cannot see the phenotypic effect of the x% of the hubs on patient survival may include: i) these hubs are so essential to humans that any abnormality would lead to death, even before breast tumors were formed or diagnosed; and/or ii) there are some redundant genes which can make up for the loss/gain of functions of these essential hubs.

Despite the fact that our results suggest that the genes in the breast cell networks are more likely to affect patient survival than those which are not, and also that hubs in the differential networks tend to affect patient survival more than genes with low degrees, our data show that the distributions of the patient survival rates (5-year, 10-year or 15-years) associated with these different groups of genes are not significantly different, suggesting that the patient survival rates are not only affected by degrees of genes in the breast cell networks, but also affected by the functionalities of the genes.

We have also characterized the three hubs in the cell-state-specific differential networks whose aberrant expression values are linked with the worst survival outcomes in the breast cancer patients: NEBL in S1 cells, HBEGF in T4-2 cells, and PAPD7 in the MMP-T4R group of the reverted cells. Our results are not only in line with existing information known about these genes, but also provide insight into their functional significance on the growth and development of breast cancer cells. These hubs are promising to serve as potential drug targets for personalized breast cancer therapy.

The major challenge of this work is the small sample size of the microarray data we have used for the network inference. The data was from 15 microarrays in total, and the T4-2 cells reverted by different agents had to be pooled together in order to increase the power of the network inference. Even though the sample grouping strategy is biologically justifiable (see details in the Results section), our abilities to find differences between T4-2 cells reverted by different agents are limited due to mixed samples in the EGFR/ITGB1-T4R and PI3K/MAPKK-T4R groups of the reversion cells. For example, it is difficult to dissect which specific pathways are abnormally regulated (compared to S1 cells) in which reversion cell state: T4-2 reverted by EGFR inhibitors or by ITGB1 inhibitors. Likewise, it is also difficult to reveal differences in the T4-2 cells reverted by different agents in the PI3K/MAPKK-T4R group. Moreover, mixed samples can reduce power to detect interesting signals in the data. Despite suggesting compensatory events in the reversion cells, the enriched pathways in the EGFR/ITGB1-T4R and the PI3K/MAPKK-T4R cells are not significant (unadjusted p-values<0.05, but FDR p-values>0.1). However, since our data agree well with clinical evidence, they may facilitate clinicians to identify specific molecules which lead to resistance in the drug-treated breast cancer patients. In order to overcome the limitations of the mixed samples, we also focus on finding similarities of the different T4-2 reversion cells. Our results show that we were able to discover a significant amount of information that agrees with the facts and evidence previously known in the literature. Moreover, we were also able to delineate a mechanistic framework at the systems level that can facilitate further elucidation of the mechanisms underlying different states of the breast cells in the progression and reversion model. Experimental validations are nevertheless needed to further verify our findings.

In summary, this work demonstrates our recently developed *Treegl* algorithm can not only provide a holistic view (i.e., the so-called “pan-cell-state” view that echoes the emerging “pan-cancer” or “pan-disease” approach nowadays to biomedical analysis) of the progression and reversion model of the breast cells worthy of further exploration, but also allows us to gain a deeper and systems-level understanding about the behaviors of nonmalignant and malignant breast cells, which may help novel drug target discovery and make personalized breast cancer therapy possible.

## Materials and Methods

### Cell culture and microarray hybridization

HMT3522 S1 and T4-2 cells were grown in 3D lrECM as previously described [6], [48]. The T4-2 cells were reverted using each of the following reverting agents as described previously: an EGFR inhibitor Tyrphostin AG 1478 and a human EGFR-blocking monoclonal antibody mAb225 [7], a β1-integrin inhibitor AIIB2 [6], a MAPK inhibitor PD98059 [7], a PI3K inhibitor LY294002 [8], dominant-negative Rap1 [13]; an MMP inhibitor GM6001 [49], and a broad-range inhibitor of MMPs and ADAMs, TNF protease inhibitor–2 (TAPI-2) [12].

S1, T4-2 and reverted T4-2 cells were isolated from 3D cultures with PBS/EDTA as previously described [50]. Total cellular RNA was extracted using RNeasy Mini Kit with on column DNase digestion (Qiagen). RNA was quantified by measuring optical density at A260 and quality was verified by agarose gel electrophoresis. Purified total cellular RNA was biotin labeled and hybridized to the Affymetrix GeneChip human genome HG-U133A arrays as previously described [51].

### Gene expression microarray data and the sample grouping strategy

Gene expression microarray data was obtained from 15 total RNA samples prepared from the HMT3522 breast cells grown in 3D lrECM and treated with various reverting agents or vehicle controls as mentioned above. Unfortunately, T4-2 cells reverted by some agents have only one sample per each reversion cell state. Even though our method, *Treegl*, is designed for small sample size scenarios, having only one sample per state is not enough for network inference — as it is known that it takes at least two samples to measure even a simple quantity like correlation. Thus, in order to increase the power of the network inference, we grouped the arrays into the following five categories with each having 3 samples: (i) S1 cells (3 arrays); (ii) T4-2 cells (3 arrays); (iii) the EGFR/ITGB1-T4R group, which contains two arrays of the T4-2 cells reverted by the EGFR inhibitor Tyrphostin AG 1478 and the human EGFR-blocking monoclonal antibody mAb225, respectively, and one array of the T4-2 cells reverted by a β1-integrin inhibitor AIIB2; vi) the PI3K/MAPKK-T4R group, which contains one array of the T4-2 cells reverted by a MAPK inhibitor PD98059, one array of the T4-2 cells reverted by a PI3K inhibitor LY294002, and one array of the T4-2 cells reverted by dominant-negative Rap1; and (v) the MMP-T4R group, which contains two arrays of the T4-2 cells reverted by an MMP inhibitor GM6001, and one array of the T4-2 cells reverted by a broad-range inhibitor of MMPs and ADAMs, TAPI-2. The biological justification on this grouping strategy is provided in the Results section.

In order to identify networks specific to each state of the breast cells, we utilized a phenotypic tree model to represent the relationships of different states of the breast cells (Figure 1). In particular, since the HMT3522 series were originated from S1 cells, we positioned S1 cells as the root of the phenotypic tree. Then we made S1 cells the parent of T4-2 cells, since T4-2 cells were derived from S1 cells. Finally, we made T4-2 cells the parent of the three conditions of the T4-2 cells reverted by various agents (the EGFR/ITGB1-T4R group, the PI3K/MAPKK-T4R group, and the MMP-T4R group).

### Microarray data preprocessing

Raw gene expression data was preprocessed using the following procedure. The data from the *perfect match* (PM) probes on the Affymetrix arrays was first log2-transformed, and normalized using the *CyclicLoess* normalization method to minimize unwanted noise in the data [52]. We did not use the difference between the values from the PM probes and those from the mismatch (MM) probes (i.e., PM – MM) to represent values of the probes for each gene, because it has been shown that the MM values can pick up both non-specific and specific signal of the probes, and thus PM-MM values may attenuate real signal values from the PM probes [53]. The normalized PM values were then summarized into gene expression values using the median polish technique [54]. For some transcripts, multiple probes on an array target the same transcript; the values of the probes were combined by taking the median of the values to represent the expression level of the corresponding transcript. There are 12,977 unique genes on the arrays. The complete microarray dataset is available at the Gene Expression Omnibus (GEO) database (http://www.ncbi.nlm.nih.gov/geo - GSE42125).

To reduce biological noise in the data, we removed genes whose expression values showed low variability across different groups of the breast cells. In particular, for each gene, we calculated its median expression values in five different groups of the breast cell states. If the fold change value of a gene between any of the two groups was larger than 1.3, we included the gene for the downstream analysis. The reasons why we used the fold change of 1.3 as the threshold value to filter genes are as follows: i) based on our previous experience with human lung disease studies [55], we found that a fold change of 1.2–1.3 is enough to elicit significant biological changes in humans; and ii) When using the threshold value of 1.3, 5,440 genes passed the filter, which we consider is a reasonable number for the downstream network analysis by *Treegl*. Then we applied *Treegl* to reconstruct gene networks in the five breast cell states using expression values of the qualified genes.

### A mathematical representation of the gene networks for the cell states in the phenotypic tree

We now give a mathematical formulation of representing the gene networks in order to introduce our algorithm. Consider the problem of modeling 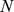 different gene networks, each corresponding to a unique cell state 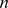. Each cell state 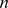 has 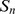 *i.i.d.* microarray replicates. All the arrays in the dataset have the same set of 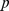 genes. In our case, we have 5 different conditions of the breast cells in the phenotypic tree: S1, T4, EGFR/ITGB1-T4R, PI3K/MAPKK-T4R, and MMP-T4R.

As commonly done, we model each gene network as a weighted undirected graph, where the vertices represent genes and the edges represent interactions in the network. Let 
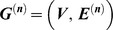
 represent a network in cell state 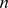, where 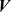 denotes the set of genes that is fixed for all cell states and 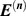 denotes the set of edges specific to the network for cell state 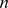. Let 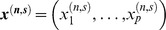 where 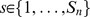 be the vector of expression values of genes on array 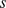 in cell state 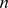. We assume 
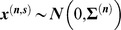
, i.e. that the vector of expression values follows a multivariate Gaussian distribution.

We are interested in reconstructing *a set of networks* 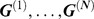 that are related by the phenotypic tree as shown in Figure 1. For each cell state 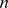, let 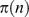 be the parent of the cell state in the tree; alternatively, we can also view 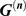 as a *descendant* of 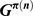. In our case, 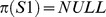, 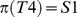, 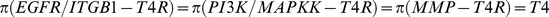. We generally let 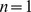 correspond to S1, 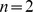 correspond to T4, and 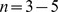 correspond to the EGFR/ITGB1-T4R group, the PI3K/MAPKK-T4R group, and the MMP-T4R group, respectively. Thus, in our formulation, recovering the structures of the gene regulatory networks in different breast cell states corresponds to estimating the network structure for each cell state.

### Estimating a gene network in a cell state

Consider first estimating the edge set of a single network 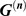 from the data. As described in the Results section, we model the gene network for each cell state 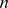 as a *Gaussian* Markov network. Therefore the inverse of the covariance matrix, called the precision matrix, 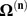, completely encodes the structure of the Markov network. An edge 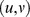 exists in the Markov network if and only if the corresponding precision matrix element is non-zero.

A Gaussian Markov network, encoded via the precision matrix, allows us to model more sophisticated dependencies than a correlation network, which is encoded by the covariance matrix. In particular, the precision matrix elements 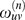 are related to the *partial* correlation between 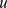 and 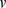 (denoted as 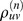, see below for details). Formally, partial correlation between a pair of random variables 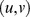 given a set 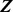 of controlling variables is defined as follows. Let 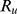 and 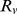 denote the residuals from performing linear regression of 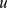 with 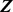 and 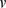 with 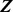, respectively. The partial correlation is then defined as the correlation between 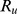 and 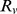. Unlike correlation, which simply measures the association between a pair of random variables, partial correlation intuitively measures the association between a pair of variables with a set 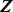 of controlling variables removed (where here 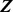 is all the other genes). The partial correlation, due to its close relationship with the elements of the precision matrix, makes the latter much more suitable than the covariance matrix for distinguishing between indirect and direct relationships as shown in Figure 2.

Since our goal is to learn the structure of the Markov network, we are only concerned with estimating which precision matrix elements are zero and which are not (rather than the exact precision matrix values). Therefore it suffices to estimate the partial correlation coefficients, which are proportional to the precision matrix elements by the equation 
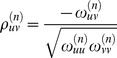
. An estimation algorithm can be constructed by exploiting the relationships between the partial correlation coefficients and a linear regression model [56]. Specifically, consider a linear regression model where gene 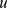 is treated as the response variable and all the other genes are covariates. The regression coefficient 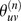 of covariate 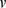 is then proportional to the partial correlation 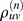.

The above facts enable us to use regression-based methods to estimate the elements of the precision matrix (up to a proportionality constant), and thus the underlying network structure. In particular, our method is based on an efficient neighborhood selection algorithm [26] based on 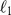-norm regularized regression that works well in practice and has strong theoretical guarantees. In this approach, the neighborhood of each gene 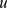 (the set of edges incident to 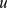) is estimated independently of the neighborhoods of other genes. After estimating each neighborhood, the results are then combined to produce the estimated network. In every neighborhood estimation step, gene 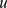 is treated as a response variable, and all the other genes are the covariates. An 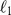 penalized linear regression (also known as the lasso [57]) is performed to give an estimate of the regression coefficients 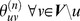.

Then by leveraging the relationship between the regression coefficients and the partial correlation, the estimated gene network 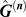 is constructed by adding an edge 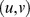 to 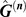 if either 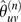 or 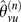 is non-zero (max-symmetrization).

### Estimating a tree-shaped genealogy of gene networks in the breast cells

Obviously, networks for each cell state can be estimated independently by using the method described above. However, this can lead to very poor estimates of the edge sets, because in common laboratory settings only a few replicates of gene expression data can be obtained. To overcome this limitation, we estimated the networks by assuming that the networks share similarities due to their relationships as suggested by the phenotypic tree, but also have some sharp differences. For example, for S1 and T4-2 cells, we assume they have considerable differences as the former is nonmalignant while the latter is tumorigenic; however, since T4-2 were derived from S1, we also assume that these cells share substantial similarity. This is the motivation behind *Treegl*, the algorithm that we first presented in [24].

*Treegl* is unique in that it makes use of a total variation regularizer, which allows information to be shared across different cell states, and thus encourages the resulting networks to be similar while allowing differences in the networks to be revealed. More specifically, *Treegl* adopts the idea of neighborhood selection and additionally penalizes the differences between the neighborhoods of adjacent states in the breast cell phenotypic tree. This makes *Treegl* more effective in small-sample-size settings than existing approaches since it can estimate a collection of networks more robustly by leveraging the similarities among them.

In summary, *Treegl* proposes the following optimization problem for jointly recovering the neighborhoods of genes for all the cell states in the phenotypic tree of the breast cells:
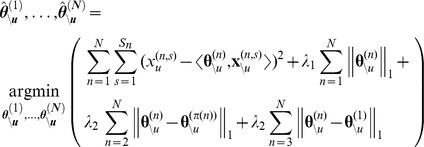



In the equation above, the first term corresponds to the residual sum of squares as in normal linear regression. 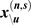 indicates the 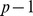 vector of the expression values of all genes except 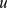, and similarly, 
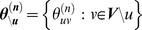
. 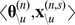 is defined as 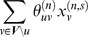. The second term (corresponding to 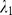) is a 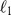 penalty on the edge weights (similar to [26]), where 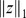 denotes the 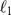 norm of vector 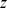, which is the sum of the absolute values of the components of 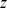. This penalty promotes **sparsity** in the edge weights by enforcing most of the edge weights to be zero. The assumption of sparsity is biologically justifiable. For example, it is common to find a transcription factor regulating a limited number of genes under specific conditions [31]. The details of the 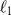 regularization can be seen in [57]. The third term (also called the total variation penalty) associated with 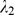 enforces sparsity of differences between S1 and T4-2 as well as between T4-2 and each of the T4R groups (as illustrated in the tree structure in Figure 1), but not between T4Rs and S1. This encourages many (but not all) of the elements of 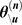 to be identical to those of 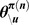. The fourth term (also associated with 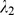) additionally penalizes the differences between each of the T4R groups and S1, while allowing for sharp differences to be revealed between the two groups. Note that if the fourth term was not used, the T4R networks would be biased to be more similar to the T4-2 network than the S1 network. This would be undesirable, since it is unknown a priori whether each of the T4R states are more similar to T4-2 or S1 cells.


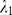
 and 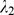 are regularization parameters that control the amount of penalization (see below for details on how we selected these parameters). Because the minimization problem is convex, we solved it using the CVX solver [58], as we described in [24].

In this work, we focus on genes linked by positive edges, because interaction of these genes is easier to interpret. For example, suppose genes *X* and *Y* are linked by positive edges, and genes *Y* and *Z* are also linked by positive edges. Intuitively, this suggests that genes *X* and *Y* are regulated in the same direction, that is, when gene *X* is up (or down)-regulated, gene *Y* is also up (or down)-regulated. Same is true for genes *Y* and *Z* which are also regulated in the same direction. As a result, we can also decide that genes *X* and *Z* are regulated in the same direction.

On the other hand, interpreting interaction of genes linked by negative edges is more complicated. For example, suppose genes *A* and *B* are linked by a negative edge and genes *B* and *C* also linked by a negative edge. This intuitively means that *A* and *B* are regulated in the opposite direction, and that *B* and *C* are also regulated in the opposite direction; it is, however, unclear what is the relationship of *A* and *C*, which may be regulated in either the same or the opposite direction.

Due to the reasons stated above, we chose to limit the scope of this work by focusing only on the positive edges to simplify our interpretation of the results.

### Selecting regularization parameters

Choosing the regularization parameters 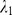 and 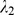 is a challenging problem in high dimensional statistics. Kolar and Xing proposed to use the Bayesian information criterion (BIC) score to select these parameters [59]. This approach can be useful in low dimensional settings; however, it does not perform well in high dimensional settings [60]. In this work, since we have a good knowledge of the biological properties of the S1 and T4-2 cells in the HTM3522 system, we employed a knowledge-based approach to tune 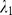 and 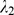, namely, we tuned these parameters based on our prior knowledge about S1 and T4-2 cells, which turned out to be highly effective in the high dimensional, small sample size setting as we encountered in this work. Specifically, we first varied 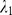 and 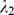 in the set {4, 4.5, 5, 5.5, 6, 6.5, 7} and the set {0.5, 1, 1.5, 2, 2.5}, respectively, and generated cell-state-specific networks for each possible pair of 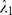 and 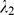. These sets of 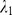 and 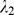 were chosen because the networks can be generated with reasonable sparsity. Then we examined the biological pathways significantly enriched in the differential network of the S1 and T4-2 cells, and found that when 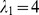 and 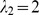, almost all of the enriched pathways in the T4-2 network make the best biological sense in that they are either well described in previous studies or are known pathways active in cancers. Since we used S1 and T4-2 cells to help tune the regularization parameters, we present and discuss mainly biological findings we made from the networks of the T4-2 reversion cells to avoid circular reasoning.

### Synthetic network generation and evaluation

We describe below how the networks in our simulation experiments were generated. Consider the following artificial collection of 70 networks, related by a tree:

1. A network A with 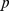 nodes, with an average degree 4, and maximum degree 6 is randomly generated. For the first 10 states, 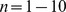, ***A*** remains unchanged, and thus, the networks for cell states 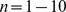 are identical.
2. After 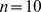, the network branches into two child networks, ***B*** and ***C***. To generate each child network, 25% of the edges are randomly deleted and the same number of the edges are randomly added. This represents a sharp, sparse change in the network. These child networks remain unchanged for another 10 states (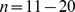 for ***B***, 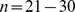 for ***C***).
3. ***B*** and ***C*** then branch (similar to step 2) to generate networks ***D*** and ***E*** from ***B***, and ***F*** and ***G*** from ***C***. These networks remain unchanged for another 10 states 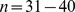 for ***D***, 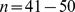 for ***E***, 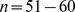 for ***F***, and 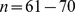 for ***G***.

*Treegl* does not know a priori which networks are identical and which are not. The number (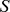) of samples are then generated for each network under the Gaussian Graphical Model assumption. We vary the values of 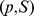 in the simulation experiments, and the results presented in Figures 3 are based on the values indicated in the figure. In each scenario, the number of edges is twice as much as the number of nodes.

To evaluate *Treegl*, we conduct a total of 10 simulation experiments, and plot the precision-recall curves showing the recall for different values of precision based on the networks reconstructed by *Treegl*. The error bars in the curves indicate the first and third quartiles of the results. Details on how we generated the precision-recall curves and selected the regularization parameters can be found in [24].

### Pathway analysis

To identify pathways significantly enriched in the gene networks of the 5 breast cell states estimated by *Treegl*, we performed pathway analysis on the list of the genes involved in each network using the *Category* Bioconductor package with minor modification (http://www.bioconductor.org). The *Category* package uses hypergeometric tests to assess overrepresentation of the KEGG pathways among genes of interest. A list of 12,977 unique genes on the Affymetrix GeneChip Human Genome U133A was used as the reference gene list for the pathway analysis. A pathway is considered to be significant if *p*<0.1 with the FDR controlling procedure of Benjamini & Hochberg [61].

### Disease relevance analysis

To find out genes significantly associated with certain diseases in the differential networks of the breast cell states, we performed pathway analysis as described above. For each differential network, pathways related to diseases and significantly enriched in the network were singled out; genes in the network that are involved in the enriched disease-related pathways were reported as the genes significantly associated with the diseases in the network.

### GO analysis

To identify functional groups of genes significantly enriched in the gene networks of the breast cells estimated by *Treegl*, we performed GO analysis on the list of the genes involved in each network using the *GOstat* program [62]. The *GOstat* program finds the enriched functional groups using Fisher's exact tests. The *GOstat* program was also used to identify functional groups of genes enriched among the neighborhoods (or the subnetworks) of the hubs significantly affecting patient survival. A functional group is considered to be significant if *p*<0.05 with the FDR controlling procedure of Benjamini & Hochberg. A list of 12,977 unique genes on the Affymetrix GeneChip Human Genome U133A was used as the reference gene list for the *GOstat* program.

### Survival analysis of hubs

We define hubs as genes with positive degree greater than 5 in the differential networks of the breast cell states. Survival analysis was performed using microarray expression values of the hubs extracted from a gene expression microarray data set obtained from 295 primary human breast tumors [39]. For each hub, its expression values across all patients were divided into three groups: lower quartile, interquartile, and upper quartile groups. Kaplan–Meier curves were used to estimate the association of expression values of the hubs in the three groups with patient survival. The log-rank test was used to calculate *p*-values of the survival curves. A hub was considered as significant if the p value of its associated survival curve <0.05 after controlling for multiple testing using the Bonferroni procedure.

## Supporting Information

Figure S1 Diagram depicting relationship of the networks in the simulation experiment. Networks with the same color have an identical network structure, i.e., networks 1–10 are identical, networks 11–20 are identical, etc. The black solid lines represent change-points where the network structure changes.
(EPS)

Figure S2 Precision-recall plots for each of the 70 individual networks in the simulation experiment for 50 nodes, 10 samples per network as shown in Figure 3D. Row 1 represents networks 1–10, Row 2 represents networks 11–20, Row 3 represents networks 21–30, …, and Row 7 represents networks 61–70. Treegl is shown in blue. The static approach of pooling all the samples to estimate only one network is shown in red, while the results for estimating each network independently without any information sharing is shown in green. The scales of the x-axis and y-axis are identical to those in Figure 3.
(EPS)

Figure S3 (Figure from [24]). An overview of the identified cell-state-specific networks: (A) S1, (B) T4, (C) EGFR/ITGB1-T4R, (D) PI3K/MAPKK-T4R, and (E) MMP-T4R. Only edges of absolute weight >0.1 are shown. Hubs (i.e., nodes with >5 edges) are in orange and enlarged proportional to their degrees.
(EPS)

Figure S4 A KEGG diagram of the phosphatidylinositol signaling pathway enriched in the differential network of the EGFR/ITGB1-T4R cells. PI3K is identified by red arrows. Only a section of the pathway is shown.
(EPS)

Figure S5 A KEGG diagram of the mTOR signaling pathway. This pathway is enriched in the differential networks of both the EGFR/ITGB1-T4R cells and the PI3K/MAPKK-T4R cells. PI3K and mTOR are identified by red and blue arrows, respectively. Insulin signaling pathway and INS/IGF are identified by purple and pink arrows, respectively. Notice that IGF is intimately connected with both insulin and mTOR pathways.
(EPS)

Figure S6 A KEGG diagram of the Insulin signaling pathway enriched in the differential network of the PI3K/MAPKK-T4R cells. PI3K, mTOR, and INS/IGF are identified by red, blue, and pink arrows, respectively.
(EPS)

Figure S7 A plot showing the number of genes that have degree d for various values of d. The plot in the inset displays the same data, except that the y-axis is shown in log scale. The red arrow points to the number of the genes with degree = 6. Since comparing to genes with degree = 5, there is a noticeable decreased number of genes with degree = 6, thus we designate all the genes with degree >5 to be hubs.
(EPS)

Table S1 Significantly enriched pathways in the differential networks of the breast cell states in the progression and reversion model of the HMT3522 cells. (A) S1 differential network; (B) T4-2 differential network; (C) EGFR/ITGB1-T4R differential network; (D) PI3K/MAPKK-T4R differential network; (E) MMP-T4R differential network.
(DOCX)

Table S2 Significantly enriched GO groups in the differential networks of the breast cell states in the progression and reversion model of the HMT3522 cells. (A) S1 differential network; (B) T4-2 differential network; (C) EGFR/ITGB1-T4R differential network; (D) PI3K/MAPKK-T4R differential network; (E) MMP-T4R differential network.
(DOCX)

Table S3 Diseases significantly associated with the genes in the differential networks of the breast cell states in the progression and reversion model of the HMT3522 cells. (A) S1 differential network; (B) T4-2 differential network; (C) EGFR/ITGB1-T4R differential network; (D) PI3K/MAPKK-T4R differential network; (E) MMP-T4R differential network.
(DOCX)

Table S4 Hubs in the differential networks of the breast cell states significantly affecting survival of breast cancer patients.
(DOCX)
