# Supplementary material for: Network Analysis of Breast Cancer Progression and Reversal Using a Tree-Evolving Network Algorithm
Source: PLoS Comput Biol. 2014 Jul 24;10(7):e1003713. doi: 10.1371/journal.pcbi.1003713 (PMC4109850; doi:10.1371/journal.pcbi.1003713)
Supplement: Table S1 — Significantly enriched pathways in the differential networks of the breast cell states in the progression and reversion model of the HMT3522 cells. (A) S1 differential network; (B) T4-2 differential network; (C) EGFR/ITGB1-T4R differential network; (D) PI3K/MAPKK-T4R differential network; (E) MMP-T4R differential network. (DOCX) [file pcbi.1003713.s008.docx]

Table S1: Significantly enriched pathways in the differential networks of the breast cell states in the progression and reversion model of the HMT3522 cells.

(A) Significantly enriched pathways in the S1 differential network.

| KEGGID | Term | Pvalue | pFDR | Odds Ratio | Exp Count | Count | Size | Genes |
| --- | --- | --- | --- | --- | --- | --- | --- | --- |
| 280 | Valine, leucine and isoleucine degradation | 7.90E-06 | 0.0017 | 5.08 | 4.32 | 15 | 40 | ABAT, ACADSB, ACAT1, ALDH2, AOX1, ALDH7A1, BCKDHA, DLD, HADHB, OXCT1, PCCA, ACAA2, HIBCH, ACAD8, MCCC2 |
| 4110 | Cell cycle | 8.80E-05 | 0.0092 | 2.61 | 11.99 | 26 | 111 | ATM, ATR, CCNH, CDC25B, CDKN1C, GADD45A, MCM2, MCM3, MCM4, MCM5, MCM6, MCM7, PRKDC, RB1, YWHAB, YWHAE, YWHAZ, SMC1A, CDC7, CUL1, SMC3, CCNE2, STAG2, GADD45G, YWHAQ, ANAPC5 |
| 3030 | DNA replication | 2.30E-04 | 0.0164 | 4.62 | 3.35 | 11 | 31 | FEN1, MCM2, MCM3, MCM4, MCM5, MCM6, MCM7, POLA1, RFC1, RPA1, POLA2 |
| 20 | Citrate cycle (TCA cycle) | 4.30E-04 | 0.0223 | 4.66 | 3.02 | 10 | 28 | ACLY, DLD, IDH3A, IDH3B, MDH1, PCK2, SDHA, SUCLG2, SUCLG1, SUCLA2 |
| 190 | Oxidative phosphorylation | 1.90E-03 | 0.0682 | 2.25 | 10.8 | 21 | 100 | ATP5I, ATP6V1B2, ATP6V1C1, COX6C, COX7C, COX15, CYC1, NDUFA3, NDUFA10, NDUFB3, NDUFC1, NDUFS1, NDUFS2, NDUFS3, NDUFS4, PPA1, SDHA, UQCRC1, UQCRC2, ATP5H, NDUFB11 |
| 640 | Propanoate metabolism | 1.90E-03 | 0.0682 | 3.97 | 3.02 | 9 | 28 | ABAT, ACAT1, ALDH2, ALDH7A1, PCCA, SUCLG2, SUCLG1, SUCLA2, HIBCH |
| 970 | Aminoacyl-tRNA biosynthesis | 2.60E-03 | 0.0768 | 3.77 | 3.13 | 9 | 29 | IARS, NARS, QARS, SARS, FARS2, LARS2, LARS, DARS2, TARS2 |
| 1100 | Metabolic pathways | 3.80E-03 | 0.099 | 1.37 | 95.89 | 119 | 888 | NAT1, ABAT, ACADSB, ACADVL, ACAT1, ACLY, ADH1B, ADK, AGL, AHCY, ALDH2, ALOX15B, AOX1, ATIC, ALDH7A1, ATP5I, ATP6V1B2, ATP6V1C1, BCKDHA, CAD, CBS, CDA, CDS1, COX6C, COX7C, COX15, CPOX, CPS1, CYC1, DHFR, DLD, DNMT1, DUT, FAH, GALNT2, GALNT3, GAMT, GBE1, GLUD1, GOT1, GOT2, GSS, HADHB, HK1, HMGCR, IDH3A, IDH3B, INPP5A, LTA4H, MDH1, NDUFA3, NDUFA10, NDUFB3, NDUFC1, NDUFS1, NDUFS2, NDUFS3, NDUFS4, PAFAH1B1, PCCA, PCK2, PFAS, PFKL, PLCB4, PLCD1, POLA1, POLR2I, PRPS2, PTS, ALDH18A1, QARS, SDHA, ST6GAL1, TKT, TSTA3, UGDH, UQCRC1, UQCRC2, UROD, MOGS, PPAP2A, PPAP2B, DGAT1, GPAA1, CDS2, SUCLG2, SUCLG1, SUCLA2, DPM1, SGPL1, ACAA2, ATP5H, POLR3C, AHCYL1, SLC27A5, LIAS, SEPHS2, NT5C2, POLA2, PHGDH, HIBCH, ACAD8, ALG5, POLR1D, CRYL1, OXSM, NADSYN1, PI4K2A, GALNT10, AKR1B10, MCCC2, NADK, PANK3, ALG9, ST6GALNAC5, MBOAT2, DGKH, PRPS1L1, MTHFD2L |

(B) Significantly enriched pathways in the T4 differential network.

| KEGGID | Term | Pvalue | pFDR | Odds Ratio | Exp Count | Count | Size | Genes |
| --- | --- | --- | --- | --- | --- | --- | --- | --- |
| 4510 | Focal adhesion | 1.20E-05 | 0.002 | 2.41 | 18.73 | 38 | 186 | ACTB, ACTG1, ACTN1, CAPN2, CAV2, COL4A1, COL4A2, FLNB, FN1, GSK3B, HRAS, ILK, ITGA6, ITGA2, ITGA5, ITGAV, ITGB4, ITGB6, JUN, LAMA3, LAMB3, LAMC2, MET, PAK1, PIK3R1, PPP1CA, MAP2K1, PXN, RAC1, RAC2, RAP1B, SOS2, THBS1, VEGFA, VEGFC, ZYX, PIK3R3, PDGFC |
| 4810 | Regulation of actin cytoskeleton | 4.70E-04 | 0.0126 | 2.04 | 19.03 | 34 | 189 | ACTB, ACTG1, ACTN1, BDKRB1, BDKRB2, CD14, CFL1, FGF1, FN1, GNA12, HRAS, ITGA6, ITGA2, ITGA5, ITGAV, ITGB4, ITGB6, PAK1, PIK3R1, PPP1CA, MAP2K1, PXN, RAC1, RAC2, SLC9A1, SOS2, TMSB4X, PIP5K1A, PIK3R3, ARPC1B, CYFIP1, SSH1, PDGFC, ARPC5L |
| 5100 | Bacterial invasion of epithelial cells | 9.40E-04 | 0.0202 | 2.91 | 6.24 | 15 | 62 | ACTB, ACTG1, RHOG, CAV2, FN1, ILK, ITGA5, MET, PIK3R1, PXN, RAC1, PIK3R3, DNM1L, ARPC1B, ARPC5L |
| 4620 | Toll-like receptor signaling pathway | 2.80E-03 | 0.0447 | 2.26 | 9.67 | 19 | 96 | CD14, CD86, FOS, IFNAR2, IL8, JUN, NFKB1, PIK3R1, MAP2K1, RAC1, RELA, CCL5, MAP2K4, TLR2, TRAF6, PIK3R3, FADD, TLR6, TICAM1 |
| 520 | Amino sugar and nucleotide sugar metabolism | 4.20E-03 | 0.0623 | 3.13 | 3.93 | 10 | 39 | GALE, GFPT1, GPI, PGM3, PMM2, UAP1, GNE, NANS, CMAS, UXS1 |
| 4666 | Fc gamma R-mediated phagocytosis | 5.30E-03 | 0.0724 | 2.28 | 8.05 | 16 | 80 | CFL1, PAK1, PIK3R1, PLD1, PRKCD, MAP2K1, RAC1, RAC2, PIP5K1A, PIK3R3, ASAP2, SPHK1, DNM1L, ARPC1B, MARCKSL1, ARPC5L |
| 4012 | ErbB signaling pathway | 6.00E-03 | 0.0747 | 2.24 | 8.16 | 16 | 81 | ABL2, HBEGF, EIF4EBP1, EREG, GSK3B, HRAS, JUN, MYC, PAK1, PIK3R1, MAP2K1, MAP2K4, SOS2, TGFA, NCK2, PIK3R3 |
| 100 | Steroid biosynthesis | 6.50E-03 | 0.0747 | 4.51 | 1.81 | 6 | 18 | CYP27B1, DHCR7, FDFT1, MSMO1, SC5D, NSDHL |
| 4662 | B cell receptor signaling pathway | 7.70E-03 | 0.081 | 2.31 | 6.95 | 14 | 69 | FOS, GSK3B, HRAS, JUN, NFKB1, NFKBIE, PIK3R1, MAP2K1, RAC1, RAC2, RELA, SOS2, PIK3R3, BCL10 |
| 4010 | MAPK signaling pathway | 8.00E-03 | 0.081 | 1.63 | 24.06 | 36 | 239 | FAS, ATF4, CACNB3, CASP3, CD14, DUSP2, DUSP5, DUSP6, DUSP7, FGF1, FLNB, FOS, GNA12, MKNK2, HRAS, IL1A, JUN, JUND, MYC, NFKB1, PAK1, MAP2K1, RAC1, RAC2, RAP1B, RASA2, RELA, RPS6KA3, MAP2K4, SOS2, TGFB1, TGFBR1, TRAF6, MAP4K4, ZAK, DUSP22 |
| 4145 | Phagosome | 8.80E-03 | 0.0853 | 1.88 | 12.99 | 22 | 129 | ACTB, ACTG1, CD14, HLA-E, ITGA2, ITGA5, ITGAV, NCF2, OLR1, RAB5A, RAC1, TFRC, THBS1, TLR2, TUBA4A, TUBB2A, TLR6, SEC61G, SEC61A1, TUBAL3, TUBB6, TUBB |
| 4722 | Neurotrophin signaling pathway | 9.90E-03 | 0.091 | 1.92 | 11.58 | 20 | 115 | ARHGDIB, ATF4, GSK3B, HRAS, JUN, NFKB1, NFKBIE, PDK1, PIK3R1, PRKCD, MAP2K1, RAC1, RAP1B, RELA, RPS6KA3, SOS2, TRAF6, PIK3R3, SH2B3, ZNF274 |

(C) Enriched pathways in the EGFR/ITGB1-T4R differential network.

| KEGGID | Term | Pvalue | pFDR | Odds Ratio | Exp Count | Count | Size | Genes |
| --- | --- | --- | --- | --- | --- | --- | --- | --- |
| 4722 | Neurotrophin signaling pathway | 0.0027 | N.S. | 2.41 | 7.456 | 16 | 115 | ABL1, AKT1, RHOA, ARHGDIA, CALML3, KRAS, NFKB1, NFKBIA, PIK3R2, PSEN1, RPS6KA1, RPS6KA2, RPS6KA3, YWHAB, RPS6KA5, MAGED1 |
| 4960 | Aldosterone-regulated sodium reabsorption | 0.0099 | N.S. | 3.31 | 2.464 | 7 | 38 | ATP1A1, ATP1B3, SFN, KRAS, PIK3R2, SCNN1B, NEDD4L |
| 4962 | Vasopressin-regulated water reabsorption | 0.0114 | N.S. | 3.21 | 2.529 | 7 | 39 | AQP3, ARHGDIA, DCTN1, DYNC1H1, NSF, DYNLL1, CREB3L2 |
| 4520 | Adherens junction | 0.0231 | N.S. | 2.36 | 4.214 | 9 | 65 | ACTN4, RHOA, CDH1, TCF7L2, TJP1, WASF1, WASF2, BAIAP2, PARD3 |
| 4114 | Oocyte meiosis | 0.0238 | N.S. | 2.13 | 5.641 | 11 | 87 | CALML3, ITPR3, PPP2R5E, RPS6KA1, RPS6KA2, RPS6KA3, SKP1, YWHAB, SMC1A, SLK, ANAPC2 |
| 4150 | mTOR signaling pathway | 0.027 | N.S. | 2.63 | 2.983 | 7 | 46 | AKT1, PIK3R2, RPS6KA1, RPS6KA2, RPS6KA3, ULK1, ULK2 |
| 4070 | Phosphatidyl-inositol signaling system | 0.0354 | N.S. | 2.16 | 4.539 | 9 | 70 | CALML3, INPPL1, ITPR3, PIK3R2, PLCD1, DGKD, PIP5K1C, PIP4K2C, PIKFYVE |
| 4360 | Axon guidance | 0.0377 | N.S. | 1.9 | 6.808 | 12 | 105 | ABL1, RHOA, EFNA3, EPHB4, EPHB6, KRAS, ABLIM1, SEMA3F, SEMA3E, SEMA4D, NFAT5, RHOD |
| 450 | Seleno-compound metabolism | 0.0383 | N.S. | 4.85 | 0.778 | 3 | 12 | CCBL1, MARS, MTR |
| 564 | Glycero-phospholipid metabolism | 0.0416 | N.S. | 2.21 | 3.955 | 8 | 61 | PLA2G6, DGKD, PPAP2C, PTDSS1, AGPAT2, GPD1L, PISD, ETNK2 |

(D) Enriched pathways in the PI3K/MAPKK-T4R differential network.

| KEGGID | Term | Pvalue | pFDR | Odds Ratio | Exp Count | Count | Size | Genes |
| --- | --- | --- | --- | --- | --- | --- | --- | --- |
| 4142 | Lysosome | 1.20E-05 | 0.0026 | 3.06 | 10.084 | 25 | 105 | ACP2, ASAH1, ATP6AP1, SCARB2, CD63, TPP1, CLN3, CLTB, FUCA1, GAA, GALC, GM2A, GNS, HYAL1, LAMP1, MAN2B1, SLC11A2, CTSA, PPT1, PSAP, AP1S2, AP3D1, AP3M2, LAMP3, MCOLN1 |
| 4612 | Antigen processing and presentation | 7.40E-04 | 0.0779 | 3.13 | 5.474 | 14 | 57 | CANX, CD74, CREB1, HLA-A, HLA-C, HLA-E, HLA-F, HLA-G, NFYB, PSME2, RFX5, TAPBP, TNF, IFI30 |
| 310 | Lysine degradation | 3.60E-03 | N.S. | 3.19 | 3.842 | 10 | 40 | ALDH9A1, ALDH3A2, DLST, GCDH, HADH, SETMAR, SUV39H1, SETD2, SUV420H1, NSD1 |
| 4722 | Neurotrophin signaling pathway | 5.90E-03 | N.S. | 2.03 | 11.044 | 20 | 115 | ABL1, BAD, CALML3, MAPK14, IRAK1, MAP3K5, NFKBIE, PDK1, PIK3CA, PIK3CD, PIK3R2, PLCG2, PSEN1, RAP1A, RPS6KA1, TP73, IRS2, MAGED1, FRS2, KIDINS220 |
| 5145 | Toxoplasmosis | 1.40E-02 | N.S. | 1.88 | 11.141 | 19 | 116 | BIRC3, BAD, CHUK, MAPK14, GNAI1, IFNGR2, IRAK1, JAK1, LAMB2, MYD88, PDK1, PIK3CA, PIK3CD, PIK3R2, TNF, TNFRSF1A, TYK2, PLA2G6, LAMB4 |
| 4120 | Ubiquitin mediated proteolysis | 1.40E-02 | N.S. | 1.88 | 11.141 | 19 | 116 | BIRC3, MID1, SKP1, TCEB2, UBE2E1, CUL3, PIAS1, CDC16, HERC1, PIAS2, UBE2L6, UBE3C, KEAP1, UBA2, HUWE1, UBE4B, FBXO2, ANAPC2, UBE2D4 |
| 61 | Fatty acid biosynthesis | 1.40E-02 | N.S. | 9.47 | 0.576 | 3 | 6 | ACACA, ACACB, FASN |
| 4210 | Apoptosis | 1.70E-02 | N.S. | 2.06 | 7.587 | 14 | 79 | BIRC3, BAD, CAPN1, CASP6, CHUK, IRAK1, MYD88, PIK3CA, PIK3CD, PIK3R2, TNF, TNFRSF1A, TNFSF10, CFLAR |
| 71 | Fatty acid metabolism | 2.50E-02 | N.S. | 2.54 | 3.649 | 8 | 38 | ACAA1, ALDH9A1, ALDH3A2, ECI1, GCDH, HADH, ACSL6, ACSL5 |
| 4150 | mTOR signaling pathway | 2.80E-02 | N.S. | 2.32 | 4.418 | 9 | 46 | PIK3CA, PIK3CD, PIK3R2, RPS6KA1, VEGFA, VEGFB, ULK1, ULK2, MLST8 |
| 4622 | RIG-I-like receptor signaling pathway | 4.30E-02 | N.S. | 1.94 | 6.243 | 11 | 65 | CHUK, MAPK14, CYLD, DDX3X, PIN1, TNF, ISG15, TANK, DDX58, RNF125, IFIH1 |
| 380 | Tryptophan metabolism | 4.50E-02 | N.S. | 2.38 | 3.361 | 7 | 35 | ALDH9A1, ALDH3A2, CCBL1, CYP1B1, GCDH, HADH, KYNU |
| 4910 | Insulin signaling pathway | 4.80E-02 | N.S. | 1.62 | 11.909 | 18 | 124 | ACACA, ACACB, BAD, CALML3, FASN, MKNK2, INSR, PHKA1, PHKA2, PIK3CA, PIK3CD, PIK3R2, PRKCI, PYGB, MKNK1, IRS2, TRIP10, RHOQ |

(E) Significantly enriched pathways in the MMP-T4R differential network.

| KEGGID | Term | Pvalue | pFDR | Odds Ratio | Exp Count | Count | Size | Genes |
| --- | --- | --- | --- | --- | --- | --- | --- | --- |
| 1100 | Metabolic pathways | 1.50E-06 | 0.0003 | 1.99 | 50.894 | 82 | 888 | NAT1, ACAA1, ACO1, ALAD, ALDH9A1, ALPP, ASAH1, ATP5G1, ATP6V1E1, BDH1, CBR1, DGKA, DHFR, ECHS1, EPHX2, EXTL2, FECH, GART, B4GALT1, GNS, HADH, HSD17B4, IDH1, IDH2, INPP1, MAT2A, ME1, NDUFA4, NDUFA6, NDUFA8, NDUFA9, NME1, NNMT, PAFAH1B3, PCCB, PDHB, PGD, PLA2G4A, POLR2K, POLR2L, PPT1, PRIM2, RRM1, ACSM3, SCP2, ST6GAL1, SPR, TKT, UGP2, UQCRFS1, DGKD, B4GALT3, ATP6V0D1, GRHPR, ATP5J2, PRDX6, PIGK, COQ7, AKR1A1, PEMT, PAICS, UQCR11, AHCYL2, HIBCH, TPK1, UQCRQ, ST6GALNAC4, PSAT1, LAP3, CMPK1, POLE3, PNPO, ETNK2, BDH2, CYP4F11, POLR1E, PANK3, ALG13, PTGES2, FLAD1, ACSM1, NDUFS7 |
| 3050 | Proteasome | 7.40E-05 | 0.0074 | 5.48 | 2.35 | 10 | 41 | PSMA2, PSMA3, PSMA4, PSMB8, PSMB9, PSMC2, PSMD7, PSME2, PSMD6, PSME3 |
| 3040 | Spliceosome | 5.40E-04 | 0.0207 | 2.98 | 5.789 | 15 | 101 | DDX5, HNRNPA1, SRSF1, SNRPC, SNRNP40, PPIH, SRSF10, TCERG1, SRSF8, NCBP2, LSM5, LSM4, TRA2A, MAGOHB, SF3B5 |
| 650 | Butanoate metabolism | 6.20E-04 | 0.0207 | 5.89 | 1.547 | 7 | 27 | BDH1, ECHS1, HADH, PDHB, ACSM3, BDH2, ACSM1 |
