# Supplementary material for: Network Analysis of Breast Cancer Progression and Reversal Using a Tree-Evolving Network Algorithm
Source: PLoS Comput Biol. 2014 Jul 24;10(7):e1003713. doi: 10.1371/journal.pcbi.1003713 (PMC4109850; doi:10.1371/journal.pcbi.1003713)
Supplement: Table S2 — Significantly enriched GO groups in the differential networks of the breast cell states in the progression and reversion model of the HMT3522 cells. (A) S1 differential network; (B) T4-2 differential network; (C) EGFR/ITGB1-T4R differential network; (D) PI3K/MAPKK-T4R differential network; (E) MMP-T4R differential network. (DOCX) [file pcbi.1003713.s009.docx]

**Table S2:** Significantly enriched GO groups in the differential networks of the breast cell states in the progression and reversion model of the HMT3522 cells.

(A) Significantly enriched GO groups in the S1 differential network.

| GO | Group count | Total count | Pvalue  (FDR) | GO as name | Genes |
| --- | --- | --- | --- | --- | --- |
| GO:0015980 | 22 | 59 | 4.63E-08 | energy derivation by oxidation of organic compounds; | sdha; ppp1r3d; gys1; suclg1; acadvl; idh3a; gbe1; agl; bcs1l; suclg2; uqcrc1; lepr; ndufs1; pygb; kiaa0100; oxa1l; sucla2; idh3b; mdh1; phka1; ppp1r3c; ndufs4 |
| GO:0044238 | 502 | 3984 | 2.50E-05 | primary metabolic process; | trex1; cul1; rad17; epha4; pck2; mcm6; usp14; acy1; icmt; crot; chst2; rb1; ufm1; cpt2; ctnnd1; wsb2; plcd1; gadd45g; supt7l; slc27a2; znf552; sucla2; bhlhb3; phka1; fryl; fdxr; cic; irx5; puf60; mrpl48; st13; gemin8; sult1e1; u1snrnpbp; kdr; rps9; ror1; znf432; pcnp; pigg; gins1; gmpr2; nmi; suclg1; rad51c; sertad2; hnrnpa2b1; nek7; galnt2; phgdh; cds1; alox15b; notch1; poln; eif6; top2b; rnf139; snx6; mizf; rad54b; gal; tsen34; itgb3bp; mafg; ppp1r3c; fah; gnpat; wdr5b; etf1; igf1r; mrps30; mapk10; ung; ube3c; pola1; znf32; ppwd1; aff4; ddx24; bccip; ints8; serpine2; ikzf5; asxl1; erh; smc2; ecd; fanci; pltp; irx4; rpl8; mmp28; flj20628; tjp1; mrpl44; chm; ptpla; rbm23; gtf3c3; txnl4b; sall1; plk2; znf141; f2r; mrpl13; esf1; sox9; csnk2a2; mrpl24; ascc3; ugdh; st6galnac5; rfc1; znf473; arpp-19; lonp1; rnf167; gtf2ird1; sirt1; hoxa2; mitf; ph-4; gin1; vrk3; phb2; twist1; arid5b; phf17; nosip; isoc2; sdha; prmt1; plod3; cyp1b1; cnot6; igfbp3; anapc5; tarbp1; rbbp8; polr1d; irf3; med23; uqcrc1; ptprz1; pfas; myd88; mdh1; ahcy; ddb2; serpina3; mcm5; znf226; snrpd2; maf1; kifap3; ttll1; eaf2; dclre1a; wasf3; fau; c1orf66; prkcz; inhbb; mms19; nfib; elp3; sertad3; cd81; mettl1; mafb; gadd45a; ppm1a; hadhb; msrb2; rps5; slc9a3r1; ctsl2; znf7; hipk2; adm; usp11; clk3; ube2m; dpysl2; mrpl23; nedd8; ech1; supv3l1; lepre1; ppp2r1a; sirt2; hipk1; ripk2; acadvl; lars2; slc2a4rg; suclg2; mcm4; cpne1; cbs; prkx; oxa1l; lrpap1; fhit; fbxl5; ptpn14; sumo3; pola2; zbtb16; rpa1; fars2; rps27l; ndufs4; cbll1; eif2s3; prkaa1; med4; slbp; rnpep; mrpl49; ptprm; bag5; cln5; eya1; ssbp2; ruvbl2; hsbp1; acaa2; hells; mipep; nt5c2; ncor1; akt3; pmpcb; znf230; hk1; c1orf25; chst1; slc27a5; irak1; msh2; usp3; pts; dhfr; phf21a; irf1; pcsk1; ints9; smyd3; ift88; znf304; uspl1; lars; ptpru; cpd; zscan22; znf350; flna; xrcc5; cnot7; lta4h; eif3k; gbe1; ppap2b; chd1; rcor3; qars; sephs2; dars2; prps1l1; prdx4; supt16h; sox15; bmi1; nfil3; rab13; apeh; tyw1; yaf2; march3; eef1b2; znf34; hoxa4; ccdc44; dcp1a; tars2; loc440434; trim5; znf623; dnajc1; atr; ndufs1; sars; mphosph8; znf395; gtf2f2; mark4; casp8ap2; mcm2; adnp2; sma4; prps2; anapc1; ubr5; gli3; fen1; rbmx; cops5; ccnb1ip1; eif4h; chfr; crnkl1; rnf7; ccnh; narfl; cstf3; znf323; gtf2h2; lgtn; ppp1r3d; manba; mtif2; atg7; znf250; hist2h2be; mtrf1; mcm3; proc; sall2; parp4; aph1a; rbck1; prmt8; erf; mrpl22; uimc1; znf394; mrpl34; kiaa0100; plod1; lrp8; ptk6; idh3b; ptpn13; znf331; nolc1; mybl1; amz2; znf16; znf223; got2; atpif1; prkg2; mis12; mphosph10; nadsyn1; znf211; c3; mme; rpl29; osgep; mid1; cda; rps16; p4hb; atp5h; sltm; atic; atp6v1b2; pygb; cad; ubac1; akr1b10; rnf128; prmt5; cct6b; c17orf81; ryk; sox12; pcaf; ppap2a; blmh; mrpl15; mpdu1; tp53bp1; aldh2; mmp14; psmd8; dis3; dhx15; idh3a; gcs1; prkacb; mccc2; phf20; tpr; eif3h; znf232; iars; hoxa5; rpp14; znf177; ctsk; hdac5; btg2; snrpb2; psip1; scand1; pabpc3; ndufa10; mkks; det1; tubd1; znf264; cdc7; cryl1; tcf4; zxda; hsf1; mynn; dnmt1; parp1; btaf1; ranbp9; cask; dpm1; plcb4; trps1; ola1; thoc1; calr; htra1; wdhd1; cux2; cpne3; slc27a3; apex1; pnpla4; ermp1; thrap3; mgea5; acad8; oxsm; mcm7; jak2; pdk4; pnkp; aldh18a1; nsmaf; cuedc2; gys1; sec11a; csnk2a1; alg5; elf1; exosc4; zbtb10; tsta3; cdc25b; prdm4; ugcgl1; st6galnac2; smc1a; meis3p1; dtl; trim28; pet112l; trim22; nr1h3; rcbtb1; ctnnbip1; baz1a; thg1l; yeats4; mrpl33; acly; polr2i; bckdha; fancc; gmfb; adk; nbn; rsl1d1; dgat1; dapk1; bcs1l; agl; taf2; gins2; gamt; chd8; got1; xpo7; foxc2; lnpep; hspbp1; ephb3; nars; hspa4l; acadsb; atm; klf9 |
| GO:0044237 | 501 | 3983 | 2.89E-05 | cellular metabolic process; | trex1; cul1; rad17; epha4; pck2; mcm6; usp14; acy1; icmt; crot; chst2; rb1; ufm1; cpt2; ctnnd1; wsb2; plcd1; gadd45g; supt7l; slc27a2; znf552; sucla2; bhlhb3; phka1; fryl; fdxr; cic; irx5; puf60; mrpl48; st13; gemin8; sult1e1; u1snrnpbp; kdr; rps9; ror1; znf432; pcnp; pigg; gins1; gmpr2; nmi; suclg1; rad51c; ndufs2; sertad2; hnrnpa2b1; nek7; galnt2; phgdh; cds1; alox15b; notch1; poln; eif6; top2b; rnf139; mpv17; snx6; mizf; rad54b; gal; tsen34; itgb3bp; mafg; ppp1r3c; fah; gnpat; wdr5b; etf1; igf1r; mrps30; mapk10; ung; ube3c; pola1; znf32; ppwd1; aff4; ddx24; bccip; ints8; serpine2; ikzf5; asxl1; erh; smc2; ecd; fanci; irx4; rpl8; mmp28; flj20628; mrpl44; chm; ptpla; rbm23; gtf3c3; txnl4b; sall1; plk2; ahcyl1; znf141; f2r; mrpl13; esf1; sox9; csnk2a2; mrpl24; ascc3; ugdh; st6galnac5; rfc1; znf473; arpp-19; lonp1; rnf167; gtf2ird1; sirt1; hoxa2; mitf; gin1; vrk3; phb2; twist1; arid5b; phf17; nosip; isoc2; sdha; prmt1; plod3; cyp1b1; lias; cnot6; igfbp3; anapc5; tarbp1; rbbp8; polr1d; irf3; med23; uqcrc1; ptprz1; mocs3; pfas; myd88; mdh1; ahcy; ddb2; mcm5; znf226; snrpd2; maf1; ttll1; eaf2; dclre1a; wasf3; fau; c1orf66; prkcz; inhbb; mms19; nfib; elp3; sertad3; cd81; mettl1; mafb; gadd45a; ppm1a; hadhb; msrb2; rps5; ctsl2; znf7; urod; hipk2; adm; usp11; clk3; ube2m; dpysl2; mrpl23; nedd8; ech1; supv3l1; ppp2r1a; sirt2; hipk1; ripk2; ca11; acadvl; lars2; slc2a4rg; suclg2; mcm4; cbs; prkx; lrpap1; ndufc1; fhit; fbxl5; ptpn14; sumo3; pola2; zbtb16; rpa1; fars2; rps27l; ndufs4; cbll1; eif2s3; prkaa1; med4; slbp; rnpep; mrpl49; ptprm; bag5; eya1; ssbp2; ruvbl2; hsbp1; acaa2; hells; mipep; nt5c2; ncor1; akt3; pmpcb; znf230; hk1; c1orf25; chst1; slc27a5; irak1; msh2; usp3; pts; dhfr; phf21a; irf1; pcsk1; ints9; smyd3; ift88; znf304; uspl1; lars; cpd; zscan22; znf350; ptpru; flna; xrcc5; cnot7; lta4h; eif3k; gbe1; ppap2b; chd1; rcor3; qars; sephs2; dars2; prps1l1; prdx4; supt16h; sox15; bmi1; nfil3; rab13; apeh; tyw1; yaf2; march3; eef1b2; znf34; hoxa4; ccdc44; dcp1a; tars2; ndufa3; loc440434; trim5; znf623; dnajc1; atr; ndufs1; sars; mphosph8; znf395; gtf2f2; mark4; casp8ap2; mcm2; adnp2; prps2; anapc1; ubr5; gli3; fen1; rbmx; cops5; ccnb1ip1; eif4h; chfr; crnkl1; rnf7; ccnh; narfl; cstf3; znf323; gtf2h2; lgtn; ppp1r3d; manba; mtif2; atg7; znf250; cpox; hist2h2be; mtrf1; mcm3; proc; sall2; parp4; aph1a; rbck1; prmt8; erf; mrpl22; uimc1; znf394; mrpl34; kiaa0100; lrp8; plod1; ptk6; idh3b; ptpn13; znf331; nolc1; mybl1; amz2; znf16; znf223; got2; atpif1; prkg2; mis12; mphosph10; nadsyn1; znf211; c3; mme; rpl29; osgep; ndufs3; mid1; cda; rps16; p4hb; atp5h; sltm; atic; atp6v1b2; pygb; cad; ubac1; akr1b10; rnf128; prmt5; cct6b; c17orf81; ryk; sox12; pcaf; ppap2a; blmh; mrpl15; mpdu1; tp53bp1; aldh2; mmp14; psmd8; dis3; oxct1; dhx15; idh3a; gcs1; prkacb; mccc2; phf20; tpr; eif3h; znf232; iars; hoxa5; rpp14; znf177; ctsk; hdac5; btg2; snrpb2; psip1; scand1; pabpc3; ndufa10; mkks; det1; tubd1; znf264; cdc7; cryl1; tcf4; zxda; hsf1; mynn; dnmt1; parp1; btaf1; cask; dpm1; trps1; ola1; thoc1; calr; aox1; htra1; wdhd1; cux2; slc27a3; apex1; ermp1; thrap3; mgea5; acad8; oxsm; mcm7; jak2; pdk4; pnkp; aldh18a1; nsmaf; cuedc2; gys1; sec11a; csnk2a1; alg5; elf1; exosc4; zbtb10; tsta3; cdc25b; prdm4; ugcgl1; st6galnac2; smc1a; meis3p1; dtl; trim28; pet112l; trim22; nr1h3; rcbtb1; ctnnbip1; baz1a; thg1l; yeats4; mrpl33; acly; polr2i; bckdha; fancc; gmfb; adk; nbn; ppa1; rsl1d1; dgat1; dapk1; agl; taf2; gins2; gamt; chd8; got1; xpo7; aldh1l1; foxc2; lnpep; hspbp1; ephb3; nars; hspa4l; acadsb; atm; klf9 |
| GO:0006139 | 266 | 1901 | 3.05E-05 | nucleobase, nucleoside, nucleotide and nucleic acid metabolic process; | trex1; rad17; mcm6; rb1; ctnnd1; gadd45g; supt7l; znf552; bhlhb3; fryl; cic; irx5; puf60; gemin8; u1snrnpbp; znf432; gins1; gmpr2; nmi; rad51c; hnrnpa2b1; sertad2; notch1; poln; top2b; mizf; snx6; rad54b; itgb3bp; tsen34; mafg; ung; pola1; ppwd1; znf32; aff4; bccip; ddx24; ints8; ikzf5; erh; asxl1; fanci; ecd; smc2; irx4; flj20628; mrpl44; rbm23; gtf3c3; txnl4b; sall1; znf141; esf1; sox9; ascc3; ugdh; rfc1; znf473; sirt1; gtf2ird1; hoxa2; mitf; gin1; twist1; phb2; arid5b; phf17; cnot6; polr1d; rbbp8; tarbp1; irf3; med23; pfas; myd88; ddb2; mdh1; znf226; mcm5; snrpd2; maf1; eaf2; dclre1a; c1orf66; mms19; sertad3; nfib; elp3; mettl1; mafb; gadd45a; znf7; hipk2; adm; dpysl2; ppp2r1a; supv3l1; sirt2; hipk1; ripk2; lars2; mcm4; slc2a4rg; fhit; pola2; zbtb16; fars2; rpa1; med4; slbp; ruvbl2; eya1; ssbp2; hsbp1; hells; nt5c2; ncor1; znf230; c1orf25; irak1; msh2; dhfr; irf1; phf21a; ints9; smyd3; znf304; lars; zscan22; znf350; flna; xrcc5; cnot7; chd1; rcor3; qars; dars2; prps1l1; supt16h; sox15; bmi1; nfil3; rab13; yaf2; tyw1; znf34; hoxa4; ccdc44; dcp1a; tars2; znf623; atr; ndufs1; sars; mphosph8; gtf2f2; znf395; casp8ap2; mcm2; adnp2; prps2; gli3; ubr5; fen1; rbmx; cops5; ccnh; crnkl1; narfl; cstf3; znf323; gtf2h2; znf250; hist2h2be; mcm3; sall2; parp4; prmt8; erf; uimc1; znf394; znf331; nolc1; mybl1; znf16; znf223; atpif1; mphosph10; nadsyn1; znf211; cda; atp5h; sltm; atic; atp6v1b2; cad; prmt5; c17orf81; sox12; pcaf; tp53bp1; dis3; dhx15; phf20; tpr; znf232; iars; hoxa5; rpp14; znf177; hdac5; btg2; snrpb2; psip1; scand1; pabpc3; ndufa10; znf264; tcf4; zxda; hsf1; mynn; parp1; dnmt1; btaf1; trps1; ola1; thoc1; calr; wdhd1; cux2; apex1; thrap3; acad8; mcm7; pnkp; elf1; exosc4; zbtb10; tsta3; prdm4; smc1a; meis3p1; dtl; trim28; trim22; nr1h3; rcbtb1; ctnnbip1; baz1a; thg1l; yeats4; acly; polr2i; bckdha; fancc; adk; nbn; gins2; taf2; chd8; foxc2; nars; klf9; atm |
| GO:0016874 | 41 | 182 | 7.14E-05 | ligase activity; | nosip; lars; lias; ube3c; anapc5; lars2; suclg2; rcl1; qars; mccc2; dars2; tpr; slc27a2; pet112l; iars; mocs3; pfas; sucla2; ddb2; fbxl5; march3; ttll1; fars2; nadsyn1; cbll1; tars2; mid1; suclg1; trim5; sars; cad; rnf139; rnf128; rnf167; nars; slc27a5; ubr5; slc27a3; ccnb1ip1; chfr; ube2m |
| GO:0043283 | 341 | 2569 | 7.85E-05 | biopolymer metabolic process; | trex1; cul1; rad17; epha4; mcm6; usp14; icmt; rb1; ufm1; ctnnd1; wsb2; gadd45g; supt7l; znf552; bhlhb3; phka1; fryl; cic; irx5; puf60; gemin8; u1snrnpbp; kdr; ror1; pcnp; znf432; gins1; pigg; nmi; rad51c; hnrnpa2b1; sertad2; nek7; galnt2; notch1; poln; top2b; rnf139; mizf; rad54b; tsen34; itgb3bp; mafg; ppp1r3c; wdr5b; igf1r; mapk10; ung; pola1; ube3c; ppwd1; znf32; aff4; bccip; ddx24; ints8; ikzf5; asxl1; fanci; ecd; smc2; irx4; flj20628; chm; mrpl44; rbm23; gtf3c3; txnl4b; sall1; plk2; znf141; f2r; esf1; sox9; csnk2a2; ascc3; st6galnac5; rfc1; znf473; rnf167; gtf2ird1; sirt1; hoxa2; mitf; gin1; vrk3; phb2; twist1; arid5b; phf17; nosip; prmt1; plod3; cnot6; igfbp3; anapc5; rbbp8; tarbp1; irf3; med23; ptprz1; myd88; ddb2; znf226; mcm5; snrpd2; maf1; ttll1; eaf2; dclre1a; fau; prkcz; c1orf66; mms19; sertad3; elp3; nfib; cd81; mettl1; mafb; gadd45a; ppm1a; znf7; hipk2; usp11; ube2m; clk3; nedd8; supv3l1; ppp2r1a; sirt2; hipk1; ripk2; lars2; slc2a4rg; mcm4; prkx; fbxl5; sumo3; ptpn14; pola2; zbtb16; rpa1; fars2; ndufs4; cbll1; prkaa1; med4; slbp; ptprm; cln5; eya1; ruvbl2; hsbp1; hells; mipep; ncor1; akt3; znf230; c1orf25; chst1; irak1; msh2; usp3; phf21a; irf1; ints9; smyd3; znf304; uspl1; lars; zscan22; znf350; ptpru; cnot7; xrcc5; gbe1; chd1; rcor3; qars; dars2; prdx4; bmi1; supt16h; sox15; rab13; nfil3; yaf2; tyw1; march3; eef1b2; znf34; hoxa4; ccdc44; dcp1a; tars2; trim5; znf623; atr; sars; mphosph8; gtf2f2; znf395; mcm2; mark4; casp8ap2; adnp2; anapc1; gli3; ubr5; fen1; rbmx; cops5; ccnb1ip1; chfr; ccnh; rnf7; crnkl1; cstf3; znf323; gtf2h2; ppp1r3d; manba; atg7; znf250; hist2h2be; mcm3; sall2; parp4; aph1a; rbck1; prmt8; znf394; uimc1; erf; ptk6; plod1; nolc1; znf331; ptpn13; mybl1; znf16; znf223; prkg2; mphosph10; znf211; mid1; p4hb; sltm; pygb; ubac1; prmt5; rnf128; c17orf81; ryk; sox12; pcaf; ppap2a; mpdu1; tp53bp1; mmp14; dis3; dhx15; gcs1; prkacb; tpr; phf20; znf232; iars; hoxa5; rpp14; znf177; hdac5; btg2; snrpb2; psip1; scand1; pabpc3; det1; znf264; cdc7; tcf4; zxda; hsf1; mynn; dnmt1; parp1; cask; dpm1; trps1; thoc1; calr; wdhd1; cux2; apex1; oxsm; acad8; thrap3; mcm7; pdk4; jak2; pnkp; gys1; sec11a; cuedc2; csnk2a1; alg5; elf1; exosc4; tsta3; zbtb10; cdc25b; prdm4; ugcgl1; st6galnac2; smc1a; trim28; dtl; meis3p1; trim22; nr1h3; rcbtb1; ctnnbip1; baz1a; thg1l; yeats4; polr2i; fancc; bckdha; gmfb; nbn; dapk1; agl; gins2; taf2; chd8; foxc2; ephb3; nars; atm; klf9 |
| GO:0043170 | 437 | 3437 | 0.000108 | macromolecule metabolic process; | trex1; cul1; rad17; epha4; pck2; mcm6; usp14; acy1; icmt; chst2; rb1; ufm1; ctnnd1; wsb2; gadd45g; supt7l; znf552; sucla2; bhlhb3; phka1; fryl; cic; irx5; puf60; mrpl48; st13; gemin8; u1snrnpbp; kdr; rps9; ror1; znf432; pcnp; pigg; gins1; nmi; suclg1; rad51c; sertad2; hnrnpa2b1; nek7; galnt2; notch1; poln; eif6; top2b; rnf139; mizf; rad54b; tsen34; itgb3bp; mafg; ppp1r3c; wdr5b; igf1r; etf1; mapk10; mrps30; ung; pola1; ube3c; ppwd1; znf32; aff4; ddx24; bccip; ints8; serpine2; ikzf5; asxl1; smc2; ecd; fanci; irx4; rpl8; mmp28; flj20628; tjp1; mrpl44; chm; ptpla; rbm23; gtf3c3; txnl4b; sall1; plk2; znf141; f2r; mrpl13; esf1; sox9; csnk2a2; mrpl24; ascc3; ugdh; st6galnac5; rfc1; znf473; arpp-19; lonp1; rnf167; gtf2ird1; sirt1; hoxa2; mitf; ph-4; gin1; vrk3; phb2; twist1; arid5b; phf17; nosip; prmt1; sdha; isoc2; plod3; cnot6; igfbp3; anapc5; rbbp8; tarbp1; irf3; uqcrc1; med23; ptprz1; myd88; ddb2; mdh1; mcm5; znf226; snrpd2; maf1; kifap3; ttll1; eaf2; dclre1a; wasf3; fau; c1orf66; prkcz; inhbb; mms19; nfib; elp3; sertad3; cd81; mettl1; mafb; gadd45a; ppm1a; msrb2; rps5; slc9a3r1; ctsl2; znf7; hipk2; usp11; clk3; ube2m; nedd8; mrpl23; supv3l1; lepre1; ppp2r1a; sirt2; hipk1; ripk2; lars2; slc2a4rg; suclg2; mcm4; prkx; oxa1l; lrpap1; fbxl5; ptpn14; sumo3; pola2; zbtb16; rpa1; fars2; rps27l; ndufs4; cbll1; eif2s3; prkaa1; med4; slbp; rnpep; mrpl49; ptprm; bag5; cln5; eya1; ruvbl2; hsbp1; hells; mipep; ncor1; akt3; pmpcb; znf230; hk1; c1orf25; chst1; irak1; msh2; usp3; phf21a; irf1; pcsk1; ints9; smyd3; ift88; znf304; uspl1; lars; cpd; zscan22; znf350; ptpru; cnot7; xrcc5; gbe1; eif3k; lta4h; rcor3; chd1; qars; dars2; prdx4; bmi1; sox15; supt16h; rab13; nfil3; apeh; yaf2; tyw1; march3; eef1b2; hoxa4; znf34; ccdc44; dcp1a; tars2; loc440434; trim5; atr; dnajc1; znf623; sars; mphosph8; gtf2f2; znf395; mark4; casp8ap2; mcm2; adnp2; sma4; anapc1; ubr5; gli3; fen1; rbmx; cops5; ccnb1ip1; eif4h; chfr; crnkl1; rnf7; ccnh; cstf3; znf323; gtf2h2; lgtn; ppp1r3d; manba; mtif2; atg7; hist2h2be; znf250; mtrf1; mcm3; proc; sall2; parp4; rbck1; aph1a; prmt8; erf; mrpl22; uimc1; znf394; mrpl34; kiaa0100; lrp8; plod1; ptk6; idh3b; ptpn13; znf331; nolc1; mybl1; amz2; znf16; znf223; atpif1; prkg2; mis12; mphosph10; c3; znf211; mme; rpl29; osgep; mid1; cda; rps16; p4hb; sltm; pygb; ubac1; rnf128; prmt5; cct6b; c17orf81; ryk; sox12; pcaf; ppap2a; blmh; mrpl15; mpdu1; tp53bp1; aldh2; mmp14; psmd8; dis3; dhx15; gcs1; idh3a; prkacb; phf20; tpr; eif3h; znf232; iars; hoxa5; rpp14; znf177; ctsk; hdac5; btg2; snrpb2; psip1; scand1; pabpc3; mkks; det1; tubd1; znf264; cdc7; tcf4; zxda; hsf1; mynn; dnmt1; parp1; cask; ranbp9; dpm1; trps1; thoc1; calr; htra1; wdhd1; cux2; apex1; ermp1; acad8; thrap3; mgea5; oxsm; mcm7; jak2; pdk4; pnkp; gys1; sec11a; cuedc2; csnk2a1; alg5; elf1; exosc4; zbtb10; tsta3; cdc25b; prdm4; ugcgl1; st6galnac2; smc1a; meis3p1; dtl; trim28; pet112l; trim22; nr1h3; rcbtb1; ctnnbip1; baz1a; thg1l; yeats4; mrpl33; acly; polr2i; bckdha; fancc; gmfb; nbn; rsl1d1; bcs1l; dapk1; agl; gins2; taf2; chd8; xpo7; foxc2; lnpep; hspbp1; ephb3; nars; hspa4l; atm; klf9 |
| GO:0005634 | 310 | 2330 | 0.00026 | nucleus; | trex1; rad17; mcm6; rb1; cpsf3l; ufm1; ctnnd1; supt7l; znf552; bhlhb3; cic; s100a6; irx5; puf60; gemin8; u1snrnpbp; pcnp; thyn1; znf432; gins1; rad51c; hnrnpa2b1; sertad2; notch1; eif6; poln; top2b; mizf; rad54b; itgb3bp; tsen34; mafg; dctn4; ung; pola1; ube3c; ppwd1; znf32; aff4; bccip; ddx24; ints8; ikzf5; asxl1; fanci; nup88; smc2; irx4; rbm23; gtf3c3; ranbp6; txnl4b; sall1; ctnnbl1; znf141; esf1; sox9; rragd; rfc1; znf473; sirt1; gtf2ird1; mitf; hoxa2; phb2; twist1; arid5b; phf17; nosip; ankrd28; prmt1; eapp; isoc2; cnot6; igfbp3; anapc5; polr1d; rbbp8; tarbp1; irf3; med23; ddb2; znf226; mcm5; serpina3; snrpd2; maf1; nxt2; nxf1; eaf2; dclre1a; fgf13; mms19; dhx32; sertad3; nfib; elp3; hax1; mafb; zfr; gadd45a; mtus1; card8; znf7; hipk2; ifih1; sept2; usp11; clk3; nedd8; ppp2r1a; supv3l1; sirt2; hipk1; mcm4; slc2a4rg; c11orf17; rcl1; cbs; ncl; pola2; zbtb16; rpa1; med4; slbp; ruvbl2; eya1; ssbp2; hsbp1; hells; strn3; ncor1; znf230; snupn; fam32a; msh2; phf21a; irf1; ints9; smyd3; znf304; c13orf15; sdcbp; cpd; zscan22; znf350; flna; xrcc5; cnot7; eif3k; chd1; rcor3; supt16h; sox15; bmi1; nfil3; yaf2; ncapg2; znf34; hoxa4; znf185; utp20; dcp1a; lmnb1; dnajc1; znf623; atr; mphosph8; gtf2f2; znf395; casp8ap2; mcm2; lgals7; adnp2; gli3; ubr5; tfpt; fen1; rbmx; cops5; ccnb1ip1; chfr; irs1; ccnh; rnf7; crnkl1; narfl; cstf3; znf323; gtf2h2; lgtn; znf250; hist2h2be; mcm3; sall2; parp4; znf394; uimc1; erf; erbb2ip; ptk6; ylpm1; znf331; nolc1; mybl1; znf16; znf223; srp54; mis12; parp16; mphosph10; znf211; ebna1bp2; osgep; bex1; sltm; kptn; gltscr2; gcom1; sh3bp4; sox12; tinp1; pcaf; blmh; tp53bp1; dis3; dhx15; prkacb; las1l; phf20; tpr; znf232; itpkc; arl4d; hoxa5; znf177; rpp14; hdac5; snrpb2; zwint; psip1; scand1; pnma1; det1; znf264; tubd1; cdc7; tcf4; zxda; hsf1; ihpk2; mynn; parp1; dnmt1; btaf1; mina; nvl; cask; ranbp9; trps1; thoc1; wdhd1; selenbp1; rhob; dcun1d4; cux2; cep57; apex1; mcm7; mgea5; thrap3; cfl1; pnkp; cuedc2; csnk2a1; exosc4; ccng1; elf1; spag7; zbtb10; prdm4; epn3; smc1a; trim28; dtl; meis3p1; trim22; nr1h3; rcbtb1; ctnnbip1; baz1a; yeats4; polr2i; fancc; znf665; maea; nbn; rsl1d1; isg20l2; arl4a; ttc35; gins2; taf2; chd8; xpo7; foxc2; s100p; hspa4l; atm; klf9 |
| GO:0045333 | 12 | 24 | 0.000262 | cellular respiration; | kiaa0100; oxa1l; sucla2; idh3b; sdha; mdh1; suclg1; idh3a; suclg2; uqcrc1; ndufs4; ndufs1 |
| GO:0010467 | 234 | 1697 | 0.000512 | gene expression; | znf304; mcm6; lars; zscan22; znf350; flna; rb1; cnot7; eif3k; chd1; ctnnd1; rcor3; qars; dars2; supt7l; supt16h; sox15; bmi1; znf552; nfil3; rab13; bhlhb3; tyw1; yaf2; fryl; cic; irx5; puf60; eef1b2; mrpl48; gemin8; znf34; hoxa4; u1snrnpbp; rps9; znf432; tars2; nmi; sertad2; hnrnpa2b1; znf623; sars; notch1; eif6; znf395; gtf2f2; casp8ap2; mcm2; snx6; mizf; adnp2; rad54b; gli3; tsen34; itgb3bp; rbmx; cops5; mafg; eif4h; ccnh; crnkl1; narfl; cstf3; znf323; gtf2h2; etf1; mrps30; lgtn; mtif2; znf250; znf32; ppwd1; aff4; mtrf1; mcm3; ints8; sall2; aph1a; erf; ikzf5; mrpl22; uimc1; znf394; mrpl34; asxl1; ecd; znf331; nolc1; mybl1; znf16; irx4; znf223; rpl8; flj20628; mrpl44; rbm23; mphosph10; gtf3c3; txnl4b; znf211; sall1; rpl29; znf141; mrpl13; esf1; sox9; rps16; sltm; mrpl24; ascc3; rfc1; znf473; rnf128; prmt5; c17orf81; gtf2ird1; sirt1; mitf; hoxa2; sox12; pcaf; mrpl15; phb2; twist1; arid5b; tp53bp1; phf17; dis3; cnot6; dhx15; tarbp1; rbbp8; polr1d; irf3; med23; phf20; tpr; eif3h; znf232; iars; hoxa5; myd88; rpp14; znf177; mcm5; znf226; snrpd2; hdac5; btg2; snrpb2; maf1; psip1; scand1; eaf2; fau; c1orf66; inhbb; mms19; elp3; nfib; sertad3; znf264; mettl1; tcf4; zxda; mafb; hsf1; mynn; parp1; dnmt1; btaf1; rps5; trps1; thoc1; calr; znf7; wdhd1; cux2; hipk2; apex1; thrap3; acad8; oxsm; mcm7; mrpl23; ppp2r1a; sirt2; hipk1; ripk2; elf1; exosc4; zbtb10; lars2; slc2a4rg; mcm4; prdm4; meis3p1; trim28; pet112l; trim22; nr1h3; rcbtb1; ctnnbip1; baz1a; thg1l; yeats4; mrpl33; zbtb16; fars2; polr2i; rps27l; bckdha; eif2s3; med4; slbp; mrpl49; rsl1d1; ssbp2; eya1; ruvbl2; hsbp1; taf2; chd8; hells; foxc2; ncor1; znf230; c1orf25; nars; irak1; klf9; phf21a; irf1; ints9 |
| GO:0006281 | 34 | 153 | 0.000622 | DNA repair; | trex1; rad17; gtf2h2; pnkp; ung; pola1; xrcc5; rbbp8; bccip; parp4; gadd45g; smc1a; supt16h; fanci; ddb2; btg2; rpa1; fancc; dclre1a; mms19; nbn; rad51c; ruvbl2; gadd45a; parp1; atr; poln; rad54b; msh2; fen1; atm; apex1; ccnh; tp53bp1 |
| GO:0006259 | 72 | 425 | 0.00215 | DNA metabolic process; | trex1; rad17; mcm6; rb1; xrcc5; rbbp8; chd1; gadd45g; bmi1; sox15; supt16h; ddb2; mcm5; hdac5; btg2; ccdc44; dclre1a; mms19; gins1; nfib; rad51c; gadd45a; atr; dnmt1; parp1; mphosph8; poln; mcm2; top2b; rad54b; fen1; apex1; ccnh; mcm7; supv3l1; ppp2r1a; pnkp; gtf2h2; sirt2; ung; pola1; hist2h2be; mcm3; bccip; mcm4; parp4; prmt8; smc1a; dtl; smc2; fanci; rcbtb1; yeats4; pola2; rpa1; fancc; nbn; ruvbl2; gins2; chd8; hells; ncor1; rfc1; prmt5; sirt1; msh2; gin1; atm; pcaf; phf21a; smyd3; tp53bp1 |
| GO:0000166 | 159 | 1108 | 0.00215 | nucleotide binding; | rad17; epha4; pck2; mcm6; lars; arl5a; xrcc5; chd1; dld; qars; sephs2; dars2; slc27a2; hras; rab13; sucla2; tyw1; abca12; dync1li1; puf60; u1snrnpbp; kdr; ror1; opa1; tars2; suclg1; rad51c; hnrnpa2b1; nek7; sars; gtf2f2; mark4; top2b; mcm2; rbmx; eif4h; centg2; igf1r; mapk10; rbm41; mtif2; pola1; mcm3; ddx24; hhat; atp8b4; ptk6; kif15; atp9b; smc2; nolc1; srp54; rbm35a; vps11; ptpla; prkg2; rbm23; nadsyn1; abcc4; atp2a2; plk2; dkfzp564o0523; rab9a; csnk2a2; sltm; guf1; ascc3; cad; rragd; rfc1; fastkd3; lonp1; rap2a; cct6b; ryk; vrk3; abca6; sdha; dhx15; rab21; slc22a4; c15orf24; prkacb; tpr; iars; itpkc; arl4d; pfas; hyou1; naip; mcm5; snrpb2; gtpbp6; nxf1; abcd3; pabpc3; prkcz; ndufa10; dhx32; mkks; rbm7; tubd1; cdc7; gna12; btaf1; cask; nvl; ola1; aox1; rhob; hipk2; ifih1; sept2; slc27a3; clk3; thrap3; acad8; oxsm; mcm7; jak2; mrpl23; pdk4; supv3l1; pnkp; hipk1; rap1b; ripk2; csnk2a1; acadvl; lars2; suclg2; mcm4; prkx; smc1a; ncl; hspa4; acly; fars2; eif2s3; prkaa1; rab3b; arl4a; ruvbl2; bcs1l; dapk1; hells; chd8; atad2; nt5c2; akt3; hk1; ephb3; nars; slc27a5; hspa4l; irak1; msh2; acadsb; rasl11b |
| GO:0008094 | 12 | 30 | 0.00229 | DNA-dependent ATPase activity; | mcm6; rfc1; mcm2; mcm5; rad54b; rad51c; xrcc5; mcm3; chd1; ruvbl2; mcm4; mcm7 |
| GO:0016070 | 197 | 1432 | 0.00277 | RNA metabolic process; | znf304; mcm6; lars; zscan22; znf350; rb1; cnot7; chd1; ctnnd1; rcor3; qars; dars2; supt7l; bmi1; sox15; supt16h; znf552; nfil3; rab13; bhlhb3; tyw1; yaf2; fryl; cic; irx5; puf60; gemin8; znf34; hoxa4; u1snrnpbp; znf432; dcp1a; tars2; nmi; sertad2; hnrnpa2b1; znf623; sars; notch1; znf395; gtf2f2; casp8ap2; mcm2; mizf; adnp2; rad54b; gli3; ubr5; tsen34; itgb3bp; rbmx; cops5; mafg; ccnh; crnkl1; cstf3; gtf2h2; znf323; ppwd1; znf32; znf250; aff4; mcm3; ddx24; ints8; sall2; erf; ikzf5; uimc1; znf394; asxl1; ecd; znf331; nolc1; mybl1; znf16; irx4; znf223; flj20628; mrpl44; rbm23; mphosph10; gtf3c3; txnl4b; znf211; sall1; znf141; esf1; sox9; sltm; ascc3; rfc1; znf473; prmt5; c17orf81; gtf2ird1; sirt1; mitf; hoxa2; sox12; pcaf; phb2; twist1; arid5b; tp53bp1; phf17; dis3; cnot6; dhx15; tarbp1; rbbp8; irf3; med23; tpr; phf20; znf232; iars; hoxa5; myd88; znf177; rpp14; mcm5; znf226; hdac5; snrpd2; snrpb2; btg2; maf1; psip1; scand1; eaf2; pabpc3; c1orf66; mms19; elp3; nfib; sertad3; znf264; mettl1; tcf4; zxda; mafb; hsf1; mynn; parp1; dnmt1; trps1; thoc1; calr; znf7; wdhd1; cux2; hipk2; apex1; acad8; thrap3; mcm7; ppp2r1a; sirt2; hipk1; elf1; exosc4; zbtb10; lars2; slc2a4rg; mcm4; prdm4; trim28; meis3p1; trim22; nr1h3; rcbtb1; ctnnbip1; baz1a; thg1l; yeats4; zbtb16; fars2; polr2i; bckdha; med4; slbp; eya1; ruvbl2; hsbp1; taf2; chd8; hells; foxc2; ncor1; znf230; c1orf25; nars; klf9; phf21a; irf1; ints9 |
| GO:0019752 | 57 | 321 | 0.0028 | carboxylic acid metabolic process; | gnpat; ech1; pck2; ppp2r1a; lars; aldh18a1; acy1; crot; lias; igfbp3; acadvl; cpt2; lars2; lta4h; qars; mccc2; sephs2; dars2; tpr; cbs; slc27a2; iars; pfas; plod1; idh3b; mdh1; ahcy; got2; acly; fars2; bckdha; ndufs4; prkaa1; rnpep; tars2; cd81; cryl1; acaa2; gamt; phgdh; got1; hadhb; aldh1l1; alox15b; ugdh; sars; cad; arpp-19; nars; slc27a5; slc27a3; pts; acadsb; dhfr; acad8; fah; oxsm |
| GO:0044429 | 53 | 294 | 0.00323 | mitochondrial part; | pdk4; mrpl23; supv3l1; sdha; aldh18a1; etfb; oxct1; acadvl; cpox; cpt2; lars2; uqcrc1; dld; mccc2; dars2; mrpl34; cyc1; ndufb11; oxa1l; ndufc1; fdxr; chchd3; got2; tomm70a; fars2; bckdha; ndufs4; ndufa10; opa1; tars2; ndufs3; mrpl13; ndufa3; suclg1; ndufs2; bcs1l; bcl2l2; mipep; hadhb; slc25a38; ndufs1; pmpcb; slc25a6; lonp1; mpv17; hk1; cox6c; mrps22; slc27a3; acadsb; slc9a6; phb2; aldh2 |
| GO:0006974 | 37 | 185 | 0.00337 | response to DNA damage stimulus; | trex1; rad17; gtf2h2; pnkp; ung; pola1; xrcc5; rbbp8; bccip; parp4; gadd45g; smc1a; dtl; supt16h; fanci; ddb2; btg2; rpa1; fancc; dclre1a; mms19; nbn; rad51c; ruvbl2; gadd45a; parp1; atr; poln; rad54b; ubr5; msh2; fen1; atm; apex1; mcm7; ccnh; tp53bp1 |
| GO:0005840 | 27 | 121 | 0.00337 | ribosome; | mrpl23; fau; eif2s3; rpl29; mrps30; mrps27; mrpl49; mrpl13; rsl1d1; rps16; mrpl24; mrpl22; mrpl34; rps5; rpl8; mrps22; mrps31; mrpl44; mrfap1l1; mrpl48; mrpl33; apex1; mett11d1; mrpl15; rps27l; rps9; oxsm |
| GO:0006082 | 57 | 324 | 0.00337 | organic acid metabolic process; | gnpat; ech1; pck2; ppp2r1a; lars; aldh18a1; acy1; crot; lias; igfbp3; acadvl; cpt2; lars2; lta4h; qars; mccc2; sephs2; dars2; tpr; cbs; slc27a2; iars; pfas; plod1; idh3b; mdh1; ahcy; got2; acly; fars2; bckdha; ndufs4; prkaa1; rnpep; tars2; cd81; cryl1; acaa2; gamt; phgdh; got1; hadhb; aldh1l1; alox15b; ugdh; sars; cad; arpp-19; nars; slc27a5; slc27a3; pts; acadsb; dhfr; acad8; fah; oxsm |
| GO:0006399 | 17 | 64 | 0.00488 | tRNA metabolic process; | lars; tars2; mettl1; lars2; qars; dars2; tpr; sars; iars; rpp14; tyw1; c1orf25; nars; thg1l; flj20628; tsen34; fars2 |
| GO:0006261 | 16 | 62 | 0.012 | DNA-dependent DNA replication; | trex1; rad17; mcm6; ppp2r1a; rfc1; pnkp; nbn; mcm2; mcm5; pola1; msh2; mcm3; mcm4; rpa1; atr; mcm7 |
| GO:0009719 | 42 | 235 | 0.0201 | response to endogenous stimulus; | trex1; rad17; mmp14; gtf2h2; pnkp; ung; pola1; xrcc5; rbbp8; bccip; parp4; gadd45g; smc1a; dtl; supt16h; fanci; ddb2; btg2; rpa1; bckdha; fancc; dclre1a; mms19; nbn; rad51c; ruvbl2; gadd45a; parp1; atr; foxc2; poln; rad54b; gal; ubr5; msh2; fen1; atm; apex1; irs1; ccnh; tp53bp1; mcm7 |
| GO:0043233 | 81 | 530 | 0.0221 | organelle lumen; | znf350; rb1; oxct1; cnot7; dld; med23; dars2; mccc2; hyou1; fdxr; hdac5; pnma1; eaf2; utp20; ndufa10; opa1; mms19; tars2; elp3; tcf4; mafb; parp1; ndufs1; gtf2f2; calr; thoc1; top2b; wdhd1; hipk2; mrps22; gli3; thrap3; mcm7; crnkl1; pdk4; mrpl23; supv3l1; gtf2h2; sirt2; cfl1; etfb; pola1; cpox; exosc4; lars2; ddx24; ints8; ugcgl1; rcl1; mrpl34; ylpm1; nolc1; ncl; yeats4; got2; srp54; zbtb16; polr2i; rpa1; fars2; mphosph10; bckdha; gtf3c3; maea; med4; mrpl13; cln5; p4hb; ruvbl2; taf2; mipep; pmpcb; lonp1; fam32a; sirt1; acadsb; pcaf; phf21a; ints9; aldh2; tp53bp1 |
| GO:0031974 | 81 | 530 | 0.0221 | membrane-enclosed lumen; | znf350; rb1; oxct1; cnot7; dld; med23; dars2; mccc2; hyou1; fdxr; hdac5; pnma1; eaf2; utp20; ndufa10; opa1; mms19; tars2; elp3; tcf4; mafb; parp1; ndufs1; gtf2f2; calr; thoc1; top2b; wdhd1; hipk2; mrps22; gli3; thrap3; mcm7; crnkl1; pdk4; mrpl23; supv3l1; gtf2h2; sirt2; cfl1; etfb; pola1; cpox; exosc4; lars2; ddx24; ints8; ugcgl1; rcl1; mrpl34; ylpm1; nolc1; ncl; yeats4; got2; srp54; zbtb16; polr2i; rpa1; fars2; mphosph10; bckdha; gtf3c3; maea; med4; mrpl13; cln5; p4hb; ruvbl2; taf2; mipep; pmpcb; lonp1; fam32a; sirt1; acadsb; pcaf; phf21a; ints9; aldh2; tp53bp1 |
| GO:0006412 | 45 | 260 | 0.0271 | translation; | mrpl23; etf1; lars; mrps30; lgtn; mtif2; lars2; mtrf1; eif3k; qars; dars2; tpr; eif3h; mrpl22; mrpl34; iars; pet112l; myd88; rpl8; eef1b2; mrpl48; mrpl33; fars2; rps27l; rps9; fau; eif2s3; rpl29; inhbb; tars2; rsl1d1; mrpl49; mrpl13; rps16; mrpl24; sars; eif6; rps5; rnf128; nars; cops5; mrpl15; irf1; eif4h; oxsm |
| GO:0017076 | 136 | 980 | 0.0273 | purine nucleotide binding; | rad17; epha4; pck2; mcm6; lars; arl5a; xrcc5; chd1; dld; qars; sephs2; dars2; hras; rab13; sucla2; abca12; dync1li1; kdr; ror1; opa1; tars2; suclg1; rad51c; nek7; sars; gtf2f2; mcm2; top2b; mark4; centg2; igf1r; mapk10; mtif2; mcm3; ddx24; hhat; atp8b4; ptk6; kif15; atp9b; smc2; nolc1; srp54; vps11; ptpla; prkg2; nadsyn1; abcc4; atp2a2; plk2; rab9a; csnk2a2; guf1; ascc3; cad; rragd; rfc1; fastkd3; rap2a; lonp1; cct6b; ryk; vrk3; abca6; sdha; dhx15; rab21; slc22a4; c15orf24; prkacb; tpr; iars; itpkc; arl4d; pfas; hyou1; mcm5; gtpbp6; abcd3; prkcz; ndufa10; dhx32; mkks; tubd1; cdc7; gna12; btaf1; cask; nvl; ola1; aox1; rhob; hipk2; sept2; ifih1; clk3; thrap3; acad8; oxsm; mcm7; jak2; pdk4; supv3l1; pnkp; hipk1; ripk2; csnk2a1; rap1b; acadvl; lars2; suclg2; mcm4; prkx; smc1a; hspa4; acly; fars2; eif2s3; prkaa1; rab3b; arl4a; dapk1; bcs1l; ruvbl2; chd8; hells; atad2; akt3; ephb3; hk1; nars; irak1; hspa4l; msh2; acadsb; rasl11b |
| GO:0030529 | 43 | 246 | 0.0273 | ribonucleoprotein complex; | mrpl23; mrps30; mrps27; ppwd1; dhx15; parp4; mrpl22; mrpl34; rpl8; snrpd2; snrpb2; mrps31; mrpl44; srp54; puf60; mrpl33; mrpl48; gemin8; mett11d1; rps27l; utp20; mphosph10; u1snrnpbp; txnl4b; rps9; fau; eif2s3; rpl29; slbp; mrpl49; mrpl13; rsl1d1; hnrnpa2b1; rps16; mrpl24; rps5; mrps22; rbmx; mrfap1l1; apex1; mrpl15; crnkl1; oxsm |
| GO:0007049 | 63 | 399 | 0.0375 | cell cycle; | cul1; rad17; mcm6; sirt2; c13orf15; rb1; pola1; cetn3; pkd2; ccng1; anapc5; rbbp8; mcm3; bccip; cdc25b; erbb2ip; smc1a; smc2; fanci; rcbtb1; nolc1; fhit; yeats4; s100a6; ncapg2; zwint; mis12; rpa1; mtss1; txnl4b; avpi1; dclre1a; stard13; maea; pcnp; plk2; nbn; rbm7; cdc7; gadd45a; hells; strn3; atr; mtus1; alox15b; bin3; mphosph8; calr; mn1; mcm2; rhob; sh3bp4; prmt5; sept2; anapc1; msh2; atm; pcaf; irf1; chfr; trim13; ccnh; mcm7 |
| GO:0005746 | 12 | 42 | 0.0421 | mitochondrial inner membrane#mitochondrial respiratory chain; | oxa1l; ndufa10; sdha; ndufc1; ndufs3; ndufa3; ndufs2; bcs1l; uqcrc1; ndufs4; ndufs1; cyc1 |
| GO:0005759 | 23 | 112 | 0.0424 | mitochondrial lumen#mitochondrial matrix; | pdk4; mrpl23; supv3l1; ndufa10; etfb; tars2; mrpl13; oxct1; lars2; dld; mccc2; mipep; dars2; mrpl34; pmpcb; lonp1; mrps22; fdxr; got2; acadsb; fars2; bckdha; aldh2 |
| GO:0031980 | 23 | 112 | 0.0424 | mitochondrial lumen; | pdk4; mrpl23; supv3l1; ndufa10; etfb; tars2; mrpl13; oxct1; lars2; dld; mccc2; mipep; dars2; mrpl34; pmpcb; lonp1; mrps22; fdxr; got2; acadsb; fars2; bckdha; aldh2 |

(B) Significantly enriched GO groups in the T4 differential network.

| GO | Group count | Total count | Pvalue  (FDR) | GO as name | Genes |
| --- | --- | --- | --- | --- | --- |
| GO:0005525 | 40 | 190 | 0.000148 | GTP binding; | rhobtb1; tpx2; rap1b; arl5a; gpr109b; gna15; spag1; rab14; tuba4a; rhof; rala; hras; nolc1; mtg1; ehd1; sept10; rhog; arhgef5; rab27a; arf4; dnm1l; tubb6; rab32; gnl3; rab5a; gspt1; arf1; gna12; scg5; rac1; rab22a; tubal3; lsg1; rab8a; rit1; eif2b2; tubg1; gtpbp2; clp1; xab1 |
| GO:0043066 | 30 | 138 | 0.000636 | negative regulation of apoptosis; | glo1; cfl1; mif; dffa; cdh13; nfkb1; tegt; anxa1; krt18; ddah2; hsp90b1; rb1cc1; sphk1; anxa5; socs2; casp3; serpinb2; il1a; bcl10; bag1; csf2; gpx1; tpt1; txndc5; ier3; bnip3l; aven; gclm; bard1; gsk3b |
| GO:0043069 | 30 | 138 | 0.000636 | negative regulation of programmed cell death; | glo1; cfl1; mif; dffa; cdh13; nfkb1; tegt; anxa1; krt18; ddah2; hsp90b1; rb1cc1; sphk1; anxa5; socs2; casp3; serpinb2; il1a; bcl10; bag1; csf2; gpx1; tpt1; txndc5; ier3; bnip3l; aven; gclm; bard1; gsk3b |
| GO:0000278 | 35 | 177 | 0.00138 | mitotic cell cycle; | tpx2; foxo4; spc25; spag5; c18orf24; sphk1; fosl1; plk1; ccnb1; nolc1; cdk2ap1; cenpe; nde1; akap8; mad1l1; mphosph6; ncaph; map2k1; smad3; smc4; dbf4; gspt1; ccna2; cdca8; kif23; ereg; kif2c; aurkb; fbxo5; mad2l1; inhba; ccne1; ruvbl1; ncapg; nedd9 |
| GO:0000087 | 27 | 127 | 0.00289 | M phase of mitotic cell cycle; | ncaph; map2k1; tpx2; spc25; smc4; spag5; c18orf24; ccna2; fosl1; cdca8; plk1; kif23; ccnb1; nolc1; kif2c; ereg; cenpe; aurkb; fbxo5; mad2l1; nde1; ruvbl1; akap8; ncapg; mphosph6; mad1l1; nedd9 |
| GO:0012501 | 72 | 468 | 0.00314 | programmed cell death; | cd14; sgpp1; fadd; traf6; nfkb1; adrb2; lgals1; chek2; tnfrsf12a; il1a; trib3; bcl10; mnt; egln3; stk17a; siah2; pecr; traf5; vdac1; smad3; anxa1; pdcd5; anxa5; casp3; serpinb2; tpt1; gpx1; bag1; ctnnal1; bnip3l; tp53i3; aven; actn1; tns4; gclm; glo1; prodh; cfl1; mif; gja1; cdh13; tegt; ddah2; sgk; rb1cc1; sphk1; csf2; nupr1; acin1; pak1; bard1; cebpg; dnm1l; litaf; tlr2; hspe1; dffa; dusp22; ppp1r15a; krt18; hsp90b1; glrx2; cib1; gspt1; socs2; top1; zak; txndc5; ier3; inhba; rp6-213h19.1; gsk3b |
| GO:0006915 | 71 | 465 | 0.00465 | apoptosis; | cd14; sgpp1; fadd; traf6; nfkb1; adrb2; lgals1; chek2; tnfrsf12a; il1a; trib3; bcl10; mnt; egln3; stk17a; siah2; pecr; traf5; vdac1; smad3; anxa1; pdcd5; anxa5; casp3; serpinb2; tpt1; gpx1; bag1; ctnnal1; bnip3l; tp53i3; aven; actn1; tns4; gclm; glo1; prodh; cfl1; mif; gja1; cdh13; tegt; ddah2; sgk; rb1cc1; sphk1; csf2; nupr1; acin1; pak1; bard1; cebpg; dnm1l; litaf; tlr2; hspe1; dffa; dusp22; ppp1r15a; krt18; hsp90b1; glrx2; cib1; gspt1; socs2; zak; txndc5; ier3; inhba; rp6-213h19.1; gsk3b |
| GO:0042981 | 52 | 314 | 0.00467 | regulation of apoptosis; | glo1; prodh; cfl1; mif; fadd; cdh13; traf6; nfkb1; tegt; ddah2; adrb2; rb1cc1; lgals1; sphk1; chek2; il1a; bcl10; csf2; mnt; nupr1; stk17a; pecr; bard1; cebpg; traf5; hspe1; tlr2; dffa; smad3; krt18; anxa1; hsp90b1; pdcd5; gspt1; anxa5; socs2; casp3; serpinb2; bag1; gpx1; tpt1; zak; txndc5; ier3; bnip3l; tp53i3; inhba; aven; actn1; gclm; rp6-213h19.1; gsk3b |
| GO:0032555 | 125 | 934 | 0.00467 | purine ribonucleotide binding; | sacs; rhobtb1; golga5; dyrk3; ddx39; ttf2; plk4; arl5a; gna15; cdkl3; tuba4a; cars; rab14; chek2; rage; hras; trib3; uckl1; mtg1; stk17a; epha2; arhgef5; mvd; map2k1; map4k4; fksg30; tor1b; gnl3; hspa5; pfkp; smc4; rab5a; nme4; arf1; gna12; csnk1e; atp2b1; bckdk; mccc1; tubal3; lsg1; rab8a; bat1; tgfbr1; atp13a3; csnk1g2; vrk2; mknk2; oxsr1; met; tk1; tpx2; rap1b; ephb6; myo19; gpr109b; ilk; sgk; spag1; sphk1; rhof; rala; pfkfb3; rfk; plk1; gne; bmpr2; nolc1; gars; ehd1; sept10; cenpe; loc146909; rhog; rab27a; melk; abl2; pak1; arf4; dnm1l; pdk1; tubb6; kif18a; c9orf167; rab32; hspe1; stch; trip13; atpbd1b; hsp90b1; map2k4; ddx21; gspt1; plk3; kif14; rps6ka3; prkcd; scg5; ascc3; srpk1; p2rx4; pank2; rab22a; rac1; kif23; rit1; eif2b2; lonp1; kif2c; tubg1; gtpbp2; zak; aurkb; hspa14; uck2; clp1; gmps; tesk1; ruvbl1; pcolce2; xab1; stk39; abce1; rp6-213h19.1; gsk3b |
| GO:0032553 | 125 | 934 | 0.00467 | ribonucleotide binding; | sacs; rhobtb1; golga5; dyrk3; ddx39; ttf2; plk4; arl5a; gna15; cdkl3; tuba4a; cars; rab14; chek2; rage; hras; trib3; uckl1; mtg1; stk17a; epha2; arhgef5; mvd; map2k1; map4k4; fksg30; tor1b; gnl3; hspa5; pfkp; smc4; rab5a; nme4; arf1; gna12; csnk1e; atp2b1; bckdk; mccc1; tubal3; lsg1; rab8a; bat1; tgfbr1; atp13a3; csnk1g2; vrk2; mknk2; oxsr1; met; tk1; tpx2; rap1b; ephb6; myo19; gpr109b; ilk; sgk; spag1; sphk1; rhof; rala; pfkfb3; rfk; plk1; gne; bmpr2; nolc1; gars; ehd1; sept10; cenpe; loc146909; rhog; rab27a; melk; abl2; pak1; arf4; dnm1l; pdk1; tubb6; kif18a; c9orf167; rab32; hspe1; stch; trip13; atpbd1b; hsp90b1; map2k4; ddx21; gspt1; plk3; kif14; rps6ka3; prkcd; scg5; ascc3; srpk1; p2rx4; pank2; rab22a; rac1; kif23; rit1; eif2b2; lonp1; kif2c; tubg1; gtpbp2; zak; aurkb; hspa14; uck2; clp1; gmps; tesk1; ruvbl1; pcolce2; xab1; stk39; abce1; rp6-213h19.1; gsk3b |
| GO:0007242 | 108 | 784 | 0.00467 | intracellular signaling cascade; | cap2; rhobtb1; fadd; traf6; arl5a; ifnar2; gna15; adrb2; slc20a1; rab14; lgals1; fhl2; wsb2; hras; arhgef18; golt1b; bcl10; tgfb1i1; depdc1; ecm1; stk17a; arhgef5; siah2; cxcl1; mc1r; traf5; vdac1; map4k4; ppan; smad3; trem1; rab5a; arf1; casp3; vapa; mt2a; rab8a; gpx1; pthlh; ect2; def8; ticam1; ccne1; mknk2; dusp2; oxsr1; met; cfl1; gja1; cdh13; rap1b; cyp26b1; spag5; ddah2; rb1cc1; sphk1; rhof; rala; adipor2; arhgef2; arhgef3; pik3r1; arhgdib; adam9; ctgf; rhog; carhsp1; rab27a; arfgap3; tlr6; acin1; pak1; arf4; dnm1l; pdk1; centd3; sh2b3; litaf; rab32; rbm14; hspe1; dffa; dusp22; smap1; hrb; plek2; ddef2; map2k4; gspt1; dvl1; socs2; rasa2; prkcd; srpk1; atp6ap2; rab22a; rac1; tmepai; rit1; farp1; bcar3; zak; zfp36; klf9; rgs19; fgd6; xab1; gsk3b |
| GO:0043067 | 52 | 316 | 0.00467 | programmed cell death#regulation of programmed cell death; | glo1; prodh; cfl1; mif; fadd; cdh13; traf6; nfkb1; tegt; ddah2; adrb2; rb1cc1; lgals1; sphk1; chek2; il1a; bcl10; csf2; mnt; nupr1; stk17a; pecr; bard1; cebpg; traf5; hspe1; tlr2; dffa; smad3; krt18; anxa1; hsp90b1; pdcd5; gspt1; anxa5; socs2; casp3; serpinb2; bag1; gpx1; tpt1; zak; txndc5; ier3; bnip3l; tp53i3; inhba; aven; actn1; gclm; rp6-213h19.1; gsk3b |
| GO:0007067 | 26 | 125 | 0.00467 | mitosis; | ncaph; map2k1; tpx2; spc25; smc4; spag5; c18orf24; ccna2; fosl1; cdca8; plk1; kif23; ccnb1; nolc1; kif2c; ereg; cenpe; aurkb; fbxo5; mad2l1; nde1; ruvbl1; akap8; ncapg; mad1l1; nedd9 |
| GO:0022403 | 35 | 189 | 0.00532 | cell cycle phase; | tpx2; foxo4; spc25; spag5; cyp26b1; c18orf24; fosl1; sc65; plk1; ccnb1; nolc1; cdk2ap1; cenpe; nde1; akap8; mad1l1; mphosph6; ncaph; map2k1; smc4; dbf4; gspt1; ccna2; cdca8; kif23; ereg; kif2c; aurkb; fbxo5; mad2l1; inhba; ccne1; ruvbl1; ncapg; nedd9 |
| GO:0008219 | 73 | 491 | 0.00647 | cell death; | cd14; sgpp1; fadd; traf6; nfkb1; adrb2; lgals1; chek2; tnfrsf12a; il1a; trib3; bcl10; mnt; egln3; stk17a; siah2; pecr; traf5; vdac1; smad3; anxa1; pdcd5; anxa5; casp3; serpinb2; tpt1; gpx1; bag1; ctnnal1; bnip3l; tp53i3; aven; actn1; tns4; gclm; glo1; mmd; prodh; cfl1; mif; gja1; cdh13; tegt; ddah2; sgk; rb1cc1; sphk1; csf2; nupr1; acin1; pak1; bard1; cebpg; dnm1l; litaf; tlr2; hspe1; dffa; dusp22; ppp1r15a; krt18; hsp90b1; glrx2; cib1; gspt1; socs2; top1; zak; txndc5; ier3; inhba; rp6-213h19.1; gsk3b |
| GO:0016265 | 73 | 491 | 0.00647 | death; | cd14; sgpp1; fadd; traf6; nfkb1; adrb2; lgals1; chek2; tnfrsf12a; il1a; trib3; bcl10; mnt; egln3; stk17a; siah2; pecr; traf5; vdac1; smad3; anxa1; pdcd5; anxa5; casp3; serpinb2; tpt1; gpx1; bag1; ctnnal1; bnip3l; tp53i3; aven; actn1; tns4; gclm; glo1; mmd; prodh; cfl1; mif; gja1; cdh13; tegt; ddah2; sgk; rb1cc1; sphk1; csf2; nupr1; acin1; pak1; bard1; cebpg; dnm1l; litaf; tlr2; hspe1; dffa; dusp22; ppp1r15a; krt18; hsp90b1; glrx2; cib1; gspt1; socs2; top1; zak; txndc5; ier3; inhba; rp6-213h19.1; gsk3b |
| GO:0017076 | 129 | 980 | 0.00669 | purine nucleotide binding; | sacs; golga5; ddx39; ttf2; plk4; arl5a; gna15; cars; tuba4a; chek2; hras; stk17a; epha2; map2k1; map4k4; tor1b; gnl3; hspa5; smc4; nme4; arf1; csnk1e; atp2b1; steap1; mccc1; lsg1; tubal3; vrk2; mknk2; tk1; myo19; gpr109b; sgk; fmo4; spag1; rhof; pfkfb3; rfk; gne; nolc1; gars; sept10; cenpe; pak1; abl2; dnm1l; atpbd1b; trip13; map2k4; gspt1; rps6ka3; ascc3; scg5; prkcd; pank2; lonp1; tubg1; hspa14; clp1; uck2; tesk1; ruvbl1; stk39; rp6-213h19.1; abce1; gsk3b; rhobtb1; dyrk3; cdkl3; rab14; rage; trib3; uckl1; mtg1; xdh; arhgef5; mvd; fksg30; pfkp; rab5a; gna12; bckdk; rab8a; bat1; tgfbr1; atp13a3; csnk1g2; met; oxsr1; tpx2; ephb6; rap1b; ilk; sphk1; rala; plk1; bmpr2; ehd1; loc146909; rhog; rab27a; melk; arf4; pdk1; tubb6; kif18a; c9orf167; rab32; hspe1; stch; hsp90b1; ddx21; plk3; kif14; srpk1; p2rx4; rab22a; rac1; kif23; rit1; eif2b2; kif2c; gtpbp2; ero1l; zak; aurkb; gmps; xab1; pcolce2 |
| GO:0000166 | 143 | 1108 | 0.0068 | nucleotide binding; | sacs; golga5; ddx39; ttf2; plk4; arl5a; gna15; cars; tuba4a; chek2; hras; pprc1; stk17a; epha2; dazap1; map2k1; map4k4; tor1b; gnl3; hspa5; smc4; hnrnpa2b1; nme4; arf1; csnk1e; parn; atp2b1; steap1; mccc1; lsg1; tubal3; vrk2; mknk2; mbd1; tk1; myo19; gpr109b; sgk; fmo4; spag1; rhof; pfkfb3; igf2bp2; rfk; gne; nolc1; gars; sept10; cenpe; acin1; sfrs9; pak1; abl2; dnm1l; rbm14; rdbp; atpbd1b; trip13; map2k4; gspt1; rps6ka3; ascc3; scg5; prkcd; pank2; lonp1; tubg1; hspa14; uck2; clp1; tesk1; ruvbl1; stk39; abce1; rp6-213h19.1; gsk3b; rhobtb1; dyrk3; cdkl3; rab14; rage; uckl1; trib3; mtg1; xdh; arhgef5; mvd; fksg30; pfkp; rab5a; gna12; bckdk; rab8a; bat1; slc27a3; skil; tgfbr1; atp13a3; csnk1g2; oxsr1; met; tpx2; rap1b; ephb6; ilk; sphk1; rala; plk1; bmpr2; ehd1; loc146909; rhog; rab27a; melk; arf4; pdk1; tubb6; hspe1; kif18a; c9orf167; rab32; stch; hsp90b1; ddx21; plk3; kif14; srpk1; p2rx4; rab22a; rac1; kif23; rit1; eif2b2; kif2c; gtpbp2; ero1l; zak; aurkb; gmps; c14orf156; pygl; xab1; pcolce2 |
| GO:0046907 | 57 | 365 | 0.00921 | intracellular transport; | stx2; sec23a; cope; golga5; ankrd27; homer3; ddx39; ergic2; nup153; adrb2; tuba4a; rab14; hmg2l1; npc1; xpo6; slc25a4; ap2b1; nup93; ykt6; arcn1; ehd1; nxt1; cenpe; loc146909; ppih; arfgap3; tom1l1; bard1; ap1m2; lman1; slc25a37; tubb6; mxi1; kif18a; nfkbie; tomm34; hrb; ap4m1; krt18; smad3; sec61a1; cspg5; bet1; sec24a; kif14; scg5; tubal3; kif23; tmem48; napa; vps41; bat1; uxt; kif2c; sec61g; sec31a; gsk3b |
| GO:0000279 | 29 | 152 | 0.0108 | M phase; | ncaph; map2k1; tpx2; spc25; smc4; spag5; cyp26b1; c18orf24; ccna2; fosl1; cdca8; sc65; plk1; kif23; ccnb1; nolc1; kif2c; ereg; cenpe; aurkb; fbxo5; mad2l1; nde1; ruvbl1; akap8; ncapg; mphosph6; mad1l1; nedd9 |
| GO:0005819 | 13 | 44 | 0.0164 | spindle; | kif23; tpx2; tubg1; ndel1; spag5; cenpe; aurkb; c18orf24; fbxo5; nde1; mad1l1; ptp4a1; nedd9 |
| GO:0043123 | 14 | 56 | 0.0198 | positive regulation of I-kappaB kinase/NF-kappaB cascade; | traf5; golt1b; litaf; bcl10; fadd; gja1; traf6; ect2; slc20a1; lgals1; ecm1; ticam1; tlr6; vapa |
| GO:0001726 | 9 | 24 | 0.0206 | leading edge#ruffle; | centd3; mtmr14; pdpn; s100a11; rab5a; cyfip1; fgd6; itga5; tnfrsf12a |
| GO:0009064 | 10 | 29 | 0.0206 | glutamine family amino acid metabolic process; | prodh; ctps; asns; arg2; ddah2; ggh; gfpt1; gmps; asl; gclm |
| GO:0022402 | 50 | 322 | 0.0253 | cell cycle process; | tpx2; foxo4; spc25; spag5; cyp26b1; rb1cc1; rbbp8; sphk1; c18orf24; chek2; fosl1; myc; sc65; plk1; ccnb1; bcl10; nolc1; mcc; cenpe; cdk2ap1; nde1; akap8; mphosph6; bard1; mad1l1; ncaph; map2k1; ppp1r15a; smad3; smc4; dbf4; mnat1; gspt1; ccna2; cdca8; kif23; kif2c; ereg; tubg1; zak; aurkb; fbxo5; mad2l1; ccne1; bmp2; ruvbl1; inhba; cks1b; ncapg; nedd9 |
| GO:0006916 | 20 | 97 | 0.0318 | anti-apoptosis; | glo1; cfl1; cdh13; nfkb1; anxa1; ddah2; hsp90b1; sphk1; socs2; anxa5; serpinb2; il1a; tpt1; gpx1; bag1; csf2; txndc5; ier3; bnip3l; aven |
| GO:0048193 | 12 | 42 | 0.0319 | Golgi vesicle transport; | lman1; sec23a; cope; golga5; napa; ergic2; krt18; sec31a; rab14; bet1; sec24a; ykt6 |
| GO:0043122 | 14 | 59 | 0.0371 | regulation of I-kappaB kinase/NF-kappaB cascade; | traf5; golt1b; litaf; bcl10; fadd; gja1; traf6; ect2; slc20a1; lgals1; ecm1; ticam1; tlr6; vapa |
| GO:0051301 | 23 | 120 | 0.0413 | cell division; | ncaph; stx2; spc25; smc4; spag5; c18orf24; ccna2; ppp1ca; cdca8; ccnb1; kif23; sept10; cenpe; aurkb; fbxo5; nde1; mad2l1; ccne1; ruvbl1; cks1b; ncapg; mad1l1; nedd9 |
| GO:0006888 | 7 | 17 | 0.0428 | ER to Golgi vesicle-mediated transport; | sec31a; lman1; sec23a; bet1; sec24a; ergic2; ykt6 |

(C) Significantly enriched GO groups in the EGFR/ITGB1-T4R differential network.

| GO | Group count | Total count | Pvalue  (FDR) | GO as name | Genes |
| --- | --- | --- | --- | --- | --- |
| GO:0004128 | 5 | 6 | 0.0268 | cytochrome-b5 reductase activity; | cyb561; cyb5r4; nqo1; cyb5r3; cyb5r1 |
| GO:0016653 | 5 | 7 | 0.0331 | oxidoreductase activity, acting on NADH or NADPH, heme protein as acceptor; | cyb561; cyb5r4; nqo1; cyb5r3; cyb5r1 |
| GO:0005911 | 16 | 83 | 0.0331 | intercellular junction; | shroom2; cdc42bpb; dsp; dsc2; synpo; dsg3; cldn1; jup; amotl2; dlg1; tjp1; tjp2; dsc3; dsc1; pard3; gjb5 |

(D) Significantly enriched GO groups in the PI3K/MAPKK-T4R differential network.

| GO | Group count | Total count | Pvalue  (FDR) | GO as name | Genes |
| --- | --- | --- | --- | --- | --- |
| GO:0005764 | 25 | 110 | 0.00532 | lysosome; | gaa; trip10; cd63; mcoln1; march2; ppt1; hyal1; fuca1; dram; ifi30; sftpd; usp6; abca5; racgap1; gns; cln3; plekhf1; man2b1; lamp3; fnbp1; psap; vps11; acp2; lamp1; ctsa |
| GO:0000323 | 25 | 110 | 0.00532 | lytic vacuole; | gaa; trip10; cd63; mcoln1; march2; ppt1; hyal1; fuca1; dram; ifi30; sftpd; usp6; abca5; racgap1; gns; cln3; plekhf1; man2b1; lamp3; fnbp1; psap; vps11; acp2; lamp1; ctsa |
| GO:0016568 | 24 | 105 | 0.0058 | chromatin modification; | morf4l1; suv420h1; cpa4; setd2; cbx7; hira; satb1; ep300; actl6a; setmar; jmjd1a; ep400; suv39h1; huwe1; hdac5; hdac9; hdac2; yeats4; hmg20a; bnip3; bptf; nsd1; cabin1; bcor |
| GO:0005773 | 26 | 121 | 0.01 | vacuole; | gaa; trip10; cd63; atp6ap1; mcoln1; march2; ppt1; hyal1; fuca1; dram; ifi30; sftpd; usp6; abca5; racgap1; gns; cln3; plekhf1; man2b1; lamp3; fnbp1; psap; vps11; acp2; lamp1; ctsa |
| GO:0043687 | 105 | 758 | 0.0247 | post-translational protein modification; | tpst1; mmp14; icmt; ube2l6; nek4; tulp4; ncstn; brcc3; tsg101; satb1; ep300; rnf19b; prkra; rage; clk2; usp34; huwe1; evpl; hdac9; hdac2; hipk3; rps6kc1; kdr; ccl2; ccnd3; fbxl14; pim2; tyk2; rps6ka1; cpa4; herc1; cyld; fbxo2; ptp4a2; ptk2b; ube2v2; pias1; bckdk; col4a3bp; asb9; rbbp6; anapc1; dyrk4; usp33; mknk1; csnk1g3; snrk; nsd1; map3k5; tceb2; chuk; mknk2; usp25; wdr5b; hipk1; ulk1; fbxl2; fbxo3; ube3c; ephb6; rnf125; march2; ilk; dda1; sgk; anapc2; rnf11; aph1a; usp6; ripk4; hectd3; nid1; ptk6; spcs1; pak2; bap1; c4orf20; tpst2; prkg2; prkci; clk1; pdk1; abl1; jak1; plk2; trpm6; mylip; eif2ak2; mid1; isg15; map2k4; mast4; ube2e1; march8; map3k8; irak1; sgk3; tyro3; tnni3k; ptpre; usp3; klhl21; ppm1h; tnf; cit |
| GO:0006325 | 30 | 158 | 0.0389 | establishment and/or maintenance of chromatin architecture; | morf4l1; suv420h1; cpa4; setd2; cbx7; hira; arid4a; satb1; ep300; actl6a; setmar; jmjd1a; sox15; ep400; suv39h1; hmgb2; hdac5; huwe1; hdac9; hdac2; yeats4; hist1h2bc; h1fx; hmg20a; bnip3; bptf; nsd1; h1f0; cabin1; bcor |

(E) Significantly enriched GO groups in the MMP-T4R differential network.

| GO | Group count | Total count | Pvalue  (FDR) | GO as name | Genes |
| --- | --- | --- | --- | --- | --- |
| GO:0000502 | 12 | 35 | 2.41E-05 | proteasome complex (sensu Eukaryota); | psme3; psma4; psmb8; psmd10; psma3; psmb9; psme2; psmd7; psma6; psmc2; psma2; psmd6 |
| GO:0043285 | 26 | 190 | 8.26E-05 | biopolymer catabolic process; | uspl1; dcp2; dcps; psmb9; pepd; chi3l2; gja1; ufd1l; anapc5; phkb; ppt1; psmc2; cycs; psma2; tprkb; psma4; psmb8; psma3; loc220594; usp18; ovgp1; usp34; cln3; psma6; zfp36; abce1 |
| GO:0016491 | 45 | 423 | 0.000172 | oxidoreductase activity; | aldh9a1; bdh1; ndufa9; srd5a3; decr2; rrm1; ndufa6; cycs; pnpo; decr1; f8; sepx1; ndufa13; pgd; ndufc2; mical3; cbr1; cyp4f2; mosc1; blvrb; cyp4f12; fxn; idh1; ndufa4; idh2; ndufa8; ndufs7; fads3; nqo1; hsd17b4; akr1c3; ifi30; hadh; spr; akr1a1; ptges2; cyp4f11; bdh2; uqcr; dhfr; uqcrq; uevld; abce1; uqcrfs1; scp2 |
| GO:0006457 | 16 | 99 | 0.000495 | protein folding; | hspe1; sep15; tor3a; dnajc7; nktr; pigk; calr; cln3; bag2; clpx; dnajc12; dnajb4; lman2l; ppih; vbp1; pin1 |
| GO:0009057 | 29 | 244 | 0.000835 | macromolecule catabolic process; | uspl1; dcp2; dcps; psmb9; pepd; chi3l2; gja1; ufd1l; anapc5; phkb; ppt1; psmc2; cycs; psma2; tprkb; psma4; psmb8; psma3; pgd; loc220594; usp18; ovgp1; gns; usp34; cln3; psma6; zfp36; uevld; abce1 |
| GO:0005746 | 11 | 42 | 0.000835 | mitochondrial inner membrane #mitochondrial respiratory chain; | ndufa13; ndufc2; ndufa9; ndufa4; ndufa8; ndufs7; uqcr; uqcrq; ndufa6; cycs; uqcrfs1 |
| GO:0005839 | 6 | 13 | 0.00203 | proteasome core complex (sensu Eukaryota); | psma4; psmb8; psma6; psma3; psmb9; psma2 |
| GO:0006091 | 33 | 308 | 0.00258 | generation of precursor metabolites and energy; | aldh9a1; ndufa9; ndufa6; cycs; fech; pgd; ndufc2; mical3; cyp4f2; cyp4f12; atp5g1; acaa1; atp6v0d1; fxn; idh1; ndufa4; idh2; phkb; atp6v1e1; aco1; ndufs7; fads3; ndufa8; nqo1; akr1c3; spr; ptges2; cyp4f11; uqcr; uqcrq; echs1; abce1; uqcrfs1 |
| GO:0044248 | 33 | 309 | 0.00273 | cellular catabolic process; | uspl1; dcps; psmb9; ephx2; anapc5; ppt1; cycs; psma2; aspa; psma4; psmb8; loc220594; pgd; ovgp1; usp18; usp34; psma6; dera; dcp2; chi3l2; ufd1l; idh1; idh2; phkb; aco1; pccb; psma3; gns; pla2g4a; akr1a1; zfp36; uevld; abce1 |
| GO:0009056 | 39 | 388 | 0.00284 | catabolic process; | uspl1; dcps; psmb9; ephx2; gja1; anapc5; ppt1; psmc2; cycs; pafah1b3; psma2; aspa; psma4; tprkb; psmb8; pgd; loc220594; usp18; ovgp1; usp34; cln3; psma6; dera; dcp2; pepd; chi3l2; ufd1l; idh1; idh2; phkb; aco1; pccb; psma3; gns; pla2g4a; akr1a1; zfp36; uevld; abce1 |
| GO:0004298 | 6 | 14 | 0.00301 | threonine endopeptidase activity; | psma4; psmb8; psma6; psma3; psmb9; psma2 |
| GO:0032787 | 19 | 144 | 0.00349 | monocarboxylic acid metabolic process; | acaa1; aldh9a1; ephx2; idh2; idh1; fads3; hsd17b4; akr1c3; pccb; prkab1; hadh; pla2g4a; acot2; cyp4f2; akr1a1; ptges2; bdh2; echs1; acn9 |
| GO:0032991 | 95 | 1229 | 0.00526 | macromolecular complex; | pnn; psmb9; ttf2; aaas; anapc5; wdr3; brcc3; psmc2; cycs; timm8b; cog8; psma4; psmd10; srprb; ndufa13; tubgcp5; ddx5; fxc1; ppih; atp5g1; psme3; imp3; prim2; atp6v0d1; tomm22; hdac1; c16orf33; pop4; tubd1; npip; pcbp2; mrps33; ndufa4; kctd15; taf5; ash2l; polr2l; med7; psmd6; gnai1; nup160; vbp1; cog2; uqcrfs1; elp4; tsc1; timm13; psmd7; gja1; ndufa9; tap1; paics; rrm1; ndufa6; ap2b1; mrpl34; psma2; ddx41; pigk; psmb8; spcs1; ndufc2; rpl26l1; sf3b5; psme2; hla-dra; rpp40; eif2b1; psma6; taf15; ercc8; dynlt1; mrpl46; nupl2; rpa2; orc3l; kctd12; atp6v1e1; atp1b3; snrpc; ndufs7; ndufa8; taf11; mrps35; psma3; map2; prpsap2; qtrt1; eif2b3; atp5j2; mycbp; mrps18b; uqcrq; mrps11; mrps10 |
| GO:0030163 | 18 | 139 | 0.00753 | protein catabolic process; | uspl1; psmb9; pepd; gja1; ufd1l; anapc5; ppt1; psmc2; psma2; tprkb; psma4; psmb8; psma3; loc220594; usp18; usp34; cln3; psma6 |
| GO:0044265 | 22 | 187 | 0.00844 | cellular macromolecule catabolic process; | uspl1; dcp2; dcps; psmb9; chi3l2; ufd1l; anapc5; phkb; psma2; psma4; psmb8; psma3; loc220594; pgd; ovgp1; usp18; gns; usp34; psma6; zfp36; uevld; abce1 |
| GO:0016655 | 8 | 31 | 0.00844 | oxidoreductase activity, acting on NADH or NADPH, quinone or similar compound as acceptor; | ndufa13; ndufc2; ndufa9; ndufa4; ndufs7; ndufa8; ndufa6; nqo1 |
| GO:0016616 | 12 | 66 | 0.00844 | oxidoreductase activity, acting on the CH-OH group of donors, NAD or NADP as acceptor; | hadh; pgd; spr; bdh1; cbr1; idh1; akr1a1; idh2; bdh2; uevld; hsd17b4; akr1c3 |
| GO:0006119 | 10 | 51 | 0.0149 | oxidative phosphorylation; | atp6v0d1; ndufc2; ndufa9; ndufa4; atp6v1e1; ndufa8; ndufs7; uqcr; ndufa6; atp5g1 |
| GO:0016614 | 12 | 72 | 0.0176 | oxidoreductase activity, acting on CH-OH group of donors; | hadh; pgd; spr; bdh1; cbr1; idh1; akr1a1; idh2; bdh2; uevld; hsd17b4; akr1c3 |
| GO:0006118 | 23 | 208 | 0.0176 | electron transport; | aldh9a1; ndufa9; fxn; ndufa4; ndufa8; ndufs7; fads3; ndufa6; nqo1; cycs; akr1c3; spr; pgd; ndufc2; mical3; cyp4f2; ptges2; cyp4f11; uqcr; uqcrq; cyp4f12; abce1; uqcrfs1 |
| GO:0030964 | 7 | 27 | 0.0176 | NADH dehydrogenase complex (quinone); | ndufa4; ndufs7; ndufa13; ndufa8; ndufc2; ndufa6; ndufa9 |
| GO:0045271 | 7 | 27 | 0.0176 | respiratory chain complex I; | ndufa4; ndufs7; ndufa13; ndufa8; ndufc2; ndufa6; ndufa9 |
| GO:0005747 | 7 | 27 | 0.0176 | mitochondrial inner membrane#mitochondrial respiratory chain#mitochondrial respiratory chain complex I; | ndufa4; ndufs7; ndufa13; ndufa8; ndufc2; ndufa6; ndufa9 |
| GO:0006631 | 14 | 103 | 0.0179 | fatty acid metabolic process; | prkab1; acaa1; hadh; ephx2; pla2g4a; acot2; cyp4f2; ptges2; bdh2; fads3; echs1; hsd17b4; pccb; akr1c3 |
| GO:0043681 | 4 | 8 | 0.0187 | protein import into mitochondrion; | fxc1; tomm22; timm13; timm8b |
| GO:0003954 | 7 | 28 | 0.0198 | NADH dehydrogenase activity; | ndufa4; ndufs7; ndufa13; ndufa8; ndufc2; ndufa6; ndufa9 |
| GO:0050136 | 7 | 28 | 0.0198 | NADH dehydrogenase (quinone) activity; | ndufa4; ndufs7; ndufa13; ndufa8; ndufc2; ndufa6; ndufa9 |
| GO:0008137 | 7 | 28 | 0.0198 | NADH dehydrogenase (ubiquinone) activity; | ndufa4; ndufs7; ndufa13; ndufa8; ndufc2; ndufa6; ndufa9 |
| GO:0005777 | 10 | 56 | 0.0239 | peroxisome; | trim37; acaa1; pxmp2; ephx2; isoc1; pex3; idh1; decr2; hsd17b4; scp2 |
| GO:0009055 | 14 | 107 | 0.0269 | electron carrier activity; | ndufa13; ndufc2; ndufa9; fxn; akr1a1; ndufa4; ptges2; uqcr; ndufs7; ndufa8; nqo1; ndufa6; cycs; abce1 |
| GO:0006767 | 6 | 22 | 0.0269 | water-soluble vitamin metabolic process; | akr1a1; psat1; aldh9a1; pgd; tpk1; pnpo |
| GO:0042775 | 7 | 30 | 0.0269 | organelle ATP synthesis coupled electron transport; | ndufa4; ndufs7; uqcr; ndufa8; ndufc2; ndufa6; ndufa9 |
| GO:0042773 | 7 | 30 | 0.0269 | oxidative phosphorylation#ATP synthesis coupled electron transport; | ndufa4; ndufs7; uqcr; ndufa8; ndufc2; ndufa6; ndufa9 |
| GO:0016628 | 4 | 10 | 0.0405 | oxidoreductase activity, acting on the CH-CH group of donors, NAD or NADP as acceptor; | decr1; decr2; blvrb; akr1c3 |
