# Supplementary material for: Network Analysis of Breast Cancer Progression and Reversal Using a Tree-Evolving Network Algorithm
Source: PLoS Comput Biol. 2014 Jul 24;10(7):e1003713. doi: 10.1371/journal.pcbi.1003713 (PMC4109850; doi:10.1371/journal.pcbi.1003713)
Supplement: Table S3 — Diseases significantly associated with the genes in the differential networks of the breast cell states in the progression and reversion model of the HMT3522 cells. (A) S1 differential network; (B) T4-2 differential network; (C) EGFR/ITGB1-T4R differential network; (D) PI3K/MAPKK-T4R differential network; (E) MMP-T4R differential network. (DOCX) [file pcbi.1003713.s010.docx]

Table S3: Diseases significantly associated with the genes in the differential networks of the breast cell states in the progression and reversion model of the HMT3522 cells.

(A) Diseases significantly associated with the genes in the S1 differential network.

None.

(B) Diseases significantly associated with the genes in the T4 differential network.

| KEGGID | Term | Pvalue | pFDR | Odds Ratio | Exp Count | Count | Size | Genes |
| --- | --- | --- | --- | --- | --- | --- | --- | --- |
| 5142 | Chagas disease (American trypanosomiasis) | 2.00E-05 | 0.002 | 3.04 | 9.77 | 24 | 97 | FAS, BDKRB2, FOS, GNA15, GNAI3, GNAS, IL8, JUN, SMAD3, NFKB1, SERPINE1, PIK3R1, PPP2R2A, RELA, CCL5, MAP2K4, TGFB1, TGFBR1, TLR2, TRAF6, PIK3R3, FADD, TLR6, TICAM1 |
| 5146 | Amoebiasis | 6.90E-05 | 0.0035 | 2.77 | 10.47 | 24 | 104 | ACTN1, ARG2, CASP3, CD14, COL4A1, COL4A2, CSF2, FN1, GNA15, GNAS, CXCL1, IL8, LAMA3, LAMB3, LAMC2, NFKB1, SERPINB2, SERPINB6, PIK3R1, RAB5A, RELA, TGFB1, TLR2, PIK3R3 |
| 5200 | Pathways in cancer | 7.30E-05 | 0.0035 | 1.94 | 30.81 | 52 | 306 | FAS, BMP2, BMP4, CASP3, CCNE1, CDK2, CKS1B, COL4A1, COL4A2, DAPK3, DVL1, EPAS1, ETS1, FGF1, FN1, FOS, GSK3B, HRAS, IL8, ITGA6, ITGA2, ITGAV, JUN, LAMA3, LAMB3, LAMC2, SMAD3, MET, MYC, NFKB1, PIK3R1, PLD1, MAP2K1, PTGS2, RAC1, RAC2, RALA, RELA, SOS2, TGFA, TGFB1, TGFBR1, TRAF5, TRAF6, VEGFA, VEGFC, CCDC6, FZD1, FZD7, PIK3R3, FADD, EGLN3 |
| 5211 | Renal cell carcinoma | 2.00E-04 | 0.0067 | 3.18 | 6.65 | 17 | 66 | EPAS1, ETS1, HRAS, JUN, MET, PAK1, PIK3R1, MAP2K1, RAC1, RAP1B, SOS2, TGFA, TGFB1, VEGFA, VEGFC, PIK3R3, EGLN3 |
| 5222 | Small cell lung cancer | 2.10E-04 | 0.0067 | 2.83 | 8.56 | 20 | 85 | CCNE1, CDK2, CKS1B, COL4A1, COL4A2, FN1, ITGA6, ITGA2, ITGAV, LAMA3, LAMB3, LAMC2, MYC, NFKB1, PIK3R1, PTGS2, RELA, TRAF5, TRAF6, PIK3R3 |
| 5130 | Pathogenic Escherichia coli infection | 5.20E-04 | 0.0126 | 3.49 | 4.73 | 13 | 47 | ACTB, ACTG1, CD14, KRT18, TUBA4A, TUBB2A, NCK2, ARHGEF2, ARPC1B, TUBAL3, ARPC5L, TUBB6, TUBB |
| 5120 | Epithelial cell signaling in Helicobacter pylori infection | 2.40E-03 | 0.0447 | 2.71 | 6.14 | 14 | 61 | ADAM10, CASP3, HBEGF, CXCL1, IL8, JUN, MET, NFKB1, PAK1, RAC1, RELA, CCL5, MAP2K4, JAM2 |
| 5212 | Pancreatic cancer | 2.50E-03 | 0.0447 | 2.58 | 6.85 | 15 | 68 | SMAD3, NFKB1, PIK3R1, PLD1, MAP2K1, RAC1, RAC2, RALA, RELA, TGFA, TGFB1, TGFBR1, VEGFA, VEGFC, PIK3R3 |
| 5210 | Colorectal cancer | 6.60E-03 | 0.0747 | 2.46 | 6.14 | 13 | 61 | CASP3, FOS, GSK3B, JUN, SMAD3, MYC, PIK3R1, MAP2K1, RAC1, RAC2, TGFB1, TGFBR1, PIK3R3 |

(C) Diseases associated with the genes in the EGFR/ITGB1-T4R differential network.

| KEGGID | Term | Pvalue | pFDR | Odds Ratio | Exp Count | Count | Size | Genes |
| --- | --- | --- | --- | --- | --- | --- | --- | --- |
| 5221 | Acute myeloid leukemia | 0.0091 | N.S. | 2.82 | 3.631 | 9 | 56 | AKT1, ARAF, CEBPA, JUP, KRAS, NFKB1, PIK3R2, TCF7L2, PIM2 |
| 5219 | Bladder cancer | 0.015 | N.S. | 3.02 | 2.658 | 7 | 41 | ARAF, CDH1, CDKN1A, TYMP, FGFR3, KRAS, RPS6KA5 |
| 5160 | Hepatitis C | 0.0252 | N.S. | 1.91 | 7.91 | 14 | 122 | AKT1, ARAF, CDKN1A, EIF2S1, KRAS, NFKB1, NFKBIA, OAS1, OAS2, OAS3, PIK3R2, TNFRSF1A, CLDN1, EIF2AK1 |
| 5200 | Pathways in cancer | 0.0374 | N.S. | 1.5 | 19.84 | 28 | 306 | ABL1, AKT1, ARAF, RHOA, CDH1, CDKN1A, CEBPA, COL4A4, CSF1R, DVL3, FGFR3, GSTP1, JUP, KRAS, LAMA5, LAMB2, NFKB1, NFKBIA, PIK3R2, RALGDS, TCF7L2, TRAF1, FZD5, NCOA4, RALBP1, LAMB4, CYCS, WNT5B |
| 5120 | Epithelial cell signaling in Helicobacter pylori infection | 0.0416 | N.S. | 2.21 | 3.955 | 8 | 61 | ATP6AP1, NFKB1, NFKBIA, TJP1, ATP6V0D1, NOD1, GIT1, ATP6V1D |
| 5210 | Colorectal cancer | 0.0416 | N.S. | 2.21 | 3.955 | 8 | 61 | AKT1, ARAF, RHOA, KRAS, PIK3R2, RALGDS, TCF7L2, CYCS |
| 5215 | Prostate cancer | 0.0436 | N.S. | 1.98 | 5.446 | 10 | 84 | AKT1, ARAF, CDKN1A, GSTP1, KRAS, NFKB1, NFKBIA, PIK3R2, TCF7L2, CREB3L2 |
| 5222 | Small cell lung cancer | 0.0467 | N.S. | 1.96 | 5.511 | 10 | 85 | AKT1, COL4A4, LAMA5, LAMB2, NFKB1, NFKBIA, PIK3R2, TRAF1, LAMB4, CYCS |

(D) Diseases associated with the genes in the PI3K/MAPKK-T4R differential network.

| KEGGID | Term | Pvalue | pFDR | Odds Ratio | Exp Count | Count | Size | Genes |
| --- | --- | --- | --- | --- | --- | --- | --- | --- |
| 5160 | Hepatitis C | 5.50E-03 | N.S. | 2.01 | 11.717 | 21 | 122 | BAD, CHUK, MAPK14, EGFR, IFIT1, JAK1, OAS1, OAS2, OAS3, PDK1, PIK3CA, PIK3CD, PIK3R2, EIF2AK2, TNF, TNFRSF1A, TYK2, PIAS1, PIAS2, CLDN1, DDX58 |
| 5212 | Pancreatic cancer | 1.10E-02 | N.S. | 2.26 | 6.531 | 13 | 68 | BAD, CCND1, CHUK, EGFR, JAK1, PIK3CA, PIK3CD, PIK3R2, PLD1, RALA, VEGFA, VEGFB, RALBP1 |
| 5142 | Chagas disease (American trypanosomiasis) | 4.20E-02 | N.S. | 1.75 | 9.316 | 15 | 97 | CHUK, MAPK14, GNAI1, IFNGR2, IRAK1, MYD88, PIK3CA, PIK3CD, PIK3R2, CCL2, CCL5, MAP2K4, TNF, TNFRSF1A, CFLAR |
| 5332 | Graft-versus-host disease | 4.60E-02 | N.S. | 2.59 | 2.689 | 6 | 28 | HLA-A, HLA-C, HLA-E, HLA-F, HLA-G, TNF |

(E) Diseases significantly associated with the genes in the MMP-T4R differential network.

| KEGGID | Term | Pvalue | pFDR | Odds Ratio | Exp Count | Count | Size | Genes |
| --- | --- | --- | --- | --- | --- | --- | --- | --- |
| 5016 | Huntington's disease | 3.00E-04 | 0.0169 | 2.66 | 8.597 | 20 | 150 | AP2B1, SLC25A5, ATP5G1, CASP8, HDAC1, NDUFA4, NDUFA6, NDUFA8, NDUFA9, POLR2K, POLR2L, TP53, UQCRFS1, UQCR11, UQCRQ, NDUFA13, CYCS, IFT57, CREB3L2, NDUFS7 |
| 5010 | Alzheimer's disease | 3.40E-04 | 0.0169 | 2.71 | 8.024 | 19 | 140 | APP, ATP5G1, CASP8, CDK5, IDE, NDUFA4, NDUFA6, NDUFA8, NDUFA9, PPP3R1, SNCA, UQCRFS1, EIF2AK3, UQCR11, UQCRQ, NDUFA13, CYCS, PSENEN, NDUFS7 |
| 5012 | Parkinson's disease | 1.20E-03 | 0.0352 | 2.84 | 5.617 | 14 | 98 | SLC25A5, ATP5G1, NDUFA4, NDUFA6, NDUFA8, NDUFA9, SNCA, UBE2G2, UQCRFS1, UQCR11, UQCRQ, NDUFA13, CYCS, NDUFS7 |
