# Supplementary material for: Network Analysis of Breast Cancer Progression and Reversal Using a Tree-Evolving Network Algorithm
Source: PLoS Comput Biol. 2014 Jul 24;10(7):e1003713. doi: 10.1371/journal.pcbi.1003713 (PMC4109850; doi:10.1371/journal.pcbi.1003713)
Supplement: Table S4 — Hubs in the differential networks of the breast cell states significantly affecting survival of breast cancer patients. (DOCX) [file pcbi.1003713.s011.docx]

Table S4: Hubs in the differential networks of the breast cell states significantly affecting survival of breast cancer patients.

| **Gene** | **15-year Survival Rate (%)** | **Description** | **p-value**  **(BH FDR)** | **Cell State** | **PDN Degree** | **Total Degree** |
| --- | --- | --- | --- | --- | --- | --- |
| PLTP | 52 | phospholipid transfer protein | 0.011 | S1 | 27 | 65 |
| DCTN6 | 47 | dynactin 6 | 0.011 | S1 | 25 | 34 |
| PRKX | 43 | protein kinase, X-linked | 0.011 | S1 | 22 | 57 |
| NEBL | 36 | nebulette | 0.021 | S1 | 86 | 154 |
| ALOX15B | 55 | arachidonate 15-lipoxygenase, type B | 0.037 | S1 | 36 | 64 |
| HYOU1 | 53 | hypoxia up-regulated 1 | 0.040 | S1 | 101 | 242 |
| GCLM | 41 | glutamate-cysteine ligase, modifier subunit | 0.003 | T4 | 24 | 56 |
| HBEGF | 34 | heparin-binding EGF-like growth factor | 0.007 | T4 | 73 | 106 |
| TXNIP | 50 | thioredoxin interacting protein | 0.011 | T4 | 28 | 58 |
| PTHLH | 45 | parathyroid hormone-like hormone | 0.011 | T4 | 18 | 22 |
| NMB | 50 | neuromedin B | 0.013 | T4 | 24 | 44 |
| SPHK1 | 54 | sphingosine kinase 1 | 0.024 | T4 | 88 | 156 |
| SYN3 | 49 | synapsin III | 0.011 | EGFR/ITGB1-T4R | 120 | 181 |
| EBP | 35 | emopamil binding protein (sterol isomerase) | 5.5E-06 | PI3K/MAPKK-T4R | 13 | 40 |
| CHI3L1 | 47 | chitinase 3-like 1 (cartilage glycoprotein-39) | 0.011 | PI3K/MAPKK-T4R | 205 | 453 |
| CDKN2AIP | 42 | CDKN2A interacting protein | 0.011 | PI3K/MAPKK-T4R | 20 | 39 |
| SGK3 | 53 | serum/glucocorticoid regulated kinase family, member 3 | 0.011 | PI3K/MAPKK-T4R | 26 | 40 |
| PER3 | 42 | period homolog 3 (Drosophila) | 0.024 | PI3K/MAPKK-T4R | 17 | 31 |
| PAPD7 | 30 | polymerase (DNA directed) sigma | 9.9E-07 | MMP-T4R | 89 | 118 |
| SLC7A6 | 47 | solute carrier family 7 (cationic amino acid transporter, y+ system), member 6 | 0.024 | MMP-T4R | 29 | 42 |
| HNRNPH1 | 45 | heterogeneous nuclear ribonucleoprotein H1 (H) | 0.025 | MMP-T4R | 7 | 9 |
| NIT2 | 51 | nitrilase family, member 2 | 0.040 | MMP-T4R | 62 | 111 |
